# Perinatal outcomes associated with antiretroviral therapy for pregnant women living with HIV: an umbrella review

**DOI:** 10.1016/j.eclinm.2026.103919

**Published:** 2026-05-07

**Authors:** Pippa Huete Boering, Joris Hemelaar

**Affiliations:** Infectious Disease Epidemiology Unit, Nuffield Department of Population Health, University of Oxford, Oxford, UK

**Keywords:** HIV, Antiretroviral therapy, Pregnancy, Preterm birth, Small for gestational age, Low birthweight

## Abstract

**Background:**

The World Health Organization recommends antiretroviral therapy (ART) for pregnant women living with HIV (WLHIV), the vast majority of whom reside in sub-Saharan Africa. In recent years, many systematic reviews, meta-analyses, and randomised controlled trials (RCTs) have been performed to assess the risks of adverse perinatal outcomes associated with ART among WLHIV. The aim of this umbrella review is to assess the available evidence regarding the risks of adverse perinatal outcomes for WLHIV receiving ART.

**Methods:**

We conducted a systematic literature review by searching Medline, Global Health, and EMBASE and two clinical trial databases (ClinicalTrials.gov and the WHO International Clinical Trials Registry Platform) for meta-analyses and RCTs published between 1 January 1980 and 12 February 2026. We included meta-analyses and RCTs reporting on the association of pregnant WLHIV receiving ART with perinatal outcomes, compared to WLHIV receiving different ART regimens, WLHIV naïve to ART, and women without HIV. We also included studies that assessed the timing of ART initiation (preconception or antenatal). Twelve predefined perinatal outcomes were assessed: preterm birth (PTB), very PTB (VPTB), spontaneous PTB (sPTB), low birthweight (LBW), very LBW (VLBW), term and preterm LBW, small for gestational age (SGA), very SGA (VSGA), stillbirth, neonatal death, and vertical HIV transmission. Heterogeneity measures (*I*^*2*^) and Peter's test results for publication bias were extracted and assessed. The quality of meta-analyses was assessed using the AMSTAR 2 tool, risk of bias of RCTs was assessed using the Cochrane Risk of Bias tool, and overall certainty of evidence for each exposure comparison and perinatal outcome was assessed according to GRADE. The protocol is registered with PROSPERO, number CRD42021248987.

**Findings:**

Of 14,279 studies identified, we included nine meta-analyses of cohort studies, one network meta-analysis of seven RCTs, and three additional RCTs. The meta-analyses were composed of a total of 154 cohort studies and were of low or critically low quality, and RCTs had a high risk of bias. Meta-analyses of cohort studies found that WLHIV receiving ART are at increased risks of PTB (risk ratio (RR) 1.55, 95% confidence interval (CI) 1.38–1.74, *I*^*2*^ 87.4%), sPTB (RR 2.10, 95% CI 1.48–2.96, *I*^*2*^ 12.5%), LBW (RR 1.79, 95% CI 1.51–2.13, *I*^*2*^ 90.6%), term LBW (RR 1.88, 95% CI 1.23–2.85, *I*^*2*^ 0.0%), SGA (RR 1.80, 95% CI 1.34–2.40, *I*^*2*^ 97.6%), and VSGA (RR 1.22, 95% CI 1.10–1.34, *I*^*2*^ 0.0%), compared to HIV-negative women. RCTs comparing ART regimens among WLHIV found few differences in perinatal outcomes assessed. Meta-analyses of cohort studies comparing different ART regimens found that protease inhibitors are associated with an increased risk of SGA (RR 1.28, 95% CI 1.09–1.51, *I*^*2*^ 62.2%) and VSGA (RR 1.41, 95% CI 1.08–1.83, *I*^*2*^ 0.0%), compared to non-nucleoside reverse transcriptase inhibitors. Tenofovir disoproxil fumarate is associated with a lower risk of adverse perinatal outcomes and zidovudine is associated with an increased risk of perinatal outcomes. Compared to HIV-negative women, WLHIV receiving ART remain at increased risk of adverse perinatal outcomes, irrespective of the ART regimen and timing of ART initiation. Publication bias, as determined using the Peter's test, was found for two analyses. Most findings were of low or very low certainty as assessed using GRADE.

**Interpretation:**

WLHIV receiving ART are associated with increased risks of adverse perinatal outcomes compared to HIV-negative women, irrespective of the ART regimen and timing of ART initiation. To strengthen the evidence base for ART guidelines for pregnant WLHIV, more and larger RCTs and high quality observational studies are needed to optimise ART regimens in pregnancy. Further efforts should be made to improve perinatal outcomes among WLHIV.

**Funding:**

This study received no funding.

**Research in context**

**Evidence before this study**

The vast majority of pregnant women living with HIV (WLHIV) reside in sub-Saharan Africa, which also has the highest neonatal mortality rates. Untreated maternal HIV infection is associated with adverse perinatal outcomes, such as preterm birth, low birthweight, and small for gestational age. The World Health Organization recommends antiretroviral therapy (ART) for pregnant WLHIV to reduce maternal morbidity and mortality and vertical HIV transmission. However, the impact of ART on perinatal outcomes of WLHIV has been uncertain, due to the complexities of triple drug ART regimens, differences in exposure comparators between studies, variation in definitions of perinatal outcomes, and the limited number of studies, especially randomised controlled trials (RCTs), conducted in pregnant WLHIV. A systematic search of Medline, EMBASE, Global Health, and clinical trial registries using “HIV”, “perinatal outcomes” and “antiretroviral therapy (ART)” for studies published since 1 January 1980 was conducted on 5 March 2025, and updated on 12 February 2026. The search yielded 14,279 studies including several systematic reviews, meta-analyses, and RCTs that assessed the risks of adverse perinatal outcomes associated with ART among WLHIV. However, an overview of all the available evidence regarding the risks of perinatal outcomes for pregnant WLHIV receiving ART has been lacking.

**Added value of this study**

To fill this evidence gap, we conducted the first umbrella review of meta-analyses and RCTs reporting on the association of pregnant WLHIV receiving ART with 12 predefined perinatal outcomes, published between 1 January 1980 and 12 February 2026. We included nine meta-analyses of cohort studies, one network meta-analysis of seven RCTs, and three additional RCTs. We find that WLHIV receiving ART are at increased risk of adverse perinatal outcomes, including preterm birth, low birthweight, and small for gestational age, compared to HIV-negative women. RCTs comparing ART regimens among WLHIV found few differences in perinatal outcomes assessed. Meta-analyses of cohort studies comparing different ART regimens indicate that protease inhibitors are associated with an increased risk of small for gestational age and very small for gestational age, compared to non-nucleoside reverse transcriptase inhibitors. Moreover, tenofovir disoproxil fumarate is associated with a lower risk of adverse perinatal outcomes and zidovudine is associated with an increased risk of adverse perinatal outcomes. Importantly, meta-analyses of cohort studies indicate that WLHIV receiving ART, compared to HIV-negative women, remain at increased risk of adverse perinatal outcomes, irrespective of the ART regimen and timing of ART initiation. The main limitations are the limited number of small RCTs, which were at high risk of bias, and the low quality of the meta-analyses. GRADE assessments indicated low or very low certainty of the evidence for all perinatal outcomes.

**Implications of all the available evidence**

ART in pregnancy has clear benefits for WLHIV to improve their health and to prevent vertical HIV transmission to their baby. However, current evidence indicates that WLHIV receiving ART are at increased risk of adverse perinatal outcomes compared to HIV-negative women, irrespective of the ART regimen and timing of ART initiation. To strengthen the evidence base for ART guidelines for pregnant WLHIV, more and larger RCTs and high-quality observational studies are needed to determine the optimal ART regimens in pregnancy. Further efforts should be made to improve perinatal outcomes among WLHIV, including improving access to ART and further research to develop a greater understanding of the mechanisms that lead to adverse perinatal outcomes among WLHIV, which may inform preventative and therapeutic interventions.

## Introduction

Globally, 40.8 million people worldwide were living with HIV in 2024, including 15.7 million women of childbearing age.^1^ Each year, 1.1 million women living with HIV (WLHIV) are pregnant, with the vast majority living in sub-Saharan Africa.^2^ Since 2013, the World Health Organization (WHO) recommends that pregnant WLHIV receive antiretroviral therapy (ART), as it improves their health and prevents vertical transmission of HIV.^3^ Furthermore, the WHO adoption in 2015 of a ‘treat all’ approach with immediate ART initiation after diagnosis has resulted in an increasing proportion of WLHIV starting ART prior to conception.^4^ Implementation of these recommendations has led to an increase in the proportion of pregnant WLHIV receiving ART globally from 44% in 2010 to 84% in 2024, leading to a decline in perinatal HIV transmission.^2^

A wide range of ART drugs and combinations are available, and recommendations for ART regimens during pregnancy vary by country and have changed over time. ART regimens consist of a backbone of two nucleoside reverse transcriptase inhibitors (NRTIs) combined with a ‘third drug,’ including integrase strand transfer inhibitors (INSTIs), non-nucleoside reverse transcriptase inhibitors (NNRTIs), or protease inhibitors (PIs). The WHO currently recommends INSTI dolutegravir (DTG)-based ART as first-line regimen for adults, including pregnant women.^5^ NNRTI efavirenz (EFV)-based ART is an alternative first-line regimen. ART containing PIs, preferably ritonavir-boosted darunavir (DRV/r), with ritonavir-boosted atazanavir (ATV/r) and ritonavir-boosted lopinavir (LPV/r) as alternatives, are designated as second-line or third-line regimens. Tenofovir (tenofovir disoproxil fumarate (TDF) or tenofovir alafenamide fumarate (TAF)) combined with lamivudine (3TC) (or emtricitabine (FTC)) is the preferred NRTI backbone.^5^ US guidelines recommend INSTI DTG-based or bictegravir (BIC)-based ART, with DRV/r-based ART as an alternative in pregnancy. The preferred backbone consists of TAF or TDF combined with FTC or 3TC.^6^ European guidelines recommend DTG-based ART or DRV/r-based ART in pregnancy, with a backbone of TAF or TDF combined with FTC or 3TC. Alternatives include raltegravir (RAL)-based or BIC-based ART, and EFV-based ART regimens.^7^ Finally, UK guidelines recommend DTG-based ART regimens with an FTC/TDF (or FTC/TAF or 3TC/abacavir (ABC)) backbone as first line in pregnancy, with rilpivirine (RPV)-based, RAL-based, DRV/r-based, and EFV-based ART regimens as alternatives.^8^

Maternal HIV infection is associated with an increased risk of adverse perinatal outcomes, including preterm birth (PTB), low birthweight (LBW), and small for gestational age (SGA).^9^ The mechanisms through which HIV contributes to adverse perinatal outcomes remain poorly understood, however hypotheses include placental lesions, chronic inflammation and immune dysregulation.^10^, ^11^, ^12^, ^13^ As the number of pregnant WLHIV receiving ART increases, understanding of the impact of different ART regimens on perinatal outcomes is crucial. Globally, PTB is the most important cause of neonatal and child mortality,^14^ with an estimated 13.4 million cases in 2020, with no change in the global preterm birth rate since 2010.^15^ An estimated 23.4 million newborns were SGA in 2020.^16^ Both PTB and SGA contribute to LBW, with an estimated 19.8 million LBW cases in 2020.^17^ It has been estimated that almost 2 million preterm births, and LBW and SGA babies in sub-Saharan Africa were attributable to HIV and ART during the period 1990–2020.^18^ The United Nations’ Sustainable Development Goal 3 (SDG3) target 3.2 aims to reduce neonatal and under-5 mortality to 12 and 25 per 1000 live births, respectively, by 2030.^19^ Sub-Saharan Africa has the highest rates of neonatal and child mortality globally and these SDG3 targets are predicted to be missed by most countries in this region.^20^ Addressing adverse perinatal outcomes in regions with high maternal HIV prevalence and neonatal and child mortality is therefore urgently needed.

The impact of ART on perinatal outcomes of WLHIV, especially PTB, has long been uncertain and controversial.^21^ The assessment of the association of ART with adverse perinatal outcomes has been complicated by a number of factors, including i) the complexities of triple drug ART regimens, which have evolved over time; ii) the different exposure comparators used in different studies, including WLHIV receiving different ART regimens, WLHIV who did not receive any ART or received zidovudine (ZDV) monotherapy, or HIV-negative women; iii) variation in the definitions, or lack of definition, of perinatal outcomes assessed in different studies; iv) and the limited number and small sizes of randomised controlled trials (RCTs) conducted in pregnant WLHIV. A number of reviews have sought to assess the evidence regarding pregnancy outcomes of WLHIV receiving ART.^21^, ^22^, ^23^, ^24^, ^25^, ^26^, ^27^, ^28^, ^29^, ^30^, ^31^, ^32^, ^33^ However, many of these reviews had limitations, such as inclusion of mixtures of mono- and dual therapy and ART (triple therapy),^31^ lack of a comparator exposure group,^29^^,^^30^ alternative or lack of definitions of perinatal outcomes,^25^^,^^32^ or lack of a meta-analysis.^21^, ^22^, ^23^, ^24^^,^^26^, ^27^, ^28^^,^^33^

International HIV treatment guidelines highlight the limited data available regarding the safety and pregnancy outcomes associated with ART drugs in pregnancy.^5^, ^6^, ^7^, ^8^ To help fill this evidence gap, we conducted the first umbrella review of meta-analyses, as well as RCTs, reporting on the association of pregnant WLHIV receiving ART with 12 predefined perinatal outcomes, compared to WLHIV receiving different ART regimens, WLHIV naïve to ART, and women without HIV. In addition, we examined studies that assessed the timing of ART initiation (preconception or antenatal).

## Methods

### Search strategy

The systematic review protocol was developed in accordance with the Cochrane guidelines,^34^ and registered online (PROSPERO, number CRD42021248987). This umbrella review is reported according to the Preferred Reporting Items for Systematic Reviews and Meta-Analyses (PRISMA) and Preferred Reporting Items for Overviews of Reviews (PRIOR) guidelines.^35^^,^^36^ A comprehensive literature search strategy was developed and adapted to three electronic literature databases (Medline (Ovid), Global Health (Ovid), Embase (Ovid)) to search for studies published between 1 January 1980 and 5 March 2025. An updated literature search was conducted on 12 February 2026, which yielded no additional studies that were eligible for inclusion. Our search time window was set from 1980, prior to the introduction of ART, to enable inclusion of studies which reported perinatal outcomes for WLHIV without ART compared to HIV-negative women. Separate searches using the search strategy were conducted for meta-analyses and RCTs. In addition, we searched two clinical trial databases (ClinicalTrials.gov and the WHO International Clinical Trials Registry Platform). We used free text and controlled vocabulary search terms for “HIV”, “antiretroviral therapy”, and “pregnancy outcome”, as well as “meta-analysis” or “randomised controlled trial”. No restrictions for country or language were applied. The full search terms are included in Appendix pp 3–7. Both full-text articles and abstracts were considered. The references of included studies were assessed for additional relevant studies. Retrieved citations were imported into EndNote reference manager (EndNote ×21, Clarivate Analytics, Philadelphia, Pennsylvania, USA) and deduplicated.

### Study selection and eligibility criteria

Meta-analyses and RCTs were eligible if they contained information regarding the association of pregnant WLHIV receiving ART with predefined adverse perinatal outcomes. Titles and abstracts were screened, and full text manuscripts of selected references were assessed against the eligibility criteria by two reviewers (PB and JH). Inclusion criteria were study design (meta-analysis conducted according to PRISMA guidelines or RCT), study population (pregnant women), exposures (WLHIV receiving ART) and comparators (WLHIV receiving different ART regimens, WLHIV naïve to ART, and women without HIV). To provide a comprehensive overview of available evidence, as well as to prevent overlap of primary data, we included primary RCTs which were not included in the network meta-analysis of RCTs.^37^ In addition, we included studies that compared WLHIV naïve to ART with women without HIV, and studies that assessed the timing of ART initiation (preconception or antenatal). ART was defined as any triple drug antiretroviral therapy. We excluded studies that used WLHIV receiving monotherapy or dual therapy as an exposure or comparator group.

Perinatal outcomes were defined as follows: preterm birth (PTB, birth <37^+^^0^ weeks gestation)^15^; very PTB (VPTB, birth <32^+^^0^ weeks gestation); spontaneous PTB (sPTB, spontaneous birth <37^+^^0^ weeks gestation); low birthweight (LBW, <2500 g)^17^; very LBW (VLBW, <1500 g); small for gestational age (SGA, birth-weight for gestational age <10^th^ centile)^38^^,^^39^ or very SGA (VSGA, birthweight for gestational age <3^rd^ centile) according to the reference chart used at the study site, and stillbirth (SB, delivery of an infant without any signs of life with birthweight at least 1000 g or gestational age ≥24^+^^0^ weeks or body length ≥35 cm); neonatal death (NND, death of an infant in the first 28 days of life); and vertical HIV transmission. Term and preterm LBW were defined according to definitions of PTB and LBW. Studies and data were not included if ART or perinatal outcomes were undefined or not defined in line with our definitions. Differences in opinion regarding inclusion of studies were resolved through discussion among the authors. Details of excluded papers and reasons for exclusion can be found in Appendix pp 41–57.

### Data extraction

Data on study characteristics (including numbers of studies, participants, and countries), study populations, ART regimens and timing of ART initiation of WLHIV, and perinatal outcomes were extracted from eligible studies by one investigator (PB) and reviewed by the senior investigator (JH). Results from meta-analyses, including risk ratios (RRs), 95% confidence intervals (CIs), heterogeneity (*I*^*2*^) and Peter's test results were extracted for each exposure comparison and perinatal outcome. For RCTs, any available RRs and 95% CI were extracted for each comparison and perinatal outcome. Raw outcome data (frequencies) were extracted when these were not available.

### Statistical analysis

In post-hoc analyses, we calculated risk ratios and 95% CIs using raw frequency data if outcome frequencies or alternative relative risk measures (e.g. risk difference) were reported, using STATA version 18 (College Station, Texas, USA). In additional post-hoc analyses, overall as well as study-pair specific corrected covered areas were calculated as described by Hennessy and Johnson.^40^

### Quality assessment

The meta-analyses included in this umbrella review were assessed for methodological quality using the A Measurement Tool to Assess systematic Reviews, version 2 (AMSTAR 2) tool by two independent assessors (PB and JH). The AMSTAR 2 tool is composed of 16 domains examining study selection, data extraction, risk of bias, and synthesis and discussion of results.^41^ Further details regarding the AMSTAR 2 tool, full results of the assessments, and criteria for overall quality of each meta-analysis can be found in Appendix pp 10–12. The quality of RCTs included in this study was assessed using the Cochrane Risk of Bias tool, version 1 (RoB1).^42^ The RoB1 tool assesses RCTs across six domains: sequence generation, allocation concealment, blinding, incomplete outcome data, selective outcome reporting, and other sources of bias. Details of the RoB1 assessments and criteria for overall risk of bias of each RCT are included in Appendix pp 8 and 9. Grading of Recommendations Assessment, Development and Evaluation (GRADE) assessments of certainty of evidence for each perinatal outcome for each exposure comparison were conducted by two independent assessors (PB and JH). The GRADE system assesses the risk of bias, inconsistency, indirectness, imprecision, and publication bias for each outcome.^43^ Publication bias was assessed using Peter's test for each outcome composed of more than ten studies and this was included as part of the GRADE assessment. Heterogeneity for meta-analyses results extracted are presented in Table 1, Table 2, Table 3, Table 4, Table 5, Table 6 and were also incorporated in the GRADE assessment of certainty of evidence. Details of criteria used to GRADE evidence certainty for meta-analyses and RCTs can be found in Appendix pp 13–15.

**Fig. 1:**
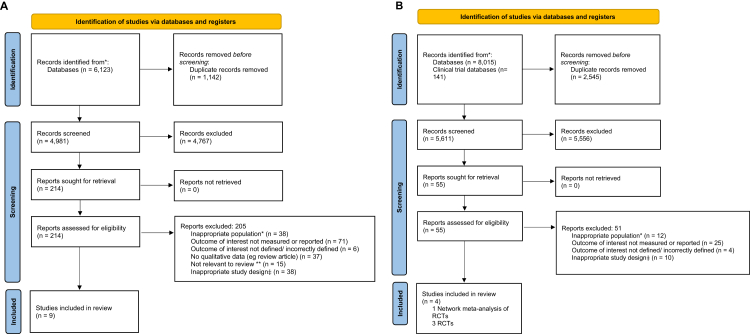
**PRISMA diagram of included studies**. A. PRISMA diagram for meta-analyses included. B. PRISMA diagram for randomised controlled trials included. ∗For example, not pregnant WLHIV. ∗∗For example, ART is Assisted Reproductive Technology. ‡For example, case series.

**Table 1:** Summary of studies included in the systematic review.

Publication	Number of studies	Number of participants	Country income status	ART regimen of WLHIV	Comparison	Perinatal outcomes	Quality of evidence^a^ (AMSTAR 2)
**Meta-analyses of cohort studies**							
Beck et al., Frontiers in Medicine, 2024^44^	30	222,312	• LMICs: 14 studies (47%), 173,456 women (78%) • HICs: 16 studies (53%), 48,856 women (22%)	• PI-based ART • NNRTI-based ART • INSTI-based ART • NRTI-based ART	• WLHIV with alternative ART regimen	• Preterm birth • Very preterm birth • Spontaneous preterm birth • Low birthweight • Very low birthweight • Small for gestational age • Very small for gestational age • Stillbirth • Neonatal death	Low
Boering et al., AIDS, 2025^45^	31	199,156	• LMICs: 25 studies (81%), 182,847 women (92%) • HICs: 6 studies (19%), 16,309 women (8%)	• Preconception ART initiation • Antenatal ART initiation	• HIV-negative women • WLHIV naïve to ART	• Preterm birth • Very preterm birth • Spontaneous preterm birth • Low birthweight • Very low birthweight • Small for gestational age • Very small for gestational age • Stillbirth • Neonatal death	Low
Cowdell et al., eClinicalMedicine, 2022^46^	34	57,546	• LMICs: 10 studies (29%), 13,932 women (24%) • HICs: 24 studies (71%), 43,661 women (76%)	• PI-based ART • Non-PI-based ART	• WLHIV with alternative ART regimen • Comparisons of individual PI-drugs	• Preterm birth • Very preterm birth • Spontaneous preterm birth • Low birthweight • Very low birthweight • Term low birthweight • Small for gestational age • Very small for gestational age • Stillbirth • Neonatal death	Low
Cowdell et al., Clinical Microbiology and Infection, 2025^47^	22	124,478	• LMICs: 9 studies (41%), 71,931 women (58%) • HICs: 13 studies (59%), 52,547 women (42%)	• TDF-based ART • ZDV-based ART • ABC-based ART • TAF-based ART • TDF+ (FTC/3TC)-ART • ZDV+ (FTC/3TC)-ART • ABC+ (FTC/3TC)-ART	• WLHIV with alternative ART regimen	• Preterm birth • Very preterm birth • Low birthweight • Very low birthweight • Term low birthweight • Small for gestational age • Very small for gestational age • Stillbirth • Neonatal death	Low
Hey et al., AIDS, 2025^48^	22	191,857	• LMICs: 15 studies (68%), 175,839 women (92%) • HICs: 7 studies (32%), 16,018 women (8%)	• INSTI-based ART • PI-based ART • NNRTI-based ART • EFV-based ART • NVP-based ART	• HIV-negative women	• Preterm birth • Very preterm birth • Spontaneous preterm birth • Low birthweight • Very low birthweight • Term low birthweight • Preterm low birthweight • Small for gestational age • Very small for gestational age • Stillbirth • Neonatal death	Low
Portwood et al., AIDS, 2022^49^	61	409,781	• LMICs: 30 studies (49%), 349,891 women (85%) • HICs: 31 studies (51%), 59,890 women (15%)	• ART	• HIV-negative women • WLHIV naïve to ART	• Preterm birth • Very preterm birth • Spontaneous preterm birth • Low birthweight • Very low birthweight • Term low birthweight • Preterm low birthweight • Small for gestational age • Very small for gestational age • Stillbirth • Neonatal death	Low
Sexton et al., HIV Medicine, 2023^50^	25	40,920	• LMICs: 13 studies (52%), 21,338 women (52%) • HICs: 12 studies (48%), 19,582 women (48%)	• Preconception ART initiation • Antenatal ART initiation	• Alternative timing of initiation of ART	• Preterm birth • Very preterm birth • Low birthweight • Very low birthweight • Small for gestational age • Very small for gestational age • Neonatal death	Low
Uthman et al., Lancet HIV, 2017^51^	11	19,189	• LMICs: 5 studies (45%), 8052 women (41%) • HICs: 6 studies (55%), 11,485 women (59%)	• Preconception ART initiation • Antenatal ART initiation	• Alternative timing of initiation of ART	• Preterm birth • Low birthweight • Very low birthweight • Small for gestational age • Very small for gestational age	Critically low
Wedi et al., Lancet HIV, 2016^9^	35	53,623	• LMICs: 28 studies (80%), 46,709 women (87%) • HICs: 7 studies (20%), 7016 women (13%)	• WLHIV naïve to ART	• HIV-negative women	• Preterm birth • Very preterm birth • Low birthweight • Very low birthweight • Term low birthweight • Preterm low birthweight • Small for gestational age • Very small for gestational age • Stillbirth • Neonatal death	Low

Publication	Trial	Number of participants	Countries	ART regimen of WLHIV	Comparison	Perinatal outcomes	Risk of bias (RoB1)^b^
**Randomised Controlled Trials**							
Joao et al., Lancet HIV, 2020^52^	NICHD P1081	408	Argentina, Brazil, South Africa, Tanzania, Thailand, USA	• ZDV/3TC/RAL • ZDV/3TC/EFV	• WLHIV with alternative ART regimen	• Preterm birth • Low birthweight • Very low birthweight • Vertical transmission	High risk
Kintu et al., Lancet HIV, 2020^53^	DolPHIN-2	268	South Africa, Uganda	• TDF/(FTC/3TC)/DTG • TDF/(FTC/3TC)/EFV	• WLHIV with alternative ART regimen	• Preterm birth • Stillbirth • Vertical transmission	High risk
Lockman et al., Lancet, 2021^54^	IMPAACT 2010/VESTED	643	Botswana, Brazil, India, South Africa, Tanzania, Thailand, Uganda, USA, Zimbabwe	• TAF/FTC/DTG • TDF/FTC/DTG • TDF/FTC/EFV	• WLHIV with alternative ART regimen	• Preterm birth • Low birthweight • Very low birthweight • Small for gestational age • Stillbirth • Neonatal death • Vertical transmission	High risk
Tshivuila-Matala et al., AIDS, 2020^37^	Network meta-analysis of seven RCTs: • PROMISE • PROMOTE • Mma Bana • Kesho Bora • PATCG 076 • ANRS 135/PRIMEVA • PHPT-5	6285	Botswana, Burkina Faso, France, India, Kenya, Malawi, South Africa, Tanzania, Thailand, Uganda, USA, Zambia, Zimbabwe	• TDF/FTC/LPV/r • ZDV/3TC/LPV/r • ZDV/3TC/EFV • ZDV/3TC/ABC	• WLHIV with alternative ART regimen • WLHIV receiving placebo	• Preterm birth • Very preterm birth • Spontaneous preterm birth • Low birthweight • Very low birthweight • Vertical transmission	Low quality (AMSTAR 2)

Meta-analyses based on cohort studies and randomised controlled trials included in the review are shown.

Abbreviations: 3TC: lamivudine, ABC: abacavir, ART: antiretroviral therapy (triple drug), DTG: dolutegravir, EFV: efavirenz, FTC: emtricitabine, HICs: high income countries, INSTI: integrase strand transfer inhibitor, NNRTI: non-nucleoside reverse transcriptase inhibitor, NRTI: nucleoside reverse transcriptase inhibitors, NVP: nevirapine, PI: protease inhibitor, LMICs: low and middle income countries, LPV/r: lopinavir/ritonavir, RAL: raltegravir, RCT: randomised controlled trial, TAF: tenofovir alafenamide, TDF: tenofovir disoproxil fumarate, WLHIV: women living with HIV, ZDV: zidovudine.

a AMSTAR2: A Measurement Tool to Assess systematic Reviews, version 2.^40^

b RoB1: Cochrane risk-of-bias tool for randomised trials.^41^

**Table 2:** Perinatal outcomes of WLHIV receiving ART, WLHIV naïve to ART, and HIV-negative women.

Publication	Comparison	Risk ratio (95% CI)										
		PTB	VPTB	sPTB	LBW	VLBW	Term LBW	Preterm LBW	SGA	VSGA	SB	NND
Wedi et al., Lancet HIV, 2016^9^	WLHIV naïve to ART vs HIV-negative women	1.63 (1.37–1.93) 22 studies 83.1%	0.99 (0.60–1.65) 1 study		1.75 (1.52–2.02) 25 studies 76.7%	4.82 (0.47–49.72) 1 study	1.64 (1.00–2.67) 7 studies 82.6%	1.66 (0.83–3.31) 3 studies 88.7%	1.64 (1.29–2.09) 7 studies 74.9%	1.28 (0.95–1.71) 1 study	1.67 (1.05–2.66) 2 studies 0.0%	1.74 (0.61–4.97) 4 studies 85.5%
	*GRADE*	*Very low*	*Very low*		*Very low*	*Very low*	*Very low*	*Very low*	*Very low*	*Very low*	*Very low*	*Very low*
Portwood et al., AIDS, 2022^49^	WLHIV with ART vs HIV-negative women	1.55 (1.38–1.74) 25 studies 87.4%	1.72 (0.75–3.96) 5 studies 92.0%	2.10 (1.48–2.96) 3 studies 12.5%	1.79 (1.51–2.13) 15 studies 90.6%	1.70 (0.63–4.57) 5 studies 83.5%	1.88 (1.23–2.85) 3 studies 0.0%	1.17 (0.64–2.14) 1 study	1.80 (1.34–2.40) 19 studies 97.6%	1.22 (1.10–1.34) 5 studies 0.0%	0.88 (0.34–2.32) 1 study	1.27 (0.75–2.15) 5 studies 61.7%
	*GRADE*	*Very low*	*Very low*	*Very low*	*Very low*	*Very low*	*Very low*	*Very low*	*Very low*	*Low*	*Very low*	*Very low*
	WLHIV with ART vs WLHIV naïve to ART	0.85 (0.66–1.10) 20 studies 93.9%	0.89 (0.23–3.51) 2 studies 86.6%	0.46 (0.32–0.67) 2 studies 0.0%	0.92 (0.79–1.08) 15 studies 73.8%	0.36 (0.16–0.78) 1 study			1.38 (1.09–1.75) 5 studies 38.5%	2.35 (1.60–3.46) 1 study		
	*GRADE*	*Very low*	*Very low*	*Very low*	*Very low*	*Very low*			*Low*	*Very low*		

The risk ratio, 95% confidence interval (95% CI), number of studies, and *I*^*2*^ (%) of each perinatal outcome in comparisons of WLHIV receiving ART, WLHIV naïve to ART, and HIV-negative women in meta-analyses of cohort studies are shown. GRADE (Grading of Recommendations Assessment, Development and Evaluation) assessments of certainty of evidence for each comparison and perinatal outcome are included.

A risk ratio >1 indicates an increased risk of a perinatal outcome associated with the first-mentioned comparator group compared to the second-mentioned group. For example, WLHIV naïve to ART are associated with an increased risk of PTB compared to HIV negative women (RR 1.63, 95% CI 1.37–1.93).

Abbreviations: ART: antiretroviral therapy (triple drug), LBW: low birthweight, NND: neonatal death, PTB: preterm birth, SB: stillbirth, SGA: small for gestational age, sPTB: spontaneous preterm birth, VLBW: very low birthweight, VPTB: very preterm birth, VSGA: very small for gestational age, WLHIV: women living with HIV.

**Table 3:** Perinatal outcomes of WLHIV receiving different ART classes in meta-analyses of cohort studies.

Publication	Comparison	Risk ratio (95% CI)										
		PTB	VPTB	sPTB	LBW	VLBW	Term LBW	Preterm LBW	SGA	VSGA	SB	NND
**WLHIV receiving different ART classes compared toHIV-negativewomen**												
Hey et al., AIDS, 2025^48^	WLHIV with PI-ART vs HIV-negative women	1.88 (1.55–2.28) 9 studies 64.8%	2.06 (1.01–4.18) 4 studies 73.4%	16.96 (1.01–284.08) 1 study	2.90 (2.41–3.50) 3 studies 1.7%	4.35 (2.67–7.09) 2 studies 0.0%	1.83 (0.88–3.80) 1 study		2.92 (0.95–9.00) 6 studies 98.1%	2.37 (1.84–3.05) 2 studies 0.0%		1.82 (0.71–4.69) 2 studies 11.8%
	*GRADE*	*Very low*	*Very low*	*Very low*	*Very low*	*Very low*	*Very low*		*Very low*	*Very low*		*Very low*
	WLHIV with NNRTI-ART vs HIV-negative women	1.40 (1.27–1.56) 15 studies 87.3%	1.94 (1.25–3.01) 6 studies 88.5%		1.63 (1.30–2.04) 9 studies 90.0%	2.43 (0.76–7.78) 4 studies 86.6%	1.83 (0.95–3.53) 1 study	1.17 (0.64–2.14) 1 study	1.53 (1.17–1.99) 12 studies 97.4%	1.48 (1.16–1.87) 5 studies 78.2%	0.88 (0.34–2.32) 1 study	1.20 (0.89–1.61) 4 studies 58.8%
	*GRADE*	*Very low*	*Very low*		*Very low*	*Very low*	*Very low*	*Very low*	*Very low*	*Very low*	*Very low*	*Very low*
	WLHIV with INSTI-ART vs HIV-negative women	1.17 (1.06–1.30) 2 studies 0.0%	1.09 (0.86–1.39) 1 study						1.20 (1.08–1.33) 1 study	1.15 (0.95–1.39) 1 study		0.89 (0.58–1.36) 1 study
	*GRADE*	*Very low*	*Very low*						*Very low*	*Very low*		*Very low*
**Comparisons of WLHIV receiving different ART classes**												
Cowdell et al., eClinicalMedicine, 2022^46^	WLHIV with PI-ART vs WLHIV with non-PI ART	1.09 (0.95–1.24) 20 studies 68.3%	1.30 (0.78–2.18) 4 studies 43.0%	1.91 (0.61–5.99) 3 studies 95.7%	1.04 (0.85–1.27) 10 studies 63.9%	0.72 (0.37–1.43) 4 studies 37.9%	0.94 (0.03–3.02) 1 study		1.24 (1.08–1.43) 11 studies 66.7%	1.40 (1.09–1.81) 3 studies 0.0%	1.04 (0.60–1.79) 1 study	1.82 (0.97–3.40) 1 study
	*GRADE*	*Very low*	*Very low*	*Very low*	*Very low*	*Very low*	*Very low*		*Very low*	*Very low*	*Very low*	*Very low*
Beck et al., Frontiers in Medicine, 2024^44^	WLHIV with PI-ART vs WLHIV with NNRTI-ART	1.08 (0.93–1.25) 18 studies 71.2%	1.22 (0.76–1.97) 4 studies 30.0%	1.70 (0.45–6.35) 3 studies 96.1%	1.05 (0.84–1.31) 11 studies 58.2%	0.71 (0.35–1.43) 4 studies 68.5%			1.28 (1.09–1.51) 11 studies 62.2%	1.41 (1.08–1.83) 2 studies 0.0%	1.04 (0.60–1.79) 1 study	1.82 (0.97–3.40) 1 study
	*GRADE*	*Very low*	*Very low*	*Very low*	*Very low*	*Very low*			*Very low*	*Very low*	*Very low*	*Very low*
	WLHIV with PI-ART vs WLHIV with INSTI-ART	1.01 (0.83–1.22) 5 studies 4.0%	0.85 (0.36–2.01) 2 studies 0.0%	2.66 (0.39–18.18) 1 study	1.42 (0.82–2.48) 4 studies 50.5%	0.58 (0.26–1.31) 2 studies 0.0%			1.29 (0.71–2.34) 3 studies 47.8%	1.93 (0.27–13.66) 1 study		
	*GRADE*	*Very low*	*Very low*	*Very low*	*Very low*	*Very low*			*Very low*	*Very low*		
	WLHIV PI-ART vs WLHIV with NRTI-ART	1.17 (0.82–1.69) 4 studies 22.2%		1.54 (0.89–2.67) 1 study					0.98 (0.66–1.46) 2 studies 6.2%	0.98 (0.36–2.68) 1 study		
	*GRADE*	*Very low*		*Very low*					*Very low*	*Very low*		
	WLHIV with NNRTI-ART vs WLHIV with NRTI-ART	1.01 (0.76–1.36) 5 studies 0.0%	0.91 (0.17–4.90) 1 study	1.58 (0.79–3.15) 1 study	1.13 (0.70–1.83) 1 study	1.36 (0.31–6.00) 1 study			0.87 (0.43–1.78) 2 studies 26.9%	0.35 (0.07–1.76) 1 study		
	*GRADE*	*Very low*	*Very low*	*Very low*	*Very low*	*Very low*			*Very low*	*Very low*		
	WLHIV with INSTI-ART vs WLHIV with NNRTI-ART	0.98 (0.90–1.06) 6 studies 0.0%	1.03 (0.74–1.42) 4 studies 41.7%	0.36 (0.05–2.60) 1 study	0.80 (0.39–1.64) 4 studies 62.9%	2.88 (0.19–43.09) 2 studies 68.5%			0.95 (0.87–1.04) 5 studies 0.0%	0.92 (0.80–1.05) 2 studies 0.0%		0.82 (0.56–1.19) 2 studies 0.0%
	*GRADE*	*Very low*	*Very low*	*Very low*	*Very low*	*Very low*			*Low*	*Low*		*Low*

The risk ratio, 95% confidence interval (95% CI), number of studies, and *I*^*2*^ (%) of each perinatal outcome associated with WLHIV receiving different ART classes in meta-analyses of cohort studies are shown. GRADE (Grading of Recommendations Assessment, Development and Evaluation) assessments of certainty of evidence for each comparison and perinatal outcome are included.

A risk ratio >1 indicates an increased risk of a perinatal outcome associated with the first-mentioned comparator group compared to the second-mentioned group. For example, WLHIV receiving PI-based ART are associated with an increased risk of PTB compared to HIV-negative women (RR 1.88, 95% CI 1.55–2.28).

Abbreviations: ART: antiretroviral therapy (triple drug), INSTI: integrase strand transfer inhibitor, LBW: low birthweight, NNRTI: non-nucleoside reverse transcriptase inhibitor, NRTI: nucleoside reverse transcriptase inhibitors, NND: neonatal death, PI: protease inhibitor, PTB: preterm birth, SB: stillbirth, SGA: small for gestational age, sPTB: spontaneous preterm birth, VLBW: very low birthweight, VPTB: very preterm birth, VSGA: very small for gestational age, WLHIV: women living with HIV.

**Table 4:** Perinatal outcomes of WLHIV receiving different ART regimens in randomised controlled trials.

Publication	Comparison	Risk ratio (95% CI)								
		PTB	VPTB	sPTB	LBW	VLBW	SGA	SB	NND	Vertical HIV transmission
Tshivuila-Matala et al., AIDS, 2020^37^^,^^a^	**WLHIV receiving different ART regimens compared to WLHIV receiving placebo**									
	WLHIV with ZDV/3TC/ABC vs WLHIV with placebo	0.94 (0.48–1.85)			1.12 (0.60–2.10)	4.56 (0.21–101.52)				0.50 (0.03–8.33)
	*GRADE*	*Very low*			*Very low*	*Very low*				*Very low*
	WLHIV with ZDV/3TC/EFV vs WLHIV with placebo	1.29 (0.61–2.75)			1.23 (0.65–2.33)					0.05 (0.00–1.76)
	*GRADE*	*Very low*			*Very low*					*Very low*
	WLHIV with TDF/FTC/LPV/r vs WLHIV with placebo	1.36 (0.76–2.43)			1.26 (0.72–2.21)	4.11 (0.46–36.99)				0.12 (0.02–0.96)
	*GRADE*	*Very low*			*Very low*	*Very low*				*Very low*
	WLHIV with ZDV/3TC/LPV/r vs WLHIV with placebo	1.43 (0.85–2.40)			1.43 (0.89–2.32)	1.20 (0.13–10.75)				0.13 (0.03–0.58)
	*GRADE*	*Very low*			*Very low*	*Very low*				*Very low*
	**Comparisons of WLHIV receiving different ART regimens**									
	WLHIV with ZDV/3TC/EFV vs WLHIV with ZDV/3TC/ABC	1.38 (0.69–2.77)	2.12 (0.29–15.39)	1.56 (0.81–3.00)	1.10 (0.62–1.95)					0.09 (0.00–5.45)
	*GRADE*	*Very low*	*Very low*	*Very low*	*Very low*					*Very low*
	WLHIV with TDF/FTC/LPV/r vs WLHIV with ZDV/3TC/ABC	1.45 (0.82–2.59)			1.12 (0.67–1.87)	0.90 (0.06–13.60)				0.24 (0.01–4.64)
	*GRADE*	*Very low*			*Very low*	*Very low*				*Very low*
	WLHIV with ZDV/3TC/LPV/r vs WLHIV with ZDV/3TC/ABC	1.52 (0.99–2.35)	2.10 (0.64–6.88)	1.81 (1.21–2.71)	1.27 (0.85–1.91)	0.26 (0.03–2.34)				0.26 (0.02–2.82)
	*GRADE*	*Very low*	*Very low*	*Very low*	*Very low*	*Very low*				*Very low*
	WLHIV with TDF/FTC/LPV/r vs WLHIV with ZDV/3TC/EFV	1.05 (0.54–2.05)			1.02 (0.61–1.72)					2.66 (0.06–114.26)
	*GRADE*	*Very low*			*Very low*					*Very low*
	WLHIV with ZDV/3TC/LPV/r vs WLHIV with ZDV/3TC/EFV	1.10 (0.64–1.90)	0.99 (0.20–4.83)	1.16 (0.69–1.94)	1.16 (0.77–1.76)					2.88 (0.10–80.20)
	*GRADE*	*Very low*	*Very low*	*Very low*	*Very low*					*Very low*
	WLHIV with ZDV/3TC/LPV/r vs WLHIV with TDF/FTC/LPV/r	1.05 (0.72–1.53)			1.14 (0.83–1.56)	0.29 (0.06–1.45)				1.08 (0.19–6.20)
	*GRADE*	*Very low*			*Very low*	*Very low*				*Very low*
Lockman et al., Lancet, 2021^54^	WLHIV with TAF/FTC/DTG vs WLHIV with TDF/FTC/DTG	0.61 (0.31–1.23)			0.66 (0.24, 1.31)	0.32 (0.01, 7.90)	0.73 (0.48–1.08)	0.72 (0.29–1.75)	0.65 (0.11–3.83)	0.99 (0.06–15.74)
	*GRADE*	*Low*			*Very low*	*Very low*	*Low*	*Very low*	*Very low*	*Very low*
	WLHIV with TDF/FTC/DTG vs WLHIV with TDF/FTC/EFV	0.78 (0.44–1.37)			0.78 (0.44, 1.37)	0.51 (0.05, 5.61)	1.10 (0.75–1.60)	2.72 (0.88–8.42)	0.31 (0.09–1.10)	0.00 (0.00–∞)
	*GRADE*	*Low*			*Low*	*Very low*	*Low*	*Very low*	*Low*	*Very low*
	WLHIV with TAF/FTC/DTG vs WLHIV with TDF/FTC/EFV	0.48 (0.25–0.93)			0.54 (0.28, 1.03)	0.20 (0.01, 4.12)	0.80 (0.53–1.21)	1.95 (0.60–6.39)	0.20 (0.04–0.90)	0.00 (0.00–∞)
	*GRADE*	*Low*			*Low*	*Very low*	*Low*	*Very low*	*Low*	*Very low*
Kintu et al., Lancet HIV, 2020^53^	WLHIV with TDF/(FTC/3TC)/DTG vs WLHIV with TDF/(FTC/3TC)/EFV	1.07 (0.61–1.89)						2.90 (0.31–27.52)		0.00 (0.00–∞)
	*GRADE*	*Very low*						*Very low*		*Very low*
Joao et al., Lancet HIV, 2020^52^	WLHIV with ZDV/3TC/RAL vs WLHIV with ZDV/3TC/EFV	1.17 (0.67–2.04)			1.02 (0.60–1.72)	2.94 (0.12–71.7)				0.16 (0.02–1.33)
	*GRADE*	*Very low*			*Very low*	*Very low*				*Very low*

The risk ratio and 95% confidence interval (95% CI) of each perinatal outcome associated with WLHIV receiving different ART regimens in randomised controlled trials, compared to WLHIV receiving placebo or other ART regimens, are shown. GRADE (Grading of Recommendations Assessment, Development and Evaluation) assessments of certainty of evidence for each comparison and perinatal outcome are included.

A risk ratio >1 indicates an increased risk of a perinatal outcome associated with the first-mentioned comparator group compared to the second-mentioned group. For example, WLHIV receiving ZDV/3TC/LPV/r are associated with an increased risk of sPTB compared with WLHIV receiving ZDV/3TC/ABC (RR 1.81 95% CI 1.21–2.71).

Abbreviations: 3TC: lamivudine, ABC: abacavir, ART: antiretroviral therapy (triple drug), DTG: dolutegravir, EFV: efavirenz, FTC: emtricitabine, LBW: low birthweight, LPV/r: lopinavir/ritonavir, NND: neonatal death, PTB: preterm birth, RAL: raltegravir, SB: stillbirth, SGA: small for gestational age, sPTB: spontaneous preterm birth, TAF: tenofovir alafenamide, TDF: tenofovir disoproxil fumarate, VLBW: very low birthweight, VPTB: very preterm birth, VSGA: very small for gestational age, WLHIV: women living with HIV, ZDV: zidovudine.

a Network meta-analysis of randomised controlled trials.

**Table 5:** Perinatal outcomes of WLHIV receiving different antiretroviral drugs in meta-analyses of cohort studies.

Publication	Comparison	Risk ratio (95% CI)										
		PTB	VPTB	sPTB	LBW	VLBW	Term LBW	Preterm LBW	SGA	VSGA	SB	NND
**Comparisons of NNRTI drugs in ART regimens**												
Hey et al., AIDS, 2025^48^	WLHIV with EFV-ART vs HIV-negative women	1.28 (1.18–1.39) 10 studies 63.3%	1.06 (0.93–1.22) 4 studies 8.7%		1.31 (1.21–1.42) 5 studies 0.0%	1.32 (0.65–2.65) 2 studies 0.0%	1.83 (0.95–3.53) 1 study	1.17 (0.64–2.14) 1 study	1.20 (1.15–1.26) 10 studies 39.5%	1.29 (1.19–1.41) 5 studies 39.5%	0.88 (0.34–2.32) 1 study	1.05 (0.87–1.23) 4 studies 60.4%
	*GRADE*	*Very low*	*Low*		*Low*	*Low*	*Very low*	*Very low*	*Low*	*Low*	*Very low*	*Very low*
	WLHIV with NVP-ART vs HIV-negative women	1.26 (1.04–1.53) 3 studies 85.7%	1.51 (1.26–1.82) 1 study		1.45 (1.30–1.62) 3 studies 80.1%				1.65 (1.55–1.76) 3 studies 88.4%	2.28 (2.01–2.57) 1 study		1.48 (1.08–2.04) 1 study
	*GRADE*	*Very low*	*Very low*		*Very low*				*Very low*	*Very low*		*Very low*
**Comparisons of PI drugs in ART regimens**												
Cowdell et al., eClinicalMedicine, 2022^46^	WLHIV with LPV/r-ART vs WLHIV with ATV/r-ART	0.98 (0.75–1.27) 7 studies 33.0%	1.04 (0.42–2.59) 1 study		1.15 (0.95–1.38) 4 studies 0.0%	0.76 (0.24–2.42) 3 studies 49.1%			0.95 (0.53–1.72) 1 study	1.35 (0.79–2.31) 2 studies 0.0%	1.87 (0.82–4.23) 1 study	
	*GRADE*	*Very low*	*Very low*		*Very low*	*Very low*			*Very low*	*Very low*	*Very low*	
	WLHIV with ATV/r-ART vs WLHIV with DRV/r-ART	0.92 (0.55–1.55) 1 study	0.72 (0.15–3.38) 1 study		1.21 (0.69–2.11) 1 study	0.62 (0.17–2.24) 1 study			1.06 (0.52–2.17) 1 study	0.70 (0.35–1.43) 1 study	0.60 (0.31–1.13) 2 studies 0.0%	
	*GRADE*	*Very low*	*Very low*		*Very low*	*Very low*			*Very low*	*Very low*	*Very low*	
	WLHIV with LPV/r-ART vs WLHIV with DRV/r-ART									0.99 (0.52–1.86) 1 study	1.53 (0.47–4.98) 1 study	
	*GRADE*									*Very low*	*Very low*	
	WLHIV with LPV/r-ART vs WLHIV with NFV-ART	1.33 (1.03–1.72) 4 studies 0.0%			1.36 (0.91–2.02) 1 study					1.09 (0.35–3.47) 1 study		
	*GRADE*	*Very low*			*Very low*					*Very low*		
	WLHIV with ATV/r-ART vs WLHIV with NFV-ART	1.63 (0.91–2.92) 4 studies 22.9%			1.06 (0.44–2.52) 1 study					1.09 (0.22–5.37) 1 study		
	*GRADE*	*Very low*			*Very low*					*Very low*		
	WLHIV with boosted PI-ART vs WLHIV with non-boosted PI-ART	1.36 (1.12–1.65) 5 studies 0.0%	1.85 (1.02–3.37) 2 studies 0.0%	1.31 (0.87–1.97) 2 studies 0.0%	1.34 (0.90, 1.99) 1 study					1.09 (0.35–3.47) 1 study		
	*GRADE*	*Very low*	*Very low*	*Very low*	*Very low*					*Very low*		
**Comparisons of NRTI drugs in ART regimens**												
Cowdell et al., Clinical Microbiology and Infection, 2025^47^	WLHIV with TDF-ART vs WLHIV with non-TDF-ART	0.89 (0.81–0.97) 15 studies 56.6%	0.58 (0.40–0.86) 4 studies 69.2%		0.95 (0.87–1.04) 9 studies 0.0%	0.69 (0.41–1.18) 2 studies 0.0%	1.07 (0.73–1.57) 1 study		0.76 (0.59–0.98) 8 studies 92.6%	0.60 (0.48–0.73) 2 studies 56.7%	0.49 (0.31–0.78) 3 studies 72.8%	0.61 (0.40–0.93) 2 studies 0.0%
	*GRADE*	*Very low*	*Very low*		*Very low*	*Very low*	*Very low*		*Very low*	*Very low*	*Very low*	*Very low*
	WLHIV with ZDV-ART vs WLHIV with non- ZDV-ART	1.07 (0.93–1.23) 8 studies 79.8%	1.59 (1.01–2.49) 3 studies 81.2%		1.03 (0.91–1.16) 4 studies 0.0%	1.14 (0.54–2.41) 1 study	0.95 (0.69–1.31) 1 study		1.33 (1.03–1.70) 4 studies 92.1%	1.63 (1.25–2.13) 2 studies 74.3%	2.23 (1.10–4.55) 2 studies 80.3%	1.65 (1.08–2.52) 2 studies 0.0%
	*GRADE*	*Very low*	*Very low*		*Very low*	*Very low*	*Very low*		*Very low*	*Very low*	*Very low*	*Very low*
	WLHIV with ABC-ART vs WLHIV with non-ABC-ART	0.96 (0.87–1.07) 5 studies 0.0%	1.14 (0.65–2.00) 3 studies 59.9%		1.01 (0.91–1.11) 3 studies 0.0%	1.09 (0.45–2.66) 2 studies 77.2%	1.13 (0.97–1.32) 2 studies 0.0%		0.87 (0.71–1.08) 2 studies 0.0%		0.65 (0.26–1.61) 1 study	
	*GRADE*	*Very low*	*Very low*		*Very low*	*Very low*	*Very low*		*Very low*		*Very low*	
	WLHIV with ZDV + XTC-ART vs WLHIV with TDF + XTC-ART	1.12 (0.97–1.29) 6 studies 69.2%	1.62 (1.04–2.52) 3 studies 77.7%		1.04 (0.89–1.22) 3 studies 0.0%	1.14 (0.54–2.41) 1 study	0.93 (0.63–1.38) 1 study		1.52 (1.28–1.82) 3 studies 78.6%	1.68 (1.36–2.06) 2 studies 56.7%	2.19 (1.03–4.67) 2 studies 81.3%	1.65 (1.08–2.52) 2 studies 0.0%
	*GRADE*	*Very low*	*Very low*		*Very low*	*Very low*	*Very low*		*Very low*	*Very low*	*Very low*	*Very low*
	WLHIV with ABC + XTC-ART vs WLHIV with TDF + XTC-ART	0.95 (0.77–1.18) 4 studies 16.0%	1.61 (0.71–3.64) 2 studies 44.7%		1.03 (0.81–1.32) 2 studies 0.0%	1.83 (0.86–3.93) 1 study	0.63 (0.32–1.25) 1 study		1.03 (0.76–1.40) 2 studies 26.9%		0.79 (0.30–2.07) 1 study	
	*GRADE*	*Very low*	*Very low*		*Very low*	*Very low*	*Very low*		*Very low*		*Very low*	
	WLHIV with ABC + XTC-ART vs WLHIV with ZDV + XTC-ART	0.91 (0.74–1.10) 2 studies 0.0%	0.95 (0.47–1.94) 1 study		0.93 (0.68–1.26) 1 study		1.02 (0.63–1.64) 1 study		0.84 (0.67–1.07) 1 study		0.54 (0.21–1.39) 1 study	
	*GRADE*	*Very low*	*Very low*		*Very low*		*Very low*		*Very low*		*Very low*	
	WLHIV with ZDV + XTC + NVP vs WLHIV with TDF + XTC + NVP	1.30 (1.09–1.54) 1 study	1.14 (0.79, 1.66) 1 study						1.13 (0.98, 1.32) 1 study	1.15 (0.90–1.47) 1 study	0.42 (0.11, 1.68) 1 study	1.20 (0.62, 2.30) 1 study
	*GRADE*	*Very low*	*Very low*						*Very low*	*Very low*	*Very low*	*Very low*
	WLHIV with TAF-ART vs WLHIV with TDF-ART				0.69 (0.16–2.90) 1 study							
	*GRADE*				*Very low*							
**Comparisons of drugs of different ART classes**												
Beck et al., Frontiers in Medicine, 2024^44^	**PI-ART compared to NNRTI-ART**											
	WLHIV with LPV/r-ART vs WLHIV with NVP-ART	1.01 (0.69–1.47) 5 studies 87.4%	1.26 (0.85–1.87) 2 studies 0.0%		1.01 (0.78–1.31) 3 studies 29.6%	1.22 (0.02–63.68) 2 studies 78.9%			1.50 (0.55–4.12) 2 studies 96.3%	1.06 (0.81–1.40) 1 study	1.11 (0.58–2.12) 1 study	1.43 (0.74–2.76) 1 study
	*GRADE*	*Very low*	*Very low*		*Very low*	*Very low*			*Very low*	*Very low*	*Very low*	*Very low*
	WLHIV with ATV/r-ART vs WLHIV with NVP-ART	0.81 (0.35–1.86) 1 study			0.88 (0.44–1.77) 2 studies 0.0%						0.60 (0.24–1.49) 1 study	
	*GRADE*	*Very low*			*Very low*						*Very low*	
	WLHIV with NFV-ART vs WLHIV with NVP-ART	0.87 (0.56–1.35) 1 study			0.90 (0.55–1.46) 1 study							
	*GRADE*	*Very low*			*Very low*							
	WLHIV with LPV/r-ART vs WLHIV with EFV-ART	0.64 (0.18–2.36) 2 studies 95.7%	1.66 (1.10–2.50) 1 study		0.48 (0.34–0.66) 1 study	0.64 (0.17–2.44) 1 study			1.40 (1.18–1.65) 1 study	1.84 (1.37–2.45) 1 study	1.49 (0.62–3.56) 1 study	2.42 (1.22–4.80) 1 study
	*GRADE*	*Very low*	*Very low*		*Very low*	*Very low*			*Very low*	*Very low*	*Very low*	*Very low*
	WLHIV with ATV/r-ART vs WLHIV with EFV-ART				0.84 (0.26–2.75) 1 study						0.80 (0.27–2.36) 1 study	
	*GRADE*				*Very low*						*Very low*	
	WLHIV with DRV/r-ART vs WLHIV with EFV-ART										0.98 (0.25–3.88) 1 study	
	*GRADE*										*Very low*	
	**PI-ART compared to INSTI-ART**											
	WLHIV with ATV/r-ART vs WLHIV with DTG-ART	0.88 (0.56–1.39) 1 study	0.45 (0.13–1.52) 1 study		0.97 (0.62–1.52) 1 study	0.36 (0.12–1.12) 1 study			1.00 (0.59–1.70) 1 study			
	*GRADE*	*Very low*	*Very low*		*Very low*	*Very low*			*Very low*			
	WLHIV with DRV/r-ART vs WLHIV with DTG-ART	0.84 (0.49–1.44) 1 study	0.65 (0.17–2.54) 1 study		0.88 (0.52–1.49) 1 study	0.26 (0.05–1.32) 1 study			0.86 (0.46–1.62) 1 study			
	*GRADE*	*Very low*	*Very low*		*Very low*	*Very low*			*Very low*			
	WLHIV with LPV/r-ART vs WLHIV with RAL-ART									2.15 (0.30–15.39) 1 study		
	*GRADE*									*Very low*		
	WLHIV with ATV/r-ART vs WLHIV with RAL-ART	0.97 (0.56–1.68) 1 study	2.81 (0.61–48.69) 1 study		0.99 (0.59–1.67) 1 study	2.81 (0.16–48.69) 1 study			1.08 (0.57–2.02) 1 study	1.52 (0.21–11.19) 1 study		
	*GRADE*	*Very low*	*Very low*		*Very low*	*Very low*			*Very low*	*Very low*		
	WLHIV with DRV/r-ART vs WLHIV with RAL-ART	0.93 (0.50–1.72) 1 study	4.21 (0.23–77.32) 1 study		0.90 (0.50–1.62) 1 study	2.34 (0.11–48.20) 1 study			0.93 (0.45–1.90) 1 study	2.17 (0.29–16.18) 1 study		
	*GRADE*	*Very low*	*Very low*		*Very low*	*Very low*			*Very low*	*Very low*		
	WLHIV with ATV/r-ART vs WLHIV with EVG/c-ART	0.83 (0.56–1.24) 1 study	0.60 (0.18–2.02) 1 study		1.17 (0.75–1.81) 1 study	0.48 (0.15–1.49) 1 study			1.17 (0.70–1.95) 1 study			
	*GRADE*	*Very low*	*Very low*		*Very low*	*Very low*			*Very low*			
	WLHIV with DRV/r-ART vs WLHIV with EVG/c-ART	0.80 (0.49–1.30) 1 study	0.86 (0.22–3.38) 1 study		1.05 (0.63–1.78) 1 study	0.34 (0.07–1.75) 1 study			1.01 (0.55–1.86) 1 study			
	*GRADE*	*Very low*	*Very low*		*Very low*	*Very low*			*Very low*			
	**NNRTI-ART compared to NRTI-ART**											
	WLHIV with NVP-ART vs WLHIV with ABC-ART	1.43 (0.10–21.18) 1 study										
	*GRADE*	*Very low*										
	**INSTI-ART compared to NNRTI-ART**											
	WLHIV with DTG-ART vs WLHIV with EFV-ART	0.97 (0.89–1.06) 2 studies 0.0%	0.97 (0.80–1.19) 2 studies 10.7%						0.94 (0.86–1.03) 2 studies 0.0%	0.92 (0.80–1.05) 2 studies 0.0%		0.92 (0.56–1.51) 1 study
	*GRADE*	*Low*	*Very low*						*Low*	*Very low*		*Very low*
	WLHIV with RAL-ART vs WLHIV with EFV-ART	0.87 (0.35–2.16) 1 study			0.50 (0.19–1.27) 1 study				1.70 (0.76–3.81) 1 study			
	*GRADE*	*Very low*			*Very low*				*Very low*			
	WLHIV with DTG-ART vs WLHIV with RPV-ART	1.31 (0.78–2.19) 1 study	9.08 (1.03–80.32) 1 study		1.33 (0.77–2.32) 1 study	11.36 (1.34–96.03) 1 study			1.45 (0.77–2.71) 1 study			
	*GRADE*	*Very low*	*Very low*		*Very low*	*Very low*			*Very low*			

The risk ratio, 95% confidence interval (95% CI), number of studies, and *I*^*2*^ (%) of each perinatal outcome associated with WLHIV receiving different ART drugs in meta-analyses of cohort studies are shown. GRADE (Grading of Recommendations Assessment, Development and Evaluation) assessments of certainty of evidence for each comparison and perinatal outcome are included.

A risk ratio >1 indicates an increased risk of a perinatal outcome associated with the first-mentioned comparator group compared to the second-mentioned group. For example, WLHIV receiving EFV-based ART are associated with an increased risk of PTB compared to HIV-negative women (RR 1.28, 95% CI 1.18–1.39).

Abbreviations: 3TC: lamivudine, ABC: abacavir, ART: antiretroviral therapy (triple drug), ATV/r: atazanavir/ritonavir, DRV/r: darunavir/ritonavir, DTG: dolutegravir, EFV: efavirenz, EVG/c: elvitegravir/cobicistat, FTC: emtricitabine, INSTI: integrase strand transfer inhibitor, LBW: low birthweight, LPV/r: lopinavir/ritonavir, NFV: nelfinavir, NND: neonatal death, NNRTI: non-nucleoside reverse transcriptase inhibitor, NRTI: nucleoside reverse transcriptase inhibitors, NVP: nevirapine, PI: protease inhibitor, PTB: preterm birth, RAL: raltegravir, RPV: rilpivirine, SB: stillbirth, SGA: small for gestational age, sPTB: spontaneous preterm birth, TAF: tenofovir alafenamide, TDF: tenofovir disoproxil fumarate, VLBW: very low birthweight, VPTB: very preterm birth, VSGA: very small for gestational age, WLHIV: women living with HIV, XTC: either FTC (emtricitabine) or 3TC (lamivudine), ZDV: zidovudine.

**Table 6:** Perinatal outcomes associated with timing of ART initiation in meta-analyses of cohort studies.

Publication	Comparison	Risk ratio (95% CI)								
		PTB	VPTB	sPTB	LBW	VLBW	SGA	VSGA	SB	NND
Boering et al., AIDS, 2025^45^	WLHIV with preconception ART initiation vs HIV-negative women	1.55 (1.27–1.90) 13 studies 92.3%	2.14 (1.02–4.47) 5 studies 89.6%	0.92 (0.69–1.22) 1 study	2.19 (1.32–3.63) 5 studies 90.3%	3.34 (1.08–10.35) 3 studies 70.9%	1.92 (1.01–3.66) 8 studies 99.3%	2.79 (1.04–7.47) 2 studies 84.7%	0.42 (0.03–6.88) 1 study	1.58 (0.66–3.79) 2 studies 21.2%
	*GRADE*	*Very low*	*Very low*	*Very low*	*Very low*	*Very low*	*Very low*	*Very low*	*Very low*	*Very low*
	WLHIV with antenatal ART initiation vs HIV-negative women	1.35 (1.15–1.58) 17 studies 88.5%	1.50 (0.79–2.83) 5 studies 82.8%	1.08 (0.83–1.41) 1 study	2.16 (1.39–3.34) 8 studies 94.7%	1.97 (1.01–3.84) 3 studies 17.2%	1.77 (1.10–2.84) 11 studies 98.2%	1.21 (1.09–1.33) 3 studies 0.0%	1.13 (0.15–8.45) 1 study	1.80 (0.33–9.68) 3 studies 67.2%
	*GRADE*	*Very low*	*Very low*	*Very low*	*Very low*	*Very low*	*Very low*	*Low*	*Very low*	*Very low*
	WLHIV with preconception ART initiation vs WLHIV naïve to ART	1.12 (0.82–1.53) 7 studies 64.7%			1.35 (0.92–2.00) 4 studies 64.9%		1.40 (1.12–1.73) 3 studies 0.0%	2.44 (1.63–3.66) 1 study		
	*GRADE*	*Very low*			*Very low*		*Low*	*Very low*		
	WLHIV with antenatal ART initiation vs WLHIV naïve to ART	0.82 (0.56–1.21) 13 studies 91.9%			1.33 (0.88–2.03) 7 studies 81.7%		1.39 (1.11–1.74) 6 studies 26.5%	2.24 (1.48–3.40) 1 study		1.23 (0.33–4.53) 1 study
	*GRADE*	*Very low*			*Very low*		*Low*	*Very low*		*Very low*
Sexton et al., HIV Medicine, 2023^50^	WLHIV with preconception ART initiation vs WLHIV with antenatal ART initiation	1.16 (1.03–1.31) 18 studies 81.4%	1.16 (0.90–1.49) 6 studies 44.3%		1.08 (0.91–1.28) 12 studies 59.0%	1.12 (0.76–1.66) 4 studies 22.6%	1.04 (0.83–1.30) 14 studies 88.8%	0.89 (0.60–1.32) 3 studies 50.8%		0.93 (0.59–1.47) 2 studies 0.0%
	*GRADE*	*Very low*	*Low*		*Very low*	*Low*	*Very low*	*Very low*		*Low*
Uthman et al., Lancet HIV, 2017^51^	WLHIV with preconception ART initiation vs WLHIV with antenatal ART initiation	1.20 (1.01–1.44) 2 studies 77.0%			1.30 (1.04–1.62) 2 studies 15.6%	0.18 (0.02–1.51) 1 study	1.13 (0.94–1.35) 2 studies 49.%	1.09 (0.82–1.45) 1 study		
	*GRADE*	*Very low*			*Very low*	*Very low*	*Very low*	*Very low*		

The risk ratio, 95% confidence interval (95% CI), number of studies, and *I*^*2*^ (%) of each pregnancy outcome associated with WLHIV depending on the timing of ART initiation in meta-analyses of cohort studies are shown. GRADE (Grading of Recommendations Assessment, Development and Evaluation) assessments of certainty of evidence for each comparison and perinatal outcome are included.

A risk ratio >1 indicates an increased risk of a perinatal outcome associated with the first-mentioned comparator group compared to the second-mentioned group. For example, WLHIV who initiated ART preconception are associated with an increased risk of PTB compared to HIV negative women (RR 1.55, 95% CI 1.27–1.90).

Abbreviations: ART: antiretroviral therapy (triple drug), LBW: low birthweight, NND: neonatal death, PTB: preterm birth, SB: stillbirth, SGA: small for gestational age, sPTB: spontaneous preterm birth, VLBW: very low birthweight, VPTB: very preterm birth, VSGA: very small for gestational age, WLHIV: women living with HIV.

### Ethics

Due to the nature of this study, being based on publicly available published data, ethical approval was not needed.

### Role of the funding source

This study received no funding. The corresponding author had full access to all the data in the study and had final responsibility for the decision to submit for publication.

## Results

Our literature search for meta-analyses yielded 6123 citations and the search for RCTs yielded 8156 citations. Nine meta-analyses of cohort studies, one network analyses of seven RCTs, and three RCTs met the eligibility criteria (Fig. 1).

The characteristics of the included meta-analyses^9^^,^^44^, ^45^, ^46^, ^47^, ^48^, ^49^, ^50^, ^51^ and RCTs^37^^,^^54^, ^53^, ^52^ are shown in Table 1. The meta-analyses included in this review were composed of 154 individual cohort studies, comprising 657,711 women. 97 (63.0%) cohort studies were included in a single meta-analysis publication, whereas data from the remaining 57 (37.0%) studies were used in more than one meta-analysis, with varying exposure comparators and outcomes analysed in each meta-analysis. The overall corrected covered area was 8.9% (Appendix pp 58–61). The corrected covered areas between study pairs are presented in Appendix p 62. A few included study pairs have high corrected covered areas, however they were retained in the umbrella review because different exposure comparisons were analysed in each study. Eighty (51.9%) cohort studies including 503,243 (76.7%) women were conducted in low- and middle-income countries (LMICs) and 73 (47.4%) cohort studies including 138,784 (21.1%) women, were conducted in high-income countries (HICs). One study was conducted in both a HIC and LMIC.^47^^,^^55^ Data on vertical HIV transmission was available from included RCTs, but vertical HIV transmission was not assessed in any of the included meta-analyses of cohort studies. In total, this review includes evidence for 66 exposure comparisons and 359 perinatal outcome results.

The AMSTAR2 tool was used to assess the quality of included meta-analyses of cohort studies and indicated that eight studies (89.0%) were of low quality and one study was of critically low quality (Table 1, Appendix p 12). The quality of the meta-analyses was good in most domains but limited by a lack of lists of excluded studies, which is deemed a critical domain (Appendix p 12). All RCTs were found to be at high risk of bias using RoB1, reflecting the lack of blinding and inaccurate measurement of outcomes (Appendix p 8). GRADE assessments were conducted for all analysis results, of which 29 (7.8%) were of low certainty and 345 (92.2%) were very low certainty (Table 2, Table 3, Table 4, Table 5, Table 6, Appendix pp 17–40). Most GRADE assessments (250, 66.8%) were downgraded due to imprecision of results, of which 151 (60.4%) analyses were composed of a single study. Furthermore, 157 (42.0%) analyses were downgraded due to a lack of directness and 150 (40.1%) due to a high risk of bias. Publication bias, as determined using the Peter's test, was found for two analyses. Heterogeneity was found to be high in 83 (22.2%) analyses (Appendix pp 17–40).

A meta-analysis of 35 cohort studies found that WLHIV naïve to ART are associated with higher risks of PTB (RR 1.63, 95% CI 1.37–1.93, *I*^*2*^ 83.1%), LBW (RR 1.75, 95% CI 1.52–2.02, *I*^*2*^ 76.7%), SGA (RR 1.64, 95% CI 1.29–2.09, *I*^*2*^ 74.9%) and SB (RR 1.67, 95% CI 1.05–2.66, *I*^*2*^ 0.0%), compared to HIV-negative women (Table 2).^9^ Another meta-analysis of 61 cohort studies found that WLHIV receiving ART are associated with increased risks of PTB (RR 1.55, 95% CI 1.38–1.74, *I*^*2*^ 87.4%), sPTB (RR 2.10, 95% CI 1.48–2.96, *I*^*2*^ 12.5%), LBW (RR 1.79, 95% CI 1.51–2.13, *I*^*2*^ 90.6%), term LBW (RR 1.88, 95% CI 1.23–2.85, *I*^*2*^ 0.0%), SGA (RR 1.80, 95% CI 1.34–2.40, *I*^*2*^ 97.6%), and VSGA (RR 1.22, 95% CI 1.10–1.34, *I*^*2*^ 0.0%), compared to HIV-negative women.^49^ WLHIV receiving ART were associated with a lower risk of sPTB (RR 0.46, 95% CI 0.32–0.67, *I*^*2*^ 0.0%) and VLBW (RR 0.36, 95% CI 0.16–0.78, 1 study), but a greater risk of SGA (RR 1.38, 95% CI 1.09–1.75, *I*^*2*^ 38.5%) and VSGA (RR 2.35, 95% CI 1.60–3.46, 1 study), compared to WLHIV naïve to ART (Table 2).^49^

When compared to HIV-negative women, WLHIV receiving PI-, NNRTI- and INSTI-based ART were associated with higher risks of adverse outcomes, including PTB, LBW and SGA (Table 3).^48^ However, there were limited differences in the risk of adverse pregnancy outcomes in the direct comparisons between WLHIV receiving different classes of ART, except for WLHIV receiving PI-based ART, who were associated with a higher risk of SGA and VSGA compared to both non-PI-based ART (SGA: RR 1.24, 95% CI 1.08–1.43, *I*^*2*^ 66.7%; VSGA: RR 1.40, 95% CI 1.09–1.81, *I*^*2*^ 0.0%)^46^ and NNRTI-based ART (SGA: RR 1.28, 95% CI 1.09–1.51, *I*^*2*^ 62.2%; VSGA: RR 1.41, 95% CI 1.08–1.83, *I*^*2*^ 0.0%) specifically (Table 3).^44^

The risks of adverse perinatal outcomes associated with ART regimens compared in RCTs are presented in Table 4. No significant differences in the risks of adverse pregnancy outcomes were found in the vast majority of comparisons of ART regimens. WLHIV receiving ZDV/3TC/LPV/r were associated with a higher risk of sPTB compared with WLHIV receiving ZDV/3TC/ABC (RR 1.81, 95% CI 1.21–2.71) in a network meta-analysis of RCTs.^37^ One RCT found a significantly lower risk of PTB (RR 0.48, 95% CI 0.25–0.93) and NND (RR 0.20, 95% CI 0.04–0.90) associated with WLHIV receiving TAF/FTC/DTG compared to WLHIV receiving TDF/FTC/EFV (Table 4).^54^ Women receiving either TDF/FTC/LPV/r (RR 0.12, 95% CI 0.02–0.96) or ZDV/3TC/LPV/r (RR 0.13, 95% CI 0.03–0.58) were associated with a reduced risk of vertical HIV transmission compared to WLHIV receiving placebo, however no other significant differences in vertical HIV transmission were identified (Table 4).^37^

The risks of adverse perinatal outcomes associated with different ART drugs in meta-analyses of cohort studies are shown in Table 5. When comparing different PI drugs, WLHIV receiving boosted PI-based ART were associated with a higher risk of PTB (RR 1.36, 95% CI 1.12–1.65, *I*^*2*^ 0.0%) and VPTB (RR 1.85, 95% CI 1.02–3.37, *I*^*2*^ 0.0%) compared to WLHIV receiving non-boosted PI-based ART. There were no significant differences in adverse perinatal outcomes in the comparisons of WLHIV receiving LPV/r, DRV/r and ATV/r.^46^ WLHIV receiving TDF-based ART were associated with a significantly lower risk of PTB (RR 0.89, 95% CI 0.81–0.97, *I*^*2*^ 56.6%), VPTB (RR 0.58, 95% CI 0.40–0.86, *I*^*2*^ 69.2%), SGA (RR 0.76, 95% CI 0.59–0.98, *I*^*2*^ 92.6%), VSGA (RR 0.60, 95% CI 0.48–0.73, *I*^*2*^ 56.7%), SB (RR 0.49, 95% CI 0.31–0.78, *I*^*2*^ 72.8%) and NND (RR 0.61, 95% CI 0.40–0.93, *I*^*2*^ 0.0%) compared to WLHIV receiving non-TDF-based ART. Conversely, WLHIV receiving ZDV-based ART (VPTB: RR 1.59, 95% CI 1.01–2.49, *I*^*2*^ 81.2%; SGA: RR 1.33, 95% CI 1.03–1.70, *I*^*2*^ 92.1%; VSGA: RR 1.63, 95% CI 1.25–2.13, *I*^*2*^ 74.3%; SB: RR 2.23, 95% CI 1.10–4.55, *I*^*2*^ 80.3%; NND: RR 1.65, 95% CI 1.08–2.52, *I*^*2*^ 0.0%), as well as WLHIV receiving ZDV+3TC/FTC-based ART (VPTB: RR 1.62, 95% CI 1.04–2.52, *I*^*2*^ 77.7%; SGA: RR 1.52, 95% CI 1.28–1.82, *I*^*2*^ 78.6%; VSGA: RR 1.68, 95% CI 1.36–2.06, *I*^*2*^ 56.7%; SB: RR 2.19, 95% CI 1.03–4.67, *I*^*2*^ 81.3%; NND: RR 1.65, 95% CI 1.08–2.52, *I*^*2*^ 0.0%), were associated with a higher risk of VPTB, SGA, VSGA, SB and NND, when compared to WLHIV receiving non-ZDV-based ART or TDF+ 3TC/FTC-based ART, respectively (Table 5).^47^

There was limited evidence for significant differences in the comparisons of specific drugs from different ART classes in meta-analyses of cohort studies (Table 5). When comparing PI-based ART with NNRTI-based ART, WLHIV receiving LPV/r-based ART were associated with a higher risk of VPTB (RR 1.66, 95% CI 1.10–2.50, 1 study), SGA (RR 1.40, 95% CI 1.18–1.65, 1 study), VSGA (RR 1.84, 95% CI 1.37–2.45, 1 study) and NND (RR 2.42, 95% CI 1.22–4.80, 1 study), but a lower risk of LBW (RR 0.48, 95% CI 0.34–0.66, 1 study), compared with WLHIV receiving EFV-based ART. There were no other significant differences found in the comparison of individual drugs of PI-based ART and NNRTI-based ART, NNRTI-based ART and NRTI-based ART, as well as PI-based ART and INSTI-based ART. In the comparison of INSTI-based ART and NNRTI-based ART, WLHIV receiving DTG-based ART were associated with a higher risk of VPTB and VLBW, compared with WLHIV receiving RPV-based ART, although estimates were very imprecise.^44^

Three meta-analyses of cohort studies assessed the risks of adverse perinatal outcomes among WLHIV associated with timing of ART initiation (Table 6).^45^^,^^50^^,^^51^ Both WLHIV with preconception (PTB: RR 1.55, 95% CI 1.27–1.90, *I*^*2*^ 92.3%; VPTB: RR 2.14, 95% CI 1.02–4.47, *I*^*2*^ 89.6%; LBW: RR 2.19, 95% CI 1.32–3.63, *I*^*2*^ 90.3%; VLBW: RR 3.34, 95% CI 1.08–10.35, *I*^*2*^ 70.9%; SGA: RR 1.92, 95% CI 1.01–3.66, *I*^*2*^ 99.3%; VSGA: RR 2.79, 95% CI 1.04–7.47, *I*^*2*^ 84.7%) or antenatal (PTB: RR 1.35, 95% CI 1.15–1.58, *I*^*2*^ 88.5%; LBW: RR 2.16, 95% CI 1.39–3.34, *I*^*2*^ 94.7%; VLBW: RR1.97, 95% CI 1.01–3.84, *I*^*2*^ 17.2%; SGA: RR 1.77, 95% CI 1.10–2.84, *I*^*2*^ 98.2%; VSGA: RR 1.21, 95% CI 1.09–1.33, *I*^*2*^ 0.0%) ART initiation were associated with a higher risk of PTB, LBW, VLBW, SGA and VSGA, compared to HIV-negative women.^45^ Furthermore, both women with preconception (SGA: RR 1.40, 95% CI 1.12–1.73, *I*^*2*^ 0.0%; VSGA: RR 2.44, 95% CI 1.63–3.66, 1 study) and antenatal (SGA: RR 1.39, 95% CI 1.11–1.74, *I*^*2*^ 26.5%; VSGA: RR 2.24, 95% CI 1.48–3.40, 1 study) initiation of ART were associated with a higher risk of SGA and VSGA compared to WLHIV naïve to ART.^45^ Two meta-analyses reported a higher risk of PTB for WLHIV with preconception ART initiation compared to WLHIV with antenatal initiation (Sexton et al.^50^: RR 1.16, 95% CI 1.03–1.31, *I*^*2*^ 81.4%; Uthman et al.^51^: RR 1.20, 95% CI 1.01–1.44, *I*^*2*^ 77.0%). One meta-analysis also found an increased risk of LBW for WLHIV with preconception ART compared to WLHIV who initiated ART antenatally (RR 1.30, 95% CI 1.04–1.62, *I*^*2*^ 15.6%).^51^

## Discussion

We found that WLHIV receiving ART are at increased risk of PTB, sPTB, LBW, term LBW, SGA, and VSGA, compared to HIV-negative women. RCTs comparing ART regimens among WLHIV found few differences in perinatal outcomes assessed. Meta-analyses of cohort studies comparing different ART regimens indicate that PIs are associated with an increased risk of SGA and VSGA, compared to NNRTIs. TDF is associated with a lower risk of adverse perinatal outcomes and ZDV is associated with an increased risk of adverse perinatal outcomes. Importantly, meta-analyses of cohort studies indicate that WLHIV receiving ART, compared to HIV-negative women, remain at increased risk of adverse perinatal outcomes, irrespective of the ART regimen and timing of ART initiation.

International treatment guidelines currently recommend INSTI-based ART regimens as first line in pregnancy. However, alternative ART regimens remain of clinical importance as they are often used as second- and third-line regimens, in cases where first-line regimens are not available or appropriate due to drug resistance or intolerance.^5^, ^6^, ^7^, ^8^ For this reason we conducted a comprehensive review of the perinatal outcomes associated with different ART regimens.

The results presented in this umbrella review broadly support ART recommendations for pregnant WLHIV in current guidelines.^5^, ^6^, ^7^, ^8^ The use of DTG as part of the first-line ART regimen is corroborated by the results of RCTs, as well as the meta-analyses of cohort studies included in this review.^44^^,^^48^^,^^54^^,^^53^ Furthermore, DTG has also been shown to have superior virological efficacy when compared to EFV.^54^ The use of RAL is supported by data from one RCT and limited cohort data. We found no perinatal outcome data for BIC use in pregnancy. PI-based ART, which is mostly recommended as second line treatment, was found to be associated with increased risk of SGA and VSGA when compared to NNRTI-based ART, but not INSTI-based ART.^44^ Specifically, LPV/r-based ART was associated with a higher risk of SGA and VSGA compared with EFV-based ART.^44^ However, this was not demonstrated in the network meta-analysis of RCTs, which may reflect small sample sizes and late ART initiation during pregnancy.^37^ There were no significant differences in adverse perinatal outcomes in the comparisons of WLHIV receiving the PIs LPV/r, DRV/r and ATV/r.^46^ However, while much data (including RCT data and cohort meta-analyses) were available for LPV/r, which is no longer recommended by most guidelines, much less data was available for the preferred PIs DRV/r and ATV/r, for which there was limited cohort data. Use of EFV was supported by RCT and cohort meta-analysis data, but data on RPV use in pregnancy was extremely limited (one small cohort study only), leaving us unable to draw any conclusions. TDF was found to be associated with a lower risk of adverse perinatal outcomes when compared to ZDV in meta-analyses of cohort studies, consistent with ART pregnancy guidelines which recommend tenofovir.^47^ The same association was not seen in the network meta-analysis of RCTs,^37^ which may reflect small sample sizes and differences in study setting, inclusion criteria, and timing of ART initiation. The evidence in this review also supports the use of TDF as pre-exposure prophylaxis during pregnancy.^56^ There was extremely limited cohort data on the use of TAF in pregnancy (one small study comparing TAF to TDF for LBW only), preventing us from drawing any conclusions.

Although RCTs are essential for comparing the safety and efficacy of ART regimens in pregnant WLHIV, we found that few RCTs of ART among pregnant WLHIV have been conducted to date. These RCTs tended to be small and assessed a limited range of ART regimens. Moreover, all RCTs had selective enrolment criteria (high CD4 counts) and initiated ART during pregnancy, often late. We assessed that all RCTs of ART among WLHIV had a high risk of bias due to lack of blinding and challenges related to outcome measurement. Seven RCTs were assessed together in a network meta-analysis, but unfortunately the three most recently conducted RCTs could not be incorporated into this network due to a lack of overlapping ART regimens. Further RCTs are needed to further assess the risk of adverse perinatal outcomes associated with ART, particularly new regimens such as long-acting preparations.^57^ Evidence relating to vertical HIV transmission was included in all RCTs, however studies were often underpowered to identify a statistically significant difference in risk between ART regimens. It is important that future RCTs are sufficiently powered to assess rarer perinatal outcomes and that they are conducted according to best practice.

This study has several strengths. This is the first umbrella review of meta-analyses and RCTs reporting on the association of pregnant WLHIV receiving ART with adverse perinatal outcomes. This study presents 359 analysis results from nine meta-analyses including 154 cohort studies (comprising 657,711 pregnant women), one network meta-analysis of seven RCTs, and three additional RCTs. We made a comprehensive assessment of a broad range of perinatal outcomes associated with WLHIV receiving ART, compared to WLHIV receiving different ART regimens, WLHIV naïve to ART, and women without HIV. We restricted our analysis to WLHIV receiving ART (triple drug therapy), explicitly excluding WLHIV receiving zidovudine monotherapy, as this regimen is no longer recommended. Importantly, the perinatal outcomes assessed were clearly predefined, and studies were not included if outcomes were defined differently or undefined. The study was registered with PROSPERO and results are reported in accordance with the PRISMA and PRIOR guidelines.

This study also has some limitations. As an umbrella review, this study is limited by the available published evidence, which may result in the perpetuation of publication bias. Limited data were available for several exposure comparisons and perinatal outcomes. None of the meta-analyses of cohort studies included in this study assessed or reported evidence regarding vertical HIV transmission, which limits our understanding of the benefit gained from ART during pregnancy and best practice in the prevention of vertical HIV transmission. The results included in this review may be impacted by selection bias which may result in an over-estimated effect size due to systematic differences in duration of exposure as well as time at risk. This may be particularly pertinent when comparing between preconception and antenatal initiation of ART, as well as when comparing WLHIV receiving ART with those who are naive to ART.^58^ Such comparisons may be vulnerable to immortal time bias, where person time at risk is artificially reduced for those who are exposed to ART.^59^ We were unable to directly examine the impact of confounders due to the review methodology. Both the meta-analyses and RCTs included in this study were assessed to be of low or very low quality, limiting the strength of evidence.

The mechanisms underlying the risk of adverse perinatal outcomes associated with maternal HIV infection and the impact of ART are poorly understood. Pregnancy presents a significant shift in maternal immunological functioning, which is necessary for successful completion of the pregnancy.^60^ Severe maternal HIV infection is associated with greater risks of adverse perinatal outcomes.^61^^,^^62^ HIV infection leads to systemic chronic immune activation and the rapid depletion of CD4 cells which may not be entirely reversed by ART.^63^ Furthermore, the depletion of innate lymphoid cells and γδ T cells during acute HIV infection is not reversed by ART, and has been associated with increased risk of preterm birth.^64^^,^^65^ HIV also alters the ratio of CD4 to CD8 cells throughout pregnancy, and lower CD4/CD8 ratios have also been associated with an increased risk of preterm birth.^66^ Exposure to PI-based ART during pregnancy has been associated with decidual and uteroplacental dysfunction, as well as changes in oestradiol and prolactin, which have been correlated with adverse pregnancy outcomes.^23^^,^^67^^,^^68^ Furthermore, PI-based ART, but not NNRTI-based ART, resulted in lower levels of progesterone, which was associated with reduced foetal weight in mouse models, and subsequent progesterone supplementation improved foetal weight.^67^

In the context of pregnancy, immediate initiation of lifelong ART has important benefits including reducing maternal morbidity and mortality, prevention of vertical HIV transmission, reducing horizontal HIV transmission, and protection of future pregnancies. Risks of adverse perinatal outcomes among WLHIV receiving ART are very similar to WLHIV who do not receive ART and ART should not be withheld for this reason. The limited number of RCTs among pregnant WLHIV found few differences in perinatal outcomes between the ART regimens assessed and support the use of DTG-based ART regimens with TDF/TAF and FTC/3TC backbone as first line. Cohort data indicate that PIs are associated with an increased risk of SGA and VSGA, compared to NNRTIs, and that TDF is associated with a lower risk of adverse perinatal outcomes compared to ZDV, also supporting current guidelines.

To strengthen the evidence base for ART guidelines for pregnant WLHIV, there is a clear need for more, larger, well-conducted RCTs and prospective observational studies of perinatal outcomes among pregnant WLHIV receiving different ART regimens. This is particularly important for individual INSTI-based ART drugs and regimens, including DTG, RAL, BIC, and elvitegravir, as well as long-acting injectable cabotegravir/RPV, as INSTI-based ART is currently recommended as first-line. Dual drug regimens should be assessed for safety and efficacy in pregnancy,^69^ as well as novel therapies such as the first-in-class HIV-1 capsid inhibitor lenacapavir, and monoclonal antibodies.^70^ It is essential that studies collect and report detailed information about ART regimens, timing of ART initiation, and perinatal outcomes, and correct for potential confounders. It is also crucial to have long-term follow-up studies to assess the effects of intrauterine ART exposure on the growth and neurodevelopment of HIV-exposed uninfected children.^71^^,^^72^

While it is clear that ART in pregnancy has important benefits for maternal health and reduces vertical and horizontal HIV transmission, pregnant WLHIV receiving ART remain at increased risk of a broad range of adverse perinatal outcomes, compared to women without HIV, irrespective of the ART regimen and timing of ART initiation. Therefore, the burden of adverse perinatal outcomes among WLHIV will continue to remain high, especially in regions with a high HIV prevalence, such as sub-Saharan Africa. It remains vital to obtain further high quality evidence through a range of methodologies, including RCTs and observational studies such as prospective cohort studies, to optimise ART regimens in pregnancy. Novel study designs including the use of routinely collected data and platform trials may be valuable to further elucidate the impact of ART on pregnancy outcomes. Additionally, further studies are needed to determine the mechanisms leading to adverse perinatal outcomes among WLHIV receiving ART to further develop interventions to improve perinatal outcomes in WLHIV.

## Contributors

PB and JH screened the literature search results for relevant manuscripts and assessed their eligibility, verified and extracted data, and conducted methodological quality assessments. PB interpreted the data and wrote the first draft of the manuscript. JH conceived, designed and coordinated the study, developed the systematic review protocol, interpreted the data and wrote the manuscript. All authors had full access to all the data in the study and had final responsibility for the decision to submit for publication.

## Data sharing statement

All data included in this umbrella review are included within the article and its supplementary materials. More detailed data is available from the primary publications included in this study.

## Declaration of interests

JH is a member of the writing group of the British HIV Association (BHIVA) guidelines on the management of HIV in pregnancy and the postpartum period 2025. PB declares no competing interests.
