# Supplementary material for: Perinatal outcomes associated with antiretroviral therapy for pregnant women living with HIV: an umbrella review
Source: eClinicalMedicine. 2026 May 7;95:103919. doi: 10.1016/j.eclinm.2026.103919 (PMC13185840; doi:10.1016/j.eclinm.2026.103919)
Supplement: Appendix [file mmc1.docx]

**Appendix: Perinatal outcomes associated with antiretroviral therapy for women living with HIV: an umbrella review.**

Table of Contents

[Appendix 1: Literature search strategy. 3](#_Toc222234599)

[Appendix 2: Cochrane risk of bias assessment of randomised controlled trials (RoB1) 8](#_Toc222234600)

[Appendix 3: Overall risk of bias of randomised controlled trials (RoB1) 9](#_Toc222234601)

[Appendix 4: A Measurement Tool to Assess systematic Reviews, version 2 (AMSTAR 2) 10](#_Toc222234602)

[Appendix 5: Overall AMSTAR 2 assessment 11](#_Toc222234603)

[Appendix 6: AMSTAR 2 assessment of meta-analyses 12](#_Toc222234604)

[Appendix 7: GRADE assessments for randomised controlled trials 13](#_Toc222234605)

[Appendix 8: GRADE assessments for meta-analyses 14](#_Toc222234606)

[Appendix 9: GRADE overall assessment criteria 15](#_Toc222234607)

[Appendix 10: Risk of preterm birth of WLHIV receiving different PI drugs. 16](#_Toc222234608)

[Appendix 11: Using the Grading of Recommendation, Assessment, Development, and Evaluation (GRADE) approach to assess the certainty of evidence 17](#_Toc222234609)

[Appendix 11.1: GRADE Assessment for Wedi et al, Lancet HIV, 2016. 17](#_Toc222234610)

[Appendix 11.2: GRADE Assessment for Portwood et al, AIDS, 2022 18](#_Toc222234611)

[Appendix 11.3: GRADE Assessment for Hey et al, AIDS, 2025 19](#_Toc222234612)

[Appendix 11.4: GRADE Assessment for Cowdell et al, eClinicalMedicine, 2022 21](#_Toc222234613)

[Appendix 11.5: GRADE Assessment for Cowdell et al, Clinical Microbiology and Infection, 2025 24](#_Toc222234614)

[Appendix 11.6: GRADE Assessment for Beck et al, Frontiers in Medicine, 2024 27](#_Toc222234615)

[PI-ART compared to NNRTI-ART 27](#_Toc222234616)

[PI-ART compared to INSTI-ART 28](#_Toc222234617)

[NNRTI-ART compared to NRTI-ART 30](#_Toc222234618)

[INSTI-ART compared to NNRTI-ART 30](#_Toc222234619)

[Appendix 11.7: GRADE Assessment for Boering et al, AIDS, 2025 32](#_Toc222234620)

[Appendix 11.8: GRADE Assessment for Uthman et al, Lancet HIV, 2017 34](#_Toc222234621)

[Appendix 11.9: GRADE Assessment for Sexton et al, HIV Medicine, 2023 35](#_Toc222234622)

[Appendix 11.10: GRADE Assessment for Joao et al, Lancet HIV 2020 35](#_Toc222234623)

[Appendix 11.11: GRADE Assessment for Kintu et al, Lancet HIV 2020 36](#_Toc222234624)

[Appendix 11.12: GRADE Assessment for Lockman et al, Lancet 2020 36](#_Toc222234625)

[Appendix 11.13: GRADE Assessment for Tshivuila-Matala et al, AIDS, 2020 37](#_Toc222234626)

[WLHIV receiving different ART regimens compared to WLHIV receiving placebo 37](#_Toc222234627)

[Comparisons of WLHIV receiving different ART regimens 38](#_Toc222234628)

[Appendix 12: Excluded studies 41](#_Toc222234629)

[Appendix 12.1 Excluded studies: meta-analysis search 41](#_Toc222234630)

[Appendix 12.2 Excluded studies: randomised controlled trials 54](#_Toc222234631)

[Appendix 13: Corrected covered area 58](#_Toc222234632)

[Appendix 13.1: Overall corrected covered area 58](#_Toc222234633)

[Appendix 13.2 Between-study corrected coverage area 62](#_Toc222234634)

# Appendix 1: Literature search strategy.

1.Pregnancy outcome/ or Pregnancy Complication/ or Fetus outcome/ or Labor complication/ or Perinatal morbidity/

2.((pregnancy or gestation$ or fetal or foetal or obstetric$ or labor or labour or birth or delivery or neonate or newborn or "new-born" or "new born") adj1 (outcome$ or complication$ or consequence$ or characteristic$ or event$ or result$ or problem$ or morbidit$ or sequelae)).ti,ab.

3.((infant or reproductive or prelabour or prelabor or "pre-labour" or "pre-labor" or intrauterine or "intra-uterine" or antenatal or "ante-natal" or prenatal or "pre-natal") adj1 (outcome$ or complication$ or consequence$ or characteristic$ or event$ or result$ or problem$ or morbidit$ or sequelae)).ti,ab.

4.((perinatal or "peri-natal" or neonatal or "neo-natal" or postnatal or "post-natal" or antepartum or "ante-partum" or intrapartum or "intra-partum" or peripartum or "peri-partum" or postpartum or "post-partum") adj1 (outcome$ or complication$ or consequence$ or characteristic$ or event$ or result$ or problem$ or morbidit$ or sequelae)).ti,ab.

5.Prematurity/ or Premature labor/ or Premature fetus membrane rupture/ or "Immature and premature labor"/

6.("premature birth" or prematurity or "gestational age at birth" or "gestational age at delivery" or "pre-terms" or preterms).ti,ab.

7.(PTB or PTBs or VPTB or VPTBs or PTL or PTLs or VPTL or VPTLs or PTD or PTDs or VPTD or VPTDs or PROM or PPROM).ti,ab.

8.(("pre-term" or preterm or premature) adj1 (birth$ or labour$ or labor$ or deliver$ or infant$)).ti,ab.

9.(("pre-term" or preterm or premature) adj3 obstetric adj3 (labor$ or labour$)).ti,ab.

10.(("pre-term" or preterm or premature) adj1 ("rupture of membranes" or "rupture of fetal membranes" or "rupture of foetal membranes")).ti,ab.

11.Intrauterine growth retardation/ or Small for date infant/ or Low birth weight/ or Very low birthweight/ or Extremely low birth weight/

12.(IUGR or FGR or SGA or SFGA or VSGA or SFD or LBW or VLBW or ELBW).ti,ab.

13.((intrauterine or "intra-uterine" or fetal or foetal) adj1 growth adj1 (restriction or restricted or retardation)).ti,ab.

14.("small for gestational age" or "small-for-gestational-age" or "small-for-gestational age" or "small for gestation" or "small-for-gestation" or "very-small-for-gestational-age" or "very-small-for-gestational age" or "small for dates" or "small-for-dates" or "weight for dates" or "weight for gestational age" or "weight for age at delivery" or "weight at delivery" or "birthweight for dates" or "birthweight for gestational age" or "birthweight for age at delivery" or "birth weight for dates" or "birth weight for gestational age" or "birth weight for age at delivery" or "birth-weight for dates" or "birth-weight for gestational age" or "birth-weight for age at delivery").ti,ab.

15.("low BW" or "low birth weight" or "low birth-weight" or "low-birth weight" or "low-birth-weight" or "low birthweight" or "low-birthweight" or "lower BW" or "lower birth weight" or "lower birth-weight" or "lower-birth weight" or "lower-birth-weight" or "lower birthweight" or "lower-birthweight" or "reduced birth weight" or "reduced birthweight" or "reduced birth-weight" or "very-low birthweight" or "very-low birth weight" or "very-low birth-weight" or "very-low-birthweight" or "very-low-birth-weight" or "extremely-low birthweight" or "extremely-low birth weight" or "extremely-low birth-weight" or "extremely-low-birthweight" or "extremely-low-birth-weight").ti,ab.

16.Stillbirth/ or Fetus death/

17.(stillbirth$ or stillborn$ or abortion$ or miscarriage$).ti,ab.

18.(still adj1 (born$ or birth$)).ti,ab.

19.((pregnancy or gestation$ or fetal or foetal or obstetric$ or labour or labor or birth or delivery or neonat$ or newborn or "new-born" or "new born" or infant or reproductive or prelabour or "pre-labour" or prelabor or "pre-labor" or intrauterine or "intra-uterine" or antenatal or "ante-natal" or prenatal or "pre-natal") adj1 (death$ or loss$ or demise$ or mortalit$)).ti,ab.

20.((perinatal or "peri-natal" or "neo-natal" or postnatal or "post-natal" or antepartum or "ante-partum" or intrapartum or "intra-partum" or peripartum or "peri-partum" or postpartum or "post-partum") adj1 (death$ or loss$ or demise$ or mortalit$)).ti,ab.

21.1 or 2 or 3 or 4 or 5 or 6 or 7 or 8 or 9 or 10 or 11 or 12 or 13 or 14 or 15 or 16 or 17 or 18 or 19 or 20

22.Human immunodeficiency virus/ or Human immunodeficiency virus 1/ or Human immunodeficiency virus 1 infection/ or Human immunodeficiency virus 2/ or Human immunodeficiency virus 2 infection/ or Human immunodeficiency virus infection/ or Human immunodeficiency virus infected patient/ or Acquired immune deficiency syndrome/ or Serodiagnosis/ or HIV associated dementia/ or HIV associated lipodystrophy/ or HIV associated nephropathy/ or AIDS related complex/

23.("HIV seropositivity" or "HIV infection$" or "AIDS serodiagnosis" or "AIDS arteritis" or "AIDS-associated nephropathy" or "AIDS dementia complex" or "AIDS-related opportunistic infection$" or "AIDS-related lymphoma").ti,ab.

24.(HIV or AIDS or "HIV-1" or "HIV-type-1" or "HTLV III" or "HTLV type III" or "HTLV-III" or "HTLV-type-III" or LAV or "HTLV-III-LAV" or "LAV-HTLV-III" or "HIV-2" or "HIV-type-2" or "HIV-II" or "HTLV-IV" or "LAV-20" or "HIV-positive" or "HIV-1-positive" or "HIV-2-positive" or "HIV-infected" or "HIV-1-infected" or "HIV-type-1-infected" or "HTLV-III-infected" or "HTLV-type-III-infected" or "LAV-infected" or "HTLV III-infected" or "HTLV type III-infected" or "HTLV-III-LAV-infected" or "LAV-HTLV-III-infected" or "HIV-2-infected" or "HIV-type-2-infected" or "HIV-II-infected" or "HTLV-IV-infected").ti,ab.

25.("LAV-2-infected" or "HIV-infection$" or "HIV-1-infection$" or "HIV-type-1-infection$" or "HTLV-III-infection$" or "HTLV-type-III-infection$" or "HTLV III-infection$" or "HTLV type III-infection$" or "LAV-infection$" or "HTLV-III-LAV-infection$" or "LAV-HTLV-III-infection$" or "HIV-2-infection$" or "HIV-type-2-infection$" or "HIV-II-infection$" or "HTLV-IV-infection$" or "LAV-2-infection$").ti,ab.

26.("Human Immunodeficiency Virus$" or "Human Immune Deficiency Virus$" or "Human T Cell Lymphotropic Virus Type III" or "Human T-Cell Lymphotropic Virus Type III" or "Human T Lymphotropic Virus Type III" or "Human T-Lymphotropic Virus Type III" or "Human T Lymphotropic Virus Type IV" or "Human T-Lymphotropic Virus Type IV" or "Human T Cell Leukemia Virus Type III" or "Human T-Cell Leukemia Virus Type III" or "Lymphadenopathy-Associated Virus$" or "Lymphadenopathy Associated Virus$" or "Acquired Immune Deficiency Syndrome" or "Acquired Immunodeficiency Syndrome").ti,ab.

27.22 or 23 or 24 or 25 or 26

28.Human immunodeficiency virus antibody/ or Human immunodeficiency virus antigen/ or exp Human immunodeficiency virus fusion inhibitor/ or exp Human immunodeficiency virus proteinase inhibitor/ or exp Anti human immunodeficiency virus agent/ or exp Integrase inhibitor/ or exp RNA directed DNA polymerase inhibitor/ or Antiretrovirus agent/ or Proteinase inhibitor/

29. (antiretroviral$ or "anti-retroviral$").ti,ab.

30.(("anti-HIV" or HIV or "anti-HIV-1" or "HIV-1" or "anti-HIV-2" or "HIV-2" or "anti-AIDS" or AIDS or combination or combined) adj1 (treatment$ or therap$ or regimen$ or drug$ or agent$)).ti,ab.

31.(antiviral$ or "anti-viral$").ti,ab.

32.(HAART or ARV or ARVs or cARV or cARVs or "HAART-exposed" or "HAART-treated" or "Mega-HAART" or "ARV-exposed" or "ARV-treated" or "combination-ARV" or "combination-ARVs" or "combined-ARV" or "combined-ARVs" or ART or "Multi-ART" or "Triple-ART" or cART or "ART-exposed" or "ART-treated" or "combination-ART" or "combined-ART" or "sc-ART" or "short-course-antiretroviral therap$" or "short-course-anti-retroviral therap$").ti,ab.

33.(monotherap$ or "mono-therap$" or "dual therap$" or "dual drug therap$" or bitherap$ or PI or PIs or "PI-based" or "PI-boosted" or "PI-containing" or "PI-therap$" or "PI-treatment$" or "PI-regimen$" or "Ritonavir-boosted" or NRTI or NRTIs or "NRTI-based" or "protease inhibitor$" or "NRTI-containing" or "NRTI-therap$" or "NRTI-treatment$" or "NRTI-regimen$" or "nucleoside reverse transcriptase inhibitor$" or "nucleoside analog reverse transcriptase inhibitor$").ti,ab.

34.(NNRTI or NNRTIs or "NNRTI-based" or "NNRTI-containing" or "NNRTI-therap$" or "NNRTI-treatment$" or "NNRTI-regimen$" or "non nucleoside reverse transcriptase inhibitor$" or "non-nucleoside reverse transcriptase inhibitor$" or "nonnucleoside reverse transcriptase inhibitor$" or "non nucleoside analog reverse transcriptase inhibitor$" or "non-nucleoside analog reverse transcriptase inhibitor$" or "nonnucleoside analog reverse transcriptase inhibitor$").ti,ab.

35. (NtRTI or NtRTIs or "NtRTI-based" or "NtRTI-containing" or "NtRTI-therap$" or "NtRTI-treatment$" or "NtRTI-regimen$" or "nucleotide reverse transcriptase inhibitor$" or "nucleotide analog reverse transcriptase inhibitor$" or "fusion inhibitor$" or "CCR5 receptor antagonist$" or "integrase inhibitor$" or "maturation inhibitor$" or "entry inhibitor$").ti,ab.

36.(Abacavir or ABC or Didanosine or ddI or Emtricitabine or FTC or Lamivudine or 3TC or Stavudine or d4T or Tenofovir or TFV or TDF or TAF or Zidovudine or AZT or ZDV or Delavirdine or DLV or Efavirenz or EFV or Etravirine or ETR or Nevirapine or NVP or Rilpivirine or RPV or Atazanavir or ATV or Darunavir or DRV or Fosamprenavir or FPV or Indinavir or IDV or Lopinavir or LPV or Nelfinavir or NFV or Ritonavir or RTV or Saquinavir or SQV or Tipranavir or TPV or Enfuvirtide or "T-20" or Maraviroc or MVC or Raltegravir or RAL or Elvitegravir or EVG or Zalcitabine or "ddC").ti,ab.

37.Abacavir/ or Abacavir plus lamivudine/ or Abacavir plus lamivudine plus zidovudine/ or Didanosine/ or Emtricitabine/ or Efavirenz plus emtricitabine plus tenofovir disoproxil/ or Emtricitabine plus rilpivirine plus tenofovir disoproxil/ or Emtricitabine plus tenofovir disoproxil/

38.Lamivudine/ or Efavirenz plus lamivudine plus zidovudine/ or Lamivudine plus nevirapine plus stavudine/ or Lamivudine plus nevirapine plus tenofovir disoproxil/ or Lamivudine plus nevirapine plus zidovudine/ or Lamivudine plus stavudine/ or Lamivudine plus tenofovir disoproxil/ or Lamivudine plus zidovudine/

39.Stavudine/ or Tenofovir/ or Cobicistat plus elvitegravir plus emtricitabine plus tenofovir disoproxil/ or Tenofovir disoproxil/ or Tenofovir 3 hexadecyloxypropyl ester/ or Tenofovir alafenamide/ or Zidovudine/ or Zidovudine 5' phosphate/ or Zidovudine 5' triphosphate/ or Zidovudine derivative/ or Zidovudine glucuronide/

40.Delavirdine/ or Efavirenz/ or Etravirine/ or Nevirapine/ or Rilpivirine/ or Atazanavir/ or Atazanavir plus ritonavir/

41.("Atazanavir/Ritonavir" or "ATV/r" or "Darunavir/Ritonavir" or "DRV/r" or "Fosamprenavir/Ritonavir" or "FPV/r" or "Indinavir/Ritonavir" or "IDV/r" or "Lopinavir/Ritonavir" or "LPV/r" or "Nelfinavir/Ritonavir" or "NFV/r" or "Saquinavir/Ritonavir" or "SQV/r" or "Tipranavir/Ritonavir" or "TPV/r").ti,ab.

42.Darunavir/ or Darunavir plus ritonavir/ or Fosamprenavir/ or Fosamprenavir plus ritonavir/ or Indinavir/ or Indinavir plus ritonavir/ or Lopinavir/ or Lopinavir plus ritonavir/ or Nelfinavir/ or Ritonavir/ or Saquinavir/ or Ritonavir plus saquinavir/ or Tipranavir/ or Ritonavir plus tipranavir/ or Enfuvirtide/ or Maraviroc/ or Raltegravir/ or Elvitegravir/ or Zalcitabine/

43.Dolutegravir/ or Amprenavir/ or Dolutegravir plus rilpivirine/ or Lamivudine plus raltegravir/ or Abacavir plus dolutegravir plus lamivudine/ or Emtricitabine plus tenofovir alafenamide/ or Emtricitabine plus rilpivirine plus tenofovir alafenamide/ or Atazanavir plus Cobicistat/ or Cobicistat plus Darunavir/ or Emtricitabine plus Tenofovir Disoproxil/ or Dolutegravir plus Rilpivirine/ or Cobicistat plus Elvitegravir plus Emtricitabine plus Tenofovir Alafenamide/ or Cobicistat plus Elvitegravir plus Emtricitabine plus Tenofovir Disoproxil/

44.(Dolutegravir or INSTI or DTG or Tivicay or Isentress or Vitekta or "Integrase strand transfer inhibitor$" or Fuzeon or FPR1 or ENF or Seizentry or Celsentri or "Formyl peptide receptor 1" or Ziagen or Videx or Emtriva or Coviracil or Zerit or Viread or Vemlidy or Retrovir or Azidothymidine or "Diarylpyrimidine analogue" or "Darunavir plus cobicistat" or Juluca or Dutrebis or Stribild or Triumeq or Odefsey or Complera or Descovy or Genvoya or Evotaz or Prezcobix).ti,ab.

45.(Rescriptor or Sustiva or Intelence or TMC125 or DAPY or Viramune or Edurant or TMC278 or Reyataz or APV or Agenerase or Prezista or Lexiva or Telzir or Crixivan or "LPV/r" or "ABT-378" or Kaletra or Norvir or Viracept or "AG1343" or Invirase or Fortovase or Aptivus or "Rilpivirine plus dolutegravir" or "Raltegravir plus lamivudine" or "Abacavir plus lamivudine plus dolutegravir" or "Emtricitabine plus tenofovir alafenamide" or "Emtricitabine plus rilpivirine plus tenofovir alafenamide" or "Atazanavir plus cobicistat").ti,ab.

46.(Combivir or Trizivir or Kaletra or Epzicom or Kivexa or Truvada or Atripla or "RPV/DTG" or "Raltegravir plus lamivudine" or "RAL/3TC" or "Abacavir plus lamivudine plus dolutegravir" or "ABC/3TC/DTG" or "Emtricitabine plus tenofovir alafenamide" or "FTC/TAF" or "Emtricitabine plus rilpivirine plus tenofovir alafenamide" or "FTC/RPV/TAF" or "Atazanavir plus cobicistat" or "ATV/COBI" or "Darunavir plus cobicistat" or "DRV/COBI").ti,ab.

47.Cobicistat/ or Bictegravir/ or Bictegravir plus emtricitabine plus tenofovir alafenamide/ or Lamivudine plus tenofovir disoproxil/ or Doravirine/ or Doravirine plus lamivudine plus tenofovir disoproxil/ or Ibalizumab/ or Tenofovir alafenamide/ or Cobicistat plus darunavir plus emtricitabine plus tenofovir alafenamide/ or Dolutegravir plus lamivudine/ or Doravirine plus lamivudine plus tenofovir disoproxil/ or Efavirenz plus emtricitabine plus tenofovir disoproxil/ or Efavirenz plus lamivudine plus tenofovir disoproxil/ or Atazanavir/

48.(Cobicistat or "cobicistat-boosted" or COBI or Rezolsta or QUAD or Epivir or Temixys or Cimduo or Selzentry or Doravirine or DOR or Pifeltro or "Ibalizumab-uiyk" or Hu5A8 or IBA or Ibalizumab or "TMB-355" or "TNX-355" or Trogarzo or Bictegravir or BIC or "Bictegravir, Emtricitabine, Tenofovir Alafenamide" or "bictegravir sodium/emtricitabine/tenofovir alafenamide fumarate" or "BIC/FTC/TAF" or Biktarvy or "tenofovir alafenamide fumarate" or "darunavir ethanolate, cobicistat, emtricitabine, tenofovir alafenamide fumarate" or "DRV/COBI/FTC/TAF").ti,ab.

49.(Symtuza or "Dolutegravir and lamivudine" or "dolutegravir sodium/lamivudine" or "DTG/3TC" or Dovato or "Doravirine, lamivudine, and tenofovir disoproxil fumarate" or "doravirine/lamivudine/tenofovir disoproxil fumarate" or "DOR/3TC/TDF" or Delstrigo or "Efavirenz, lamivudine, and tenofovir disoproxil fumarate" or "EFV/3TC/TDF" or Symfi or "Symfi Lo" or "Elvitegravir, cobicistat, emtricitabine, and tenofovir alafenamide fumarate" or "elvitegravir/cobicistat/emtricitabine/tenofovir alafenamide fumarate" or "EVG/COBI/FTC/TAF" or Genvoya or "TMC-114" or "TMC114" or Dideoxyinosine or Racivir or Heptovir or Hepitec).ti,ab.

50. (Zerut or Estavudina or Sanilvudine or Apropovir or Stocrin or Zrivada or Aluvia or Aluviran or Pentafuside or Symtuza or "Efavirenz, Lamivudine, Tenofovir Disoproxil Fumarate drug combination" or "Lopinavir-Ritonavir drug combination" or NRTTIs or NRTTI or "nucleoside reverse transcriptase translocation inhibitor" or "islatravir" or ISL or "MK-8591").ti,ab.

51.("Capsid inhibitor$" or "CAI" or "lenacapavir" or "LEN" or "GS-CA1" or "Portmanteau inhibitor$" or "Fusion inhibitor$" or "albuvirtide" or "leronlimab" or "Eviplera" or "rilpivirine, emtricitabine and tenofovir disoproxil fumarate" or "attachment inhibitor" or "gp120 attachment inhibitor" or "Rukobia" or "fostemsavir" or "FTV" or INSTIs or "Vocabria" or "cabotegravir" or "CAB" or "cabenuva" or "Tybost" or "INSTI-based" or "INSTI-containing" or "INSTI-therap$" or "INSTI-treatment$" or "INSTI-regimen$").ti,ab.

52.Islatravir/ or Lenacapavir/ or exp Human immunodeficiency virus fusion inhibitor/ or Leronlimab/ or Labuvirtide/ or Emtricitabine plus rilpivirine plus tenofovir disoproxil/ or Fostemsavir/ or Cabotegravir/ or Cabotegravir plus rilpivirine/ or Cobicistat/

53. 28 or 29 or 30 or 31 or 32 or 33 or 34 or 35 or 36 or 37 or 38 or 39 or 40 or 41 or 42 or 43 or 44 or 45 or 46 or 47 or 48 or 49 or 50 or 51 or 52

54. 27 or 53

55.21 and 54

**To search for meta-analyses:**

56. systematic review*.ti.

57. meta analys*.ti.

58. metaanalys*.ti.

59. systematic.ti.

60. 56 or 57 or 58 or 59

61.55 and 60

**To search for randomized controlled trials:**

56. ((randomized controlled trial or controlled clinical trial).pt. or randomized.ab. or randomised.ab. or placebo.ab. or drug therapy.fs. or randomly.ab. or trial.ab. or groups.ab.) not (exp animals/ not humans.sh.)

57. 55 and 56

# Appendix 2: Cochrane risk of bias assessment of randomised controlled trials (RoB1)

| Study (Trial) | Sequence Generation | | Allocation concealment | | Blinding | | Incomplete outcome data | | Selective outcome reporting | | Other sources of bias | | Overall risk of bias |
| --- | --- | --- | --- | --- | --- | --- | --- | --- | --- | --- | --- | --- | --- |
|  |  | Specify |  | Specify |  | Specify |  | Specify |  | Specify |  | Specify |  |
| Lockman et al. 2021 (50) | Yes | Randomised 1:1:1 using central computerised randomisation system. Randomisation stratified by gestational age and country. | Yes | Central computerised system used to randomise. | No | Open label. Local staff and participants unmasked to treatment assignment | Yes | Numbers of missing outcome data balanced between trial arms. Similar underlying causes of missingness.  Intention-to-treat analysis. | Yes | Reported two interim result analyses as per protocol. | No | Gestational age assessed using USS dating 14-28 weeks | High |
| Kintu et al. 2020  (49) | Yes | Randomly assigned to trial arms (1:1) after meeting eligibility criteria. Stratified by country. | No | Randomly assigned by the study investigators. | No | Open label | Yes | Numbers of missing outcome data balanced between trial arms. Similar underlying causes of missingness.  Intention-to-treat analysis. | Yes | Reported one interim result analysis as per protocol. | No | Gestational age assessed using USS dating after 28 weeks | High |
| Joao et al. 2020 (48) | Yes | Randomised to trial arm with stratification by gestational age at enrolment, NNRTI backbone. | Yes | Web-based central computer used for randomisation. | No | Open label. Participants, site staff and statisticians unmasked to group assignment | Yes | High number of women excluded from primary efficacy analysis, but this was balanced between groups. Intention-to-treat analysis. | Yes | Reported two interim result analyses as per protocol. | No | Gestational age based on USS or LMP at 20-37 weeks | High |

# Appendix 3: Overall risk of bias of randomised controlled trials (RoB1)

| **Overall risk of bias** | **Explanation** |
| --- | --- |
| Low risk | Low risk of bias for all domains |
| Some concern | Some concerns in at least 1 domain, no domains assessed to be high risk |
| High risk | High risk in at least 1 domain or some concerns in two or more domains |

# Appendix 4: A Measurement Tool to Assess systematic Reviews, version 2 (AMSTAR 2)

| **Domain** | **Explanation** | **Critical or**  **non-critical** |
| --- | --- | --- |
| 1 | Did the research questions and inclusion criteria for the  review include the components of PICO? | Non-critical |
| 2 | Did the report of the review contain an explicit statement that the review methods were established prior to the conduct of the review and did the report justify any significant deviations from the protocol? | Critical |
| 3 | Did the review authors explain their selection of the study designs for inclusion in the review? | Non-critical |
| 4 | Did the review authors use a comprehensive literature search strategy? | Critical |
| 5 | Did the review authors perform study selection in duplicate? | Non-critical |
| 6 | Did the review authors perform data extraction in duplicate? | Non-critical |
| 7 | Did the review authors provide a list of excluded studies and justify the exclusions? | Critical |
| 8 | Did the review authors describe the included studies in adequate detail? | Non-critical |
| 9 | Did the review authors use a satisfactory technique for assessing the risk of bias (RoB) in individual studies that were included in the review? | Critical |
| 10 | Did the review authors report on the sources of funding for the studies included in the review? | Non-critical |
| 11 | If meta-analysis was performed did the review authors use appropriate methods for statistical combination of results? | Non-critical |
| 12 | If meta-analysis was performed, did the review authors assess the potential impact of RoB in individual studies on the results of the meta-analysis or other evidence synthesis? | Non-critical |
| 13 | Did the review authors account for RoB in individual studies when interpreting/ discussing the results of the review? | Critical |
| 14 | Did the review authors provide a satisfactory explanation for, and discussion of, any heterogeneity observed in the results of the review? | Non-critical |
| 15 | If they performed quantitative synthesis did the review authors carry out an adequate investigation of publication bias (small study bias) and discuss its likely impact on the results of the review? | Critical |
| 16 | Did the review authors report any potential sources of conflict of interest, including any funding they received for conducting the review? | Non-critical |

# Appendix 5: Overall AMSTAR 2 assessment

| **Overall quality of evidence** | **Explanation** |
| --- | --- |
| High | No more than one non-critical weakness (not starred) |
| Moderate | More than 1 non-critical weakness |
| Low | 1 critical flaw with or without non-critical weaknesses |
| Critically low | More than 1 critical flaw with or without non-critical weakness |

# Appendix 6: AMSTAR 2 assessment of meta-analyses

| Publication Author | Year of meta-analysis | 1 | 2* | 3 | 4* | 5 | 6 | 7* | 8 | 9* | 10 | 11* | 12 | 13* | 14 | 15* | 16 | Overall quality of evidence |
| --- | --- | --- | --- | --- | --- | --- | --- | --- | --- | --- | --- | --- | --- | --- | --- | --- | --- | --- |
| **Meta-analyses of cohort studies** | | | | | | | | | | | | | | | | | | |
| Beck et al (39) | 2024 | Yes | Yes | Yes | Yes | Yes | Yes | No | Yes | Yes | Yes | Yes | Yes | Yes | Yes | Yes | Yes | Low |
| Boering et al (40) | 2025 | Yes | Yes | Yes | Yes | Yes | Yes | No | Yes | Yes | Yes | Yes | Yes | Yes | No | Yes | Yes | Low |
| Cowdell et al (41) | 2022 | Yes | Yes | Yes | Yes | Yes | Yes | No | Yes | Yes | Yes | Yes | Yes | Yes | Yes | Yes | Yes | Low |
| Cowdell et al (42) | 2025 | Yes | Yes | Yes | Yes | Yes | Yes | No | Yes | Yes | Yes | Yes | Yes | Yes | Yes | Yes | Yes | Low |
| Hey et al (43) | 2025 | Yes | Yes | No | Yes | Yes | Yes | No | Yes | Yes | No | Yes | Yes | Yes | Yes | Yes | Yes | Low |
| Portwood et al (44) | 2022 | Yes | Yes | Yes | Yes | Yes | Yes | No | Yes | Yes | Yes | Yes | Yes | Yes | Yes | Yes | Yes | Low |
| Sexton et al (45) | 2023 | Yes | Yes | Yes | Yes | Yes | Yes | No | Yes | Yes | Yes | Yes | Yes | Yes | Yes | Yes | Yes | Low |
| Uthman et al (46) | 2017 | Yes | Yes | No | Yes | Yes | Yes | No | Yes | No | Yes | Yes | No | Yes | Yes | Yes | Yes | Critically low |
| Wedi et al (47) | 2016 | Yes | Yes | Yes | Yes | Yes | Yes | No | Yes | Yes | Yes | Yes | Yes | Yes | Yes | Yes | Yes | Low |
| **Network meta-analysis of randomised controlled rials** | | | | | | | | | | | | | | | | | | |
| Tshivuila-Matala et al (51) | 2020 | Yes | Yes | Yes | Yes | Yes | Yes | No | Yes | Yes | Yes | Yes | Yes | Yes | Yes | Yes | Yes | Low |

* critical domains

# Appendix 7: GRADE assessments for randomised controlled trials

| **Category** | **Criteria** | **Score** |
| --- | --- | --- |
| Risk of bias (based on RoB1) | No concerns | 0 |
|  | Some concerns | -1 |
|  | High risk of bias | -2 |
| Inconsistency | I^2^ <50% or single study | 0 |
|  | I^2^ 50–75% | -1 |
|  | I^2^ >75% | -2 |
| Indirectness | Majority of studies from low- and middle-income countries | 0 |
|  | >50% studies from high income countries | -1 |
|  | >75% studies from high income countries | -2 |
| Imprecision (cumulative score, maximum -2) | If confidence interval wider than 1 | -1 |
|  | < 300 participants | -1 |
| Publication bias | Peter’s test not significant | 0 |
|  | If Peter’s test not done as less than 10 studies | 0 |
|  | If Peter’s test significant | -1 |
| To upgrade score +1 | If relative risk >2 then upgrade +1, if >5 then upgrade +2 | |
|  | Upgrade if all plausible confounding factors were accounted for | |

Note: only randomised controlled trials with no major threats to validity (i.e. that have not been downgraded) can be upgraded

# Appendix 8: GRADE assessments for meta-analyses

| **Category** | **Criteria** | **Score** |
| --- | --- | --- |
| Risk of bias | <50% poor quality studies | 0 |
|  | 50-75% poor quality studies | -1 |
|  | >75% poor quality studies | -2 |
| Inconsistency | I^2^ <50% or single study | 0 |
|  | I^2^ 50–75% | -1 |
|  | I^2^ >75% | -2 |
| Indirectness | Majority of studies from low- and middle-income countries | 0 |
|  | >50% studies from high income countries | -1 |
|  | >75% studies from high income countries | -2 |
| Imprecision (cumulative score, maximum -2) | If confidence interval wider than 1 | -1 |
|  | Single study | -1 |
|  | < 1000 participants | -1 |
| Publication bias | Peter’s test not significant | 0 |
|  | If Peter’s test not done as fewer than 10 studies | 0 |
|  | If Peter’s test significant | -1 |
| To upgrade score +1 | If relative risk >2 then upgrade +1, if >5 then upgrade +2 | |
|  | Upgrade if all plausible confounding factors were accounted for | |

Note: only observational studies with no major threats to validity (i.e. that have not been downgraded) can be upgraded

# Appendix 9: GRADE overall assessment criteria

| **GRADE criteria** | **Rating**  (circle one) | **Footnotes**  (explain reasons for down- or upgrading) | **Quality of the evidence**  (Circle one) |
| --- | --- | --- | --- |
| **Reference:** | | | |
| **Outcome:** | | | |
| **Study design** | **RCT (starts as high quality)**  **Non-RCT (starts as low quality)** |  | ⊕⊕⊕⊕ High    ⊕⊕⊕🌕 Moderate  ⊕⊕🌕🌕 Low  ⊕🌕🌕🌕 Very Low |
| **Risk of Bias**  *(use the Cochrane Risk of Bias tables and figures)* | No  serious (-1)  very serious (-2) |  |  |
| **Inconsistency** | No  serious (-1)  very serious (-2) |  |  |
| **Indirectness** | No  serious (-1)  very serious (-2) |  |  |
| **Imprecision** | No  serious (-1)  very serious (-2) |  |  |
| **Publication Bias** | Undetected  Strongly suspected (-1) |  |  |
| **Other**  (upgrading factors, circle all that apply) | Large effect (+1 or +2)  Dose response (+1 or +2)  No Plausible confounding (+1 or +2) |  |  |

# Appendix 10: Risk of preterm birth of WLHIV receiving different PI drugs.

|  |  | **RR (95%CI)** | | | | | | | | |
| --- | --- | --- | --- | --- | --- | --- | --- | --- | --- | --- |
| **Cowdell et al, eClinicalMedicine, 2022 (41)** |  | ATV/r-ART | NFV-ART | DRV/r-ART | FPV/r-ART | IDV/r-ART | IDV-ART | SQV/r-ART | SQV-ART | TPV/r-ART |
|  | LPV/r-ART | 0.98 (0.75, 1.27) | 1.33 (1.03, 1.72) |  | 0.83 (0.36, 1.95) | 1.09 (0.54, 2.22) | 1.68 (0.43, 6.65) | 1.08 (0.65, 1.81) | 2.99 (0.43, 20.92) | 1.90 (0.13, 27.28) |
|  | ATV/r-ART |  | 1.63 (0.91, 2.92) | 0.92 (0.55, 1.55) | 0.90 (0.12, 6.98) | 0.54 (0.07, 4.04) | 2.70 (0.62, 11.72) | 0.54 (0.08, 3.77) | 4.80 (0.64, 35.91) | 3.10 (0.21, 46.34) |
|  | NFV-ART |  |  |  | 0.52 (0.20, 1.32) | 0.66 (0.28, 1.53) | 1.06 (0.27, 4.19) | 0.65 (0.33, 1.30) | 1.89 (0.27, 13.19) | 1.20 (0.08, 17.25) |
|  | FPV/r-ART |  |  |  |  | 1.44 (0.47, 4.42)) | 0.76 (0.04, 13.43) | 1.43 (0.52, 3.91) | 1.13 (0.05, 23.72) |  |
|  | IDV/r-ART |  |  |  |  |  |  | 0.99 (0.43, 2.31) |  |  |
|  | IDV-ART |  |  |  |  |  |  |  | 1.78 (0.18, 17.80) | 1.32 (0.07, 23.26) |
|  | SQV-ART |  |  |  |  |  |  |  |  | 0.88 (0.04, 18.47) |

# Appendix 11: Using the Grading of Recommendation, Assessment, Development, and Evaluation (GRADE) approach to assess the certainty of evidence

## Appendix 11.1: GRADE Assessment for Wedi et al, Lancet HIV, 2016

| WLHIV naïve to ART vs  HIV-negative women | Risk of bias | Inconsistency | Indirectness | Imprecision | Publication bias | Quality |
| --- | --- | --- | --- | --- | --- | --- |
| PTB | -1 | -2 | 0 | 0 | 0 | Very low |
| VPTB | -2 | 0 | 0 | -2 | 0 | Very low |
| LBW | 0 | -2 | 0 | 0 | 0 | Very low |
| VLBW | 0 | 0 | -2 | -2 | 0 | Very low |
| Term LBW | -1 | -2 | 0 | -1 | 0 | Very low |
| Preterm LBW | 0 | -2 | 0 | -1 | 0 | Very low |
| SGA | -1 | -1 | 0 | 0 | 0 | Very low |
| VSGA | -2 | 0 | 0 | -1 | 0 | Very low |
| SB | -1 | 0 | 0 | -1 | 0 | Very low |
| NND | 0 | -2 | 0 | 0 | 0 | Very low |

## Appendix 11.2: GRADE Assessment for Portwood et al, AIDS, 2022

| WLHIV with ART  vs  HIV-negative women | Risk of bias | Inconsistency | Indirectness | Imprecision | Publication bias | Quality |
| --- | --- | --- | --- | --- | --- | --- |
| PTB | 0 | -2 | 0 | 0 | 0 | Very low |
| VPTB | 0 | -2 | 0 | -1 | 0 | Very low |
| sPTB | 0 | 0 | -2 | -1 | 0 | Very low |
| LBW | 0 | -2 | 0 | 0 | 0 | Very low |
| VLBW | 0 | -2 | 0 | -1 | 0 | Very low |
| Term LBW | 0 | 0 | -1 | -1 | 0 | Very low |
| Preterm LBW | 0 | 0 | 0 | -1 | 0 | Very low |
| SGA | 0 | -2 | 0 | -1 | 0 | Very low |
| VSGA | 0 | 0 | 0 | 0 | 0 | Low |
| SB | 0 | 0 | -2 | -2 | 0 | Very low |
| NND | 0 | -1 | 0 | -1 | 0 | Very low |

| WLHIV with ART  vs  WLHIV naïve to ART | Risk of bias | Inconsistency | Indirectness | Imprecision | Publication bias | Quality |
| --- | --- | --- | --- | --- | --- | --- |
| PTB | -1 | -2 | -1 | 0 | 0 | Very low |
| VPTB | -1 | -2 | -1 | -1 | 0 | Very low |
| sPTB | 0 | 0 | -2 | 0 | 0 | Very low |
| LBW | -1 | -1 | 0 | 0 | 0 | Very low |
| VLBW | -2 | 0 | -2 | -1 | 0 | Very low |
| SGA | 0 | 0 | 0 | 0 | 0 | Low |
| VSGA | -2 | 0 | -2 | -2 | 0 | Very low |

## Appendix 11.3: GRADE Assessment for Hey et al, AIDS, 2025

| WLHIV with PI-ART  vs  HIV-negative women | Risk of bias | Inconsistency | Indirectness | Imprecision | Publication bias | Quality |
| --- | --- | --- | --- | --- | --- | --- |
| PTB | 0 | -1 | -1 | 0 | 0 | Very low |
| VPTB | 0 | -1 | -1 | -1 | 0 | Very low |
| sPTB | 0 | 0 | -1 | -1 | 0 | Very low |
| LBW | 0 | 0 | -1 | -1 | 0 | Very low |
| VLBW | 0 | 0 | -1 | -1 | 0 | Very low |
| Term LBW | -2 | 0 | 0 | -2 | 0 | Very low |
| SGA | 0 | -2 | -1 | -1 | 0 | Very low |
| VSGA | 0 | 0 | -1 | -1 | 0 | Very low |
| NND | 0 | 0 | -1 | -1 | 0 | Very low |

| WLHIV with NNRTI-ART  vs  HIV-negative women | Risk of bias | Inconsistency | Indirectness | Imprecision | Publication bias | Quality |
| --- | --- | --- | --- | --- | --- | --- |
| PTB | 0 | -2 | 0 | 0 | 0 | Very low |
| VPTB | 0 | -2 | 0 | -1 | 0 | Very low |
| LBW | 0 | -2 | 0 | -1 | 0 | Very low |
| VLBW | -1 | -2 | -1 | -1 | 0 | Very low |
| Term LBW | 0 | 0 | 0 | -2 | 0 | Very low |
| Preterm LBW | 0 | 0 | 0 | -2 | 0 | Very low |
| SGA | 0 | -2 | 0 | 0 | 0 | Very low |
| VSGA | 0 | -2 | 0 | 0 | 0 | Very low |
| SB | 0 | 0 | 0 | -2 | 0 | Very low |
| NND | 0 | -1 | 0 | 0 | 0 | Very low |

| WLHIV with INSTI-ART vs  HIV-negative women | Risk of bias | Inconsistency | Indirectness | Imprecision | Publication bias | Quality |
| --- | --- | --- | --- | --- | --- | --- |
| PTB | 0 | 0 | -1 | 0 | 0 | Very low |
| VPTB | 0 | 0 | 0 | -1 | 0 | Very low |
| SGA | 0 | 0 | 0 | -1 | 0 | Very low |
| VSGA | 0 | 0 | 0 | -1 | 0 | Very low |
| NND | 0 | 0 | 0 | -1 | 0 | Very low |

| WLHIV with EFV-ART  vs  HIV-negative women | Risk of bias | Inconsistency | Indirectness | Imprecision | Publication bias | Quality |
| --- | --- | --- | --- | --- | --- | --- |
| PTB | 0 | -1 | 0 | 0 | 0 | Very low |
| VPTB | 0 | 0 | 0 | 0 | 0 | Low |
| LBW | 0 | 0 | 0 | 0 | 0 | Low |
| VLBW | 0 | 0 | 0 | 0 | 0 | Low |
| Term LBW | 0 | 0 | 0 | -2 | 0 | Very low |
| Preterm LBW | 0 | 0 | 0 | -2 | 0 | Very low |
| SGA | 0 | 0 | 0 | 0 | 0 | Low |
| VSGA | 0 | 0 | 0 | 0 | 0 | Low |
| SB | 0 | 0 | 0 | -1 | 0 | Very low |
| NND | 0 | -1 | 0 | 0 | 0 | Very low |

| WLHIV with NVP-ART  vs  HIV-negative women | Risk of bias | Inconsistency | Indirectness | Imprecision | Publication bias | Quality |
| --- | --- | --- | --- | --- | --- | --- |
| PTB | 0 | -2 | 0 | 0 | 0 | Very low |
| VPTB | 0 | 0 | 0 | -1 | 0 | Very low |
| LBW | 0 | -2 | 0 | 0 | 0 | Very low |
| SGA | 0 | -2 | 0 | 0 | 0 | Very low |
| VSGA | 0 | 0 | 0 | -1 | 0 | Very low |
| NND | 0 | 0 | 0 | -1 | 0 | Very low |

## Appendix 11.4: GRADE Assessment for Cowdell et al, eClinicalMedicine, 2022

| WLHIV with PI-ART vs  WLHIV with non-PI-ART | Risk of bias | Inconsistency | Indirectness | Imprecision | Publication bias | Quality |
| --- | --- | --- | --- | --- | --- | --- |
| PTB | -1 | -1 | -1 | 0 | 0 | Very low |
| VPTB | 0 | 0 | -1 | 0 | 0 | Very low |
| sPTB | 0 | -2 | -1 | -1 | 0 | Very low |
| LBW | -1 | -1 | 0 | 0 | 0 | Very low |
| VLBW | -1 | 0 | -1 | 0 | 0 | Very low |
| Term LBW | -2 | 0 | -2 | -2 | 0 | Very low |
| SGA | 0 | -1 | -1 | 0 | 0 | Very low |
| VSGA | 0 | 0 | -1 | 0 | 0 | Very low |
| SB | -2 | 0 | -2 | -1 | 0 | Very low |
| NND | 0 | 0 | 0 | -2 | 0 | Very low |

| WLHIV with LPV/r-ART vs  WLHIV with ATV/r-ART | Risk of bias | Inconsistency | Indirectness | Imprecision | Publication bias | Quality |
| --- | --- | --- | --- | --- | --- | --- |
| PTB | 0 | 0 | -2 | 0 | 0 | Very low |
| VPTB | -2 | 0 | -2 | -2 | 0 | Very low |
| LBW | 0 | 0 | -1 | 0 | 0 | Very low |
| VLBW | -1 | -1 | -2 | -1 | 0 | Very low |
| SGA | 0 | 0 | -2 | -2 | 0 | Very low |
| VSGA | -2 | 0 | -2 | -1 | 0 | Very low |
| SB | 0 | 0 | -2 | -2 | 0 | Very low |

| WLHIV with ATV/r-ART  vs  WLHIV with DRV/r-ART | Risk of bias | Inconsistency | Indirectness | Imprecision | Publication bias | Quality |
| --- | --- | --- | --- | --- | --- | --- |
| PTB | 0 | 0 | -2 | -2 | 0 | Very low |
| VPTB | 0 | 0 | -2 | -2 | 0 | Very low |
| LBW | 0 | 0 | -2 | -2 | 0 | Very low |
| VLBW | 0 | 0 | -2 | -2 | 0 | Very low |
| SGA | 0 | 0 | -2 | -2 | 0 | Very low |
| VSGA | 0 | 0 | -2 | -2 | 0 | Very low |
| SB | -1 | 0 | -2 | 0 | 0 | Very low |

| WLHIV with LPV/r-ART  vs  WLHIV with DRV/r-ART | Risk of bias | Inconsistency | Indirectness | Imprecision | Publication bias | Quality |
| --- | --- | --- | --- | --- | --- | --- |
| VSGA | 0 | 0 | -2 | -2 | 0 | Very low |
| SB | -2 | 0 | -2 | -2 | 0 | Very low |

| WLHIV with LPV/r-ART  vs  WLHIV with NFV-ART | Risk of bias | Inconsistency | Indirectness | Imprecision | Publication bias | Quality |
| --- | --- | --- | --- | --- | --- | --- |
| PTB | 0 | 0 | -2 | -2 | 0 | Very low |
| LBW | 0 | 0 | 0 | -2 | 0 | Very low |
| VSGA | -2 | 0 | -2 | -2 | 0 | Very low |

| WLHIV with ATV/r-ART  vs  WLHIV with NFV-ART | Risk of bias | Inconsistency | Indirectness | Imprecision | Publication bias | Quality |
| --- | --- | --- | --- | --- | --- | --- |
| PTB | 0 | 0 | -2 | -2 | 0 | Very low |
| LBW | 0 | 0 | 0 | -2 | 0 | Very low |
| VSGA | -2 | 0 | -2 | -2 | 0 | Very low |

| WLHIV with boosted  PI-ART  vs  WLHIV with non-boosted PI-ART | Risk of bias | Inconsistency | Indirectness | Imprecision | Publication bias | Quality |
| --- | --- | --- | --- | --- | --- | --- |
| PTB | 0 | 0 | -2 | 0 | 0 | Very low |
| VPTB | 0 | 0 | -2 | -1 | 0 | Very low |
| sPTB | 0 | 0 | -2 | -1 | 0 | Very low |
| LBW | 0 | 0 | 0 | -2 | 0 | Very low |
| VSGA | -2 | 0 | -2 | -2 | 0 | Very low |

## Appendix 11.5: GRADE Assessment for Cowdell et al, Clinical Microbiology and Infection, 2025

| WLHIV with TDF-ART  vs  WLHIV with non-TDF-ART | Risk of bias | Inconsistency | Indirectness | Imprecision | Publication bias | Quality |
| --- | --- | --- | --- | --- | --- | --- |
| PTB | 0 | -1 | 0 | 0 | 0 | Very low |
| VPTB | 0 | -1 | 0 | 0 | 0 | Very low |
| LBW | -1 | 0 | -1 | 0 | 0 | Very low |
| VLBW | 0 | 0 | -2 | 0 | 0 | Very low |
| Term LBW | -2 | 0 | -2 | -1 | 0 | Very low |
| SGA | 0 | -2 | -1 | 0 | 0 | Very low |
| VSGA | 0 | -1 | 0 | 0 | 0 | Very low |
| SB | 0 | -1 | 0 | 0 | 0 | Very low |
| NND | -1 | 0 | 0 | 0 | 0 | Very low |

| WLHIV with ZDV-ART  vs  WLHIV with non-ZDV-ART | Risk of bias | Inconsistency | Indirectness | Imprecision | Publication bias | Quality |
| --- | --- | --- | --- | --- | --- | --- |
| PTB | 0 | -2 | -1 | 0 | 0 | Very low |
| VPTB | -1 | -1 | 0 | 0 | 0 | Very low |
| LBW | 0 | 0 | -1 | 0 | 0 | Very low |
| VLBW | 0 | 0 | -2 | -1 | 0 | Very low |
| Term LBW | -2 | 0 | -2 | -1 | 0 | Very low |
| SGA | 0 | -2 | 0 | 0 | 0 | Very low |
| VSGA | 0 | -1 | 0 | 0 | 0 | Very low |
| SB | -1 | -2 | -1 | -1 | 0 | Very low |
| NND | -1 | 0 | 0 | -1 | 0 | Very low |

| WLHIV with ABC-ART  vs  WLHIV with non-ABC-ART | Risk of bias | Inconsistency | Indirectness | Imprecision | Publication bias | Quality |
| --- | --- | --- | --- | --- | --- | --- |
| PTB | 0 | 0 | -2 | 0 | 0 | Very low |
| VPTB | -1 | -1 | -2 | -1 | 0 | Very low |
| LBW | 0 | 0 | -2 | 0 | 0 | Very low |
| VLBW | 0 | -1 | -2 | -1 | 0 | Very low |
| Term LBW | -1 | 0 | -2 | 0 | 0 | Very low |
| SGA | 0 | 0 | -2 | 0 | 0 | Very low |
| SB | -2 | 0 | -2 | -2 | 0 | Very low |

| WLHIV with ZDV+XTC-ART  vs  WLHIV with TDF+XTC-ART | Risk of bias | Inconsistency | Indirectness | Imprecision | Publication bias | Quality |
| --- | --- | --- | --- | --- | --- | --- |
| PTB | 0 | -2 | -1 | -1 | 0 | Very low |
| VPTB | -1 | -1 | -1 | -1 | 0 | Very low |
| LBW | -1 | 0 | -1 | 0 | 0 | Very low |
| VLBW | 0 | 0 | -2 | -2 | 0 | Very low |
| Term LBW | -2 | 0 | -2 | -2 | 0 | Very low |
| SGA | 0 | -2 | 0 | 0 | 0 | Very low |
| VSGA | 0 | -1 | 0 | 0 | 0 | Very low |
| SB | -1 | -2 | -1 | -1 | 0 | Very low |
| NND | -1 | 0 | 0 | -1 | 0 | Very low |

| WLHIV with ABC+XTC-ART  vs  WLHIV with TDF+XTC-ART | Risk of bias | Inconsistency | Indirectness | Imprecision | Publication bias | Quality |
| --- | --- | --- | --- | --- | --- | --- |
| PTB | 0 | 0 | -2 | 0 | 0 | Very low |
| VPTB | -1 | 0 | -2 | -1 | 0 | Very low |
| LBW | -1 | 0 | -2 | 0 | 0 | Very low |
| VLBW | 0 | 0 | -2 | -2 | 0 | Very low |
| Term LBW | -2 | 0 | -2 | -2 | 0 | Very low |
| SGA | 0 | 0 | -2 | 0 | 0 | Very low |
| SB | -2 | 0 | -2 | -2 | 0 | Very low |

| WLHIV with ABC+XTC-ART  vs  WLHIV with ZDV+XTC-ART | Risk of bias | Inconsistency | Indirectness | Imprecision | Publication bias | Quality |
| --- | --- | --- | --- | --- | --- | --- |
| PTB | -1 | 0 | -2 | 0 | 0 | Very low |
| VPTB | -2 | 0 | -2 | -2 | 0 | Very low |
| LBW | -2 | 0 | -2 | -1 | 0 | Very low |
| Term LBW | -2 | 0 | -2 | -1 | 0 | Very low |
| SGA | 0 | 0 | -2 | -1 | 0 | Very low |
| SB | -2 | 0 | -2 | -2 | 0 | Very low |

| WLHIV with ZDV+XTC+NVP  vs  WLHIV with TDF+XTC+NVP | Risk of bias | Inconsistency | Indirectness | Imprecision | Publication bias | Quality |
| --- | --- | --- | --- | --- | --- | --- |
| PTB | 0 | 0 | 0 | -1 | 0 | Very low |
| VPTB | 0 | 0 | 0 | -1 | 0 | Very low |
| SGA | 0 | 0 | 0 | -1 | 0 | Very low |
| VSGA | 0 | 0 | 0 | -1 | 0 | Very low |
| SB | -2 | 0 | -2 | -1 | 0 | Very low |
| NND | 0 | 0 | 0 | -1 | 0 | Very low |

| WLHIV with TAF-ART  vs  WLHIV with TDF-ART | Risk of bias | Inconsistency | Indirectness | Imprecision | Publication bias | Quality |
| --- | --- | --- | --- | --- | --- | --- |
| LBW | -2 | 0 | -2 | -2 | 0 | Very low |

## Appendix 11.6: GRADE Assessment for Beck et al, Frontiers in Medicine, 2024

### PI-ART compared to NNRTI-ART

| WLHIV with LPV/r-ART vs  WLHIV with NVP-ART | Risk of bias | Inconsistency | Indirectness | Imprecision | Publication bias | Quality |
| --- | --- | --- | --- | --- | --- | --- |
| PTB | 0 | -2 | 0 | 0 | 0 | Very low |
| VPTB | -1 | 0 | 0 | 0 | 0 | Very low |
| LBW | -1 | 0 | 0 | 0 | 0 | Very low |
| VLBW | -1 | -2 | 0 | 0 | 0 | Very low |
| SGA | 0 | -2 | -1 | -1 | 0 | Very low |
| VSGA | 0 | 0 | 0 | -1 | 0 | Very low |
| SB | -2 | 0 | -2 | -2 | 0 | Very low |
| NND | 0 | 0 | 0 | -2 | 0 | Very low |

| WLHIV with ATV/r-ART  vs  WLHIV with NVP-ART | Risk of bias | Inconsistency | Indirectness | Imprecision | Publication bias | Quality |
| --- | --- | --- | --- | --- | --- | --- |
| PTB | 0 | 0 | -2 | -2 | 0 | Very low |
| LBW | -1 | 0 | -1 | -2 | 0 | Very low |
| SB | -2 | 0 | -2 | -2 | 0 | Very low |

| WLHIV with NFV-ART vs  WLHIV with NVP-ART | Risk of bias | Inconsistency | Indirectness | Imprecision | Publication bias | Quality |
| --- | --- | --- | --- | --- | --- | --- |
| PTB | -2 | 0 | 0 | -2 | 0 | Very low |
| LBW | -2 | 0 | 0 | -2 | 0 | Very low |

| WLHIV with LPV/r-ART vs  WLHIV with EFV-ART | Risk of bias | Inconsistency | Indirectness | Imprecision | Publication bias | Quality |
| --- | --- | --- | --- | --- | --- | --- |
| PTB | -1 | -2 | 0 | -1 | 0 | Very low |
| VPTB | 0 | 0 | 0 | -2 | 0 | Very low |
| LBW | -2 | 0 | 0 | -2 | 0 | Very low |
| VLBW | -2 | 0 | 0 | -2 | 0 | Very low |
| SGA | 0 | -2 | 0 | 0 | 0 | Very low |
| VSGA | 0 | 0 | 0 | -2 | 0 | Very low |
| SB | -2 | 0 | -2 | -2 | 0 | Very low |
| NND | 0 | 0 | 0 | -2 | 0 | Very low |

| WLHIV with ATV/r-ART vs  WLHIV with EFV-ART | Risk of bias | Inconsistency | Indirectness | Imprecision | Publication bias | Quality |
| --- | --- | --- | --- | --- | --- | --- |
| LBW | -2 | 0 | 0 | -2 | 0 | Very low |
| SB | -2 | 0 | -2 | -2 | 0 | Very low |

| WLHIV with DRV/r-ART vs  WLHIV with EFV-ART | Risk of bias | Inconsistency | Indirectness | Imprecision | Publication bias | Quality |
| --- | --- | --- | --- | --- | --- | --- |
| SB | -2 | 0 | -2 | -2 | 0 | Very low |

### PI-ART compared to INSTI-ART

| WLHIV with ATV/r-ART vs  WLHIV with DTG-ART | Risk of bias | Inconsistency | Indirectness | Imprecision | Publication bias | Quality |
| --- | --- | --- | --- | --- | --- | --- |
| PTB | 0 | 0 | -2 | -2 | 0 | Very low |
| VPTB | 0 | 0 | -2 | -2 | 0 | Very low |
| LBW | 0 | 0 | -2 | -2 | 0 | Very low |
| VLBW | 0 | 0 | -2 | -2 | 0 | Very low |
| SGA | 0 | 0 | -2 | -2 | 0 | Very low |

| WLHIV with DRV/r-ART vs  WLHIV with DTG-ART | Risk of bias | Inconsistency | Indirectness | Imprecision | Publication bias | Quality |
| --- | --- | --- | --- | --- | --- | --- |
| PTB | 0 | 0 | -2 | -2 | 0 | Very low |
| VPTB | 0 | 0 | -2 | -2 | 0 | Very low |
| LBW | 0 | 0 | -2 | -2 | 0 | Very low |
| VLBW | 0 | 0 | -2 | -2 | 0 | Very low |
| SGA | 0 | 0 | -2 | -2 | 0 | Very low |

| WLHIV with LPV/r-ART vs  WLHIV with RAL-ART | Risk of bias | Inconsistency | Indirectness | Imprecision | Publication bias | Quality |
| --- | --- | --- | --- | --- | --- | --- |
| VSGA | 0 | 0 | 0 | -2 | 0 | Very low |

| WLHIV with ATV/r-ART vs  WLHIV with RAL-ART | Risk of bias | Inconsistency | Indirectness | Imprecision | Publication bias | Quality |
| --- | --- | --- | --- | --- | --- | --- |
| PTB | 0 | 0 | -2 | -2 | 0 | Very low |
| VPTB | 0 | 0 | -2 | -2 | 0 | Very low |
| LBW | 0 | 0 | -2 | -2 | 0 | Very low |
| VLBW | 0 | 0 | -2 | -2 | 0 | Very low |
| SGA | 0 | 0 | -2 | -2 | 0 | Very low |
| VSGA | 0 | 0 | -2 | -2 | 0 | Very low |

| WLHIV with DRV/r-ART vs  WLHIV with RAL-ART | Risk of bias | Inconsistency | Indirectness | Imprecision | Publication bias | Quality |
| --- | --- | --- | --- | --- | --- | --- |
| PTB | 0 | 0 | -2 | -2 | 0 | Very low |
| VPTB | 0 | 0 | -2 | -2 | 0 | Very low |
| LBW | 0 | 0 | -2 | -2 | 0 | Very low |
| VLBW | 0 | 0 | -2 | -2 | 0 | Very low |
| SGA | 0 | 0 | -2 | -2 | 0 | Very low |
| VSGA | 0 | 0 | -2 | -2 | 0 | Very low |

| WLHIV with ATV/r-ART vs  WLHIV with EVG/c-ART | Risk of bias | Inconsistency | Indirectness | Imprecision | Publication bias | Quality |
| --- | --- | --- | --- | --- | --- | --- |
| PTB | 0 | 0 | -2 | -2 | 0 | Very low |
| VPTB | 0 | 0 | -2 | -2 | 0 | Very low |
| LBW | 0 | 0 | -2 | -2 | 0 | Very low |
| VLBW | 0 | 0 | -2 | -2 | 0 | Very low |
| SGA | 0 | 0 | -2 | -2 | 0 | Very low |

| WLHIV with DRV/r-ART vs  WLHIV with EVG/c-ART | Risk of bias | Inconsistency | Indirectness | Imprecision | Publication bias | Quality |
| --- | --- | --- | --- | --- | --- | --- |
| PTB | 0 | 0 | -2 | -2 | 0 | Very low |
| VPTB | 0 | 0 | -2 | -2 | 0 | Very low |
| LBW | 0 | 0 | -2 | -2 | 0 | Very low |
| VLBW | 0 | 0 | -2 | -2 | 0 | Very low |
| SGA | 0 | 0 | -2 | -2 | 0 | Very low |

### NNRTI-ART compared to NRTI-ART

| WLHIV with NVP-ART vs  WLHIV with ABC-ART | Risk of bias | Inconsistency | Indirectness | Imprecision | Publication bias | Quality |
| --- | --- | --- | --- | --- | --- | --- |
| PTB | -2 | 0 | 0 | -2 | 0 | Very low |

### INSTI-ART compared to NNRTI-ART

| WLHIV with DTG- ART  vs  WLHIV with EFV-ART | Risk of bias | Inconsistency | Indirectness | Imprecision | Publication bias | Quality |
| --- | --- | --- | --- | --- | --- | --- |
| PTB | 0 | 0 | 0 | 0 | 0 | Low |
| VPTB | -1 | 0 | 0 | 0 | 0 | Very low |
| SGA | 0 | 0 | 0 | 0 | 0 | Low |
| VSGA | -1 | 0 | 0 | 0 | 0 | Very low |
| NND | 0 | 0 | 0 | -1 | 0 | Very low |

| WLHIV with RAL-ART vs  WLHIV with EFV-ART | Risk of bias | Inconsistency | Indirectness | Imprecision | Publication bias | Quality |
| --- | --- | --- | --- | --- | --- | --- |
| PTB | 0 | 0 | 0 | -2 | 0 | Very low |
| LBW | 0 | 0 | 0 | -2 | 0 | Very low |
| SGA | 0 | 0 | 0 | -2 | 0 | Very low |

| WLHIV with DTG- ART vs  WLHIV with RPV-ART | Risk of bias | Inconsistency | Indirectness | Imprecision | Publication bias | Quality |
| --- | --- | --- | --- | --- | --- | --- |
| PTB | 0 | 0 | -2 | -2 | 0 | Very low |
| VPTB | 0 | 0 | -2 | -2 | 0 | Very low |
| LBW | 0 | 0 | -2 | -2 | 0 | Very low |
| VLBW | 0 | 0 | -2 | -2 | 0 | Very low |
| SGA | 0 | 0 | -2 | -2 | 0 | Very low |

## Appendix 11.7: GRADE Assessment for Boering et al, AIDS, 2025

| WLHIV with preconception ART initiation  vs  HIV-negative women | Risk of bias | Inconsistency | Indirectness | Imprecision | Publication bias | Quality |
| --- | --- | --- | --- | --- | --- | --- |
| PTB | 0 | -2 | 0 | 0 | 0 | Very low |
| VPTB | 0 | -2 | 0 | -1 | 0 | Very low |
| sPTB | 0 | 0 | 0 | -1 | 0 | Very low |
| LBW | 0 | -2 | 0 | -1 | 0 | Very low |
| VLBW | 0 | -1 | 0 | -1 | 0 | Very low |
| SGA | 0 | -2 | 0 | -1 | 0 | Very low |
| VSGA | 0 | -2 | 0 | -1 | 0 | Very low |
| SB | 0 | 0 | 0 | -2 | 0 | Very low |
| NND | 0 | 0 | 0 | -1 | 0 | Very low |

| WLHIV with antenatal ART initiation  vs  HIV-negative women | Risk of bias | Inconsistency | Indirectness | Imprecision | Publication bias | Quality |
| --- | --- | --- | --- | --- | --- | --- |
| PTB | 0 | -2 | 0 | 0 | -1 | Very low |
| VPTB | 0 | -2 | 0 | -1 | 0 | Very low |
| sPTB | 0 | 0 | 0 | -1 | 0 | Very low |
| LBW | 0 | -2 | 0 | -1 | 0 | Very low |
| VLBW | 0 | 0 | 0 | -1 | 0 | Very low |
| SGA | 0 | -2 | 0 | -1 | 0 | Very low |
| VSGA | 0 | 0 | 0 | 0 | 0 | Low |
| SB | 0 | 0 | 0 | -2 | 0 | Very low |
| NND | 0 | 0 | 0 | -1 | 0 | Very low |

| WLHIV with preconception ART initiation  vs  WLHIV naïve to ART | Risk of bias | Inconsistency | Indirectness | Imprecision | Publication bias | Quality |
| --- | --- | --- | --- | --- | --- | --- |
| PTB | -1 | -1 | 0 | 0 | 0 | Very low |
| LBW | 0 | -1 | 0 | -1 | 0 | Very low |
| SGA | 0 | 0 | 0 | 0 | 0 | Low |
| VSGA | -2 | 0 | 0 | -2 | 0 | Very low |

| WLHIV with antenatal ART initiation  vs  WLHIV naïve to ART | Risk of bias | Inconsistency | Indirectness | Imprecision | Publication bias | Quality |
| --- | --- | --- | --- | --- | --- | --- |
| PTB | 0 | -2 | 0 | 0 | -1 | Very low |
| LBW | 0 | -2 | 0 | -1 | 0 | Very low |
| SGA | 0 | 0 | 0 | 0 | 0 | Low |
| VSGA | -2 | 0 | 0 | -2 | 0 | Very low |
| SB | 0 | 0 | 0 | -2 | 0 | Very low |

## Appendix 11.8: GRADE Assessment for Uthman et al, Lancet HIV, 2017

| WLHIV with preconception ART initiation  vs  WLHIV with antenatal ART initiation | Risk of bias | Inconsistency | Indirectness | Imprecision | Publication bias | Quality |
| --- | --- | --- | --- | --- | --- | --- |
| PTB | -2 | -2 | -1 | 0 | 0 | Very low |
| LBW | -2 | 0 | 0 | 0 | 0 | Very low |
| VLBW | -2 | 0 | 0 | 0 | 0 | Very low |
| SGA | -1 | 0 | 0 | 0 | 0 | Very low |
| VSGA | -2 | 0 | 0 | 0 | 0 | Very low |

## Appendix 11.9: GRADE Assessment for Sexton et al, HIV Medicine, 2023

| WLHIV with preconception ART initiation  vs  WLHIV with antenatal ART initiation | Risk of bias | Inconsistency | Indirectness | Imprecision | Publication bias | Quality |
| --- | --- | --- | --- | --- | --- | --- |
| PTB | -2 | -2 | 0 | 0 | 0 | Very low |
| VPTB | 0 | 0 | 0 | 0 | 0 | Low |
| LBW | -1 | -1 | 0 | 0 | 0 | Very low |
| VLBW | 0 | 0 | 0 | 0 | 0 | Low |
| SGA | -1 | -2 | -1 | 0 | 0 | Very low |
| VSGA | 0 | -1 | 0 | 0 | 0 | Very low |
| NND | 0 | 0 | 0 | 0 | 0 | Low |

## Appendix 11.10: GRADE Assessment for Joao et al, Lancet HIV 2020

| WLHIV with ZDV/3TC/RAL vs WLHIV with ZDV/3TC/EFV | Risk of bias | Inconsistency | Indirectness | Imprecision | Publication bias | Quality |
| --- | --- | --- | --- | --- | --- | --- |
| PTB | -2 | 0 | 0 | -1 | 0 | Very low |
| VPTB | -2 | 0 | 0 | -1 | 0 | Very low |
| LBW | -2 | 0 | 0 | -1 | 0 | Very low |
| VLBW | -2 | 0 | 0 | -1 | 0 | Very low |
| SB | -2 | 0 | 0 | -1 | 0 | Very low |
| Vertical HIV transmission | -2 | 0 | 0 | -1 | 0 | Very low |

## Appendix 11.11: GRADE Assessment for Kintu et al, Lancet HIV 2020

| WLHIV with  TDF/(FTC/3TC)/DTG  Vs WLHIV with TDF/(FTC/3TC)/EFV | Risk of bias | Inconsistency | Indirectness | Imprecision | Publication bias | Quality |
| --- | --- | --- | --- | --- | --- | --- |
| PTB | -2 | 0 | 0 | -2 | 0 | Very low |
| SB | -2 | 0 | 0 | -2 | 0 | Very low |
| Vertical HIV transmission | -2 | 0 | 0 | -2 | 0 | Very low |

## Appendix 11.12: GRADE Assessment for Lockman et al, Lancet 2020

| WLHIV with TAF/FTC/DTG  vs  WLHIV with TDF/FTC/DTG | Risk of bias | Inconsistency | Indirectness | Imprecision | Publication bias | Quality |
| --- | --- | --- | --- | --- | --- | --- |
| PTB | -2 | 0 | 0 | 0 | 0 | Low |
| LBW | -2 | 0 | 0 | -1 | 0 | Very low |
| VLBW | -2 | 0 | 0 | -1 | 0 | Very low |
| SGA | -2 | 0 | 0 | 0 | 0 | Low |
| SB | -2 | 0 | 0 | -1 | 0 | Very low |
| NND | -2 | 0 | 0 | -1 | 0 | Very low |
| Vertical HIV transmission | -2 | 0 | 0 | -1 | 0 | Very low |

| WLHIV with TDF/FTC/DTG  vs  WLHIV with TDF/FTC/EFV | Risk of bias | Inconsistency | Indirectness | Imprecision | Publication bias | Quality |
| --- | --- | --- | --- | --- | --- | --- |
| PTB | -2 | 0 | 0 | 0 | 0 | Low |
| LBW | -2 | 0 | 0 | 0 | 0 | Low |
| VLBW | -2 | 0 | 0 | -1 | 0 | Very low |
| SGA | -2 | 0 | 0 | 0 | 0 | Low |
| SB | -2 | 0 | 0 | -1 | 0 | Very low |
| NND | -2 | 0 | 0 | 0 | 0 | Low |
| Vertical HIV transmission | -2 | 0 | 0 | -1 | 0 | Very low |

| WLHIV with TAF/FTC/DTG  vs  WLHIV with TDF/FTC/EFV | Risk of bias | Inconsistency | Indirectness | Imprecision | Publication bias | Quality |
| --- | --- | --- | --- | --- | --- | --- |
| PTB | -2 | 0 | 0 | 0 | 0 | Low |
| LBW | -2 | 0 | 0 | 0 | 0 | Low |
| VLBW | -2 | 0 | 0 | -1 | 0 | Very low |
| SGA | -2 | 0 | 0 | 0 | 0 | Low |
| SB | -2 | 0 | 0 | -1 | 0 | Very low |
| NND | -2 | 0 | 0 | 0 | 0 | Low |
| Vertical HIV transmission | -2 | 0 | 0 | -1 | 0 | Very low |

## Appendix 11.13: GRADE Assessment for Tshivuila-Matala et al, AIDS, 2020

### WLHIV receiving different ART regimens compared to WLHIV receiving placebo

| WLHIV with ZDV/3TC/ABC  vs  WLHIV with placebo | | Risk of bias | Inconsistency | Indirectness | Publication bias | Intransivity | Imprecision | Quality |
| --- | --- | --- | --- | --- | --- | --- | --- | --- |
| PTB | | -2 | 0 | 0 | 0 | -1 | -1 | Very low |
| LBW | | -2 | 0 | 0 | 0 | -1 | -1 | Very low |
| VLBW | | -2 | 0 | 0 | 0 | -1 | -1 | Very low |
| Vertical HIV transmission | Loop 1 | -2 | 0 | -2 | 0 | -1 | -1 | Very low |
|  | Loop 2 | -2 | 0 | 0 | 0 |  |  |  |
|  | Loop 3 | -2 | 0 | 0 | 0 |  |  |  |

| WLHIV with ZDV/3TC/EFV  vs  WLHIV with placebo | | Risk of bias | Inconsistency | Indirectness | Publication bias | Intransivity | Imprecision | Quality |
| --- | --- | --- | --- | --- | --- | --- | --- | --- |
| PTB | | -2 | 0 | 0 | 0 | -1 | -1 | Very low |
| LBW | | -2 | 0 | 0 | 0 | -1 | -1 | Very low |
| Vertical HIV transmission | Loop 1 | -2 | 0 | -2 | 0 | -1 | 0 | Very low |
|  | Loop 2 | -2 | 0 | 0 | 0 |  |  |  |
|  | Loop 3 | -2 | 0 | 0 | 0 |  |  |  |

| WLHIV with TDF/FTC/LPV/r  vs  WLHIV with placebo | | Risk of bias | Inconsistency | Indirectness | Publication bias | Intransivity | Imprecision | Quality |
| --- | --- | --- | --- | --- | --- | --- | --- | --- |
| PTB | Loop 1 | -2 | 0 | -2 | 0 | -2 | -2 | Very low |
|  | Loop 2 | -2 | 0 | 0 | 0 |  |  |  |
| LBW | Loop 1 | -2 | 0 | -2 | 0 | -1 | -2 | Very low |
|  | Loop 2 | -2 | 0 | 0 | 0 |  |  |  |
| VLBW | Loop 1 | -2 | 0 | -2 | 0 | -2 | -2 | Very low |
|  | Loop 2 | -2 | 0 | 0 | 0 |  |  |  |
| Vertical HIV transmission | Loop 1 | -2 | 0 | -2 | 0 | -2 | 0 | Very low |
|  | Loop 2 | -2 | 0 | 0 | 0 |  |  |  |

| WLHIV with ZDV/3TC/LPV/r  vs  WLHIV with placebo | | Risk of bias | Inconsistency | Indirectness | Publication bias | Intransivity | Imprecision | Quality | |
| --- | --- | --- | --- | --- | --- | --- | --- | --- | --- |
| PTB | | -2 | 0 | 0 | 0 | -1 | 0 | Very low | |
| LBW | | -2 | 0 | 0 | 0 | -1 | 0 | Very low | |
| VLBW | | -2 | 0 | 0 | 0 | -1 | 0 | Very low | |
| Vertical HIV transmission | Loop 1 | -2 | 0 | -2 | 0 | -1 | 0 | Very low |  |
|  | Loop 2 | -2 | 0 | 0 | 0 |  |  |  |  |

### Comparisons of WLHIV receiving different ART regimens

| WLHIV with ZDV/3TC/EFV  vs  WLHIV with ZDV/3TC/ABC | | Risk of bias | Inconsistency | Indirectness | Publication bias | Intransivity | Imprecision | Quality |
| --- | --- | --- | --- | --- | --- | --- | --- | --- |
| PTB | Loop 1 | -2 | 0 | 0 | 0 | -1 | -2 | Very low |
|  | Loop 2 | -2 | 0 | 0 | 0 |  |  |  |
| VPTB | Loop 1 | -2 | 0 | 0 | 0 | -1 | -2 | Very low |
|  | Loop 2 | -2 | 0 | 0 | 0 |  |  |  |
| sPTB | Loop 1 | -2 | 0 | 0 | 0 | -1 | -1 | Very low |
|  | Loop 2 | -2 | 0 | 0 | 0 |  |  |  |
| LBW | Loop 1 | -2 | 0 | 0 | 0 | -1 | -2 | Very low |
|  | Loop 2 | -2 | 0 | 0 | 0 |  |  |  |
| Vertical HIV transmission | Loop 1 | -2 | 0 | 0 | 0 | -1 | -2 | Very low |
|  | Loop 2 | -2 | 0 | 0 | 0 |  |  |  |

| WLHIV with TDF/FTC/LPV/  vs  WLHIV with ZDV/3TC/ABC | | Risk of bias | Inconsistency | Indirectness | Publication bias | Intransivity | Imprecision | Quality |
| --- | --- | --- | --- | --- | --- | --- | --- | --- |
| PTB | Loop 1 | -2 | 0 | 0 | 0 | -1 | -1 | Very low |
|  | Loop 2 | -2 | 0 | 0 | 0 |  |  |  |
| LBW | Loop 1 | -2 | 0 | 0 | 0 | -1 | -1 | Very low |
|  | Loop 2 | -2 | 0 | 0 | 0 |  |  |  |
| VLBW | Loop 1 | -2 | 0 | 0 | 0 | -1 | -2 | Very low |
|  | Loop 2 | -2 | 0 | 0 | 0 |  |  |  |
| Vertical HIV transmission | Loop 1 | -2 | 0 | 0 | 0 | -1 | -2 | Very low |
|  | Loop 2 | -2 | 0 | 0 | 0 |  |  |  |

| WLHIV with ZDV/3TC/LPV/r  vs  WLHIV with ZDV/3TC/ABC | Risk of bias | Inconsistency | Indirectness | Imprecision | Publication bias | Quality |
| --- | --- | --- | --- | --- | --- | --- |
| PTB | -2 | 0 | 0 | -1 | 0 | Very low |
| VPTB | -2 | 0 | 0 | -2 | 0 | Very low |
| sPTB | -2 | 0 | 0 | -1 | 0 | Very low |
| LBW | -2 | 0 | 0 | -1 | 0 | Very low |
| VLBW | -2 | 0 | 0 | -2 | 0 | Very low |
| Vertical HIV transmission | -2 | 0 | 0 | -1 | 0 | Very low |

| WLHIV with TDF/FTC/LPV/r  vs  WLHIV with ZDV/3TC/EFV | | Risk of bias | Inconsistency | Indirectness | Publication bias | Intransivity | Imprecision | Quality |
| --- | --- | --- | --- | --- | --- | --- | --- | --- |
| PTB | Loop 1 | -2 | 0 | 0 | 0 | -1 | -2 | Very low |
|  | Loop 2 | -2 | 0 | 0 | 0 |  |  |  |
| LBW | Loop 1 | -2 | 0 | 0 | 0 | -1 | -2 | Very low |
|  | Loop 2 | -2 | 0 | 0 | 0 |  |  |  |
| Vertical HIV transmission | Loop 1 | -2 | 0 | 0 | 0 | -1 | -2 | Very low |
|  | Loop 2 | -2 | 0 | 0 | 0 |  |  |  |

| WLHIV with ZDV/3TC/LPV/r  vs  WLHIV with ZDV/3TC/EFV | Risk of bias | Inconsistency | Indirectness | Imprecision | Publication bias | Quality |
| --- | --- | --- | --- | --- | --- | --- |
| PTB | -2 | 0 | 0 | -1 | 0 | Very low |
| VPTB | -2 | 0 | 0 | -2 | 0 | Very low |
| sPTB | -2 | 0 | 0 | -1 | 0 | Very low |
| LBW | -2 | 0 | 0 | -1 | 0 | Very low |
| Vertical HIV transmission | -2 | 0 | 0 | -1 | 0 | Very low |

| WLHIV with ZDV/3TC/LPV/r  vs  WLHIV with TDF/FTC/LPV/r | Risk of bias | Inconsistency | Indirectness | Imprecision | Publication bias | Quality |
| --- | --- | --- | --- | --- | --- | --- |
| PTB | -2 | 0 | 0 | -1 | 0 | Very low |
| LBW | -2 | 0 | 0 | -1 | 0 | Very low |
| VLBW | -2 | 0 | 0 | -2 | 0 | Very low |
| Vertical HIV transmission | -2 | 0 | 0 | -2 | 0 | Very low |

# Appendix 12: Excluded studies

## Appendix 12.1 Excluded studies: meta-analysis search

**Inappropriate population: 38**

Adane, H. A., Assefa, N., Mengistie, B., & Demis, A. (2020). Male involvement in prevention of mother to child transmission of human immunodeficiency virus and associated factors in Enebsiesarmider District, north West Ethiopia, 2018: a cross-sectional study. BMC Pregnancy & Childbirth, 20(1), 144.

Ades, V. (2011). Safety, pharmacokinetics and efficacy of artemisinins in pregnancy [Review]. Infectious Disease Reports, 3(1), e8.

Afran, L., Garcia Knight, M., Nduati, E., Urban, B. C., Heyderman, R. S., & Rowland-Jones, S. L. (2014). HIV-exposed uninfected children: a growing population with a vulnerable immune system? Clinical & Experimental Immunology, 176(1), 11-22.

Aguti, I., Kimbugwe, C., Apai, P., Munyaga, S., & Nyeko, R. (2020). HIV-free survival among breastfed infants born to HIV-positive women in northern Uganda: a facility-based retrospective study [Multicenter Study]. The Pan African medical journal, 37, 297.

Alemu, F. M., & Yalew, A. W. (2021). Does antiretroviral therapy cause congenital malformations? A systematic review and meta-analysis. Epidemiology and health, 43, e2021008.

Amin, O., Powers, J., Bricker, K. M., & Chahroudi, A. (2021). Understanding Viral and Immune Interplay During Vertical Transmission of HIV: Implications for Cure. Frontiers in Immunology, 12, 757400.

Antony, K. M., Kazembe, P. N., Pace, R. M., Levison, J., Phiri, H., Chiudzu, G., Harris, R. A., Chirwa, R., Nyondo, M., Marko, E., Chigayo, A., Nanthuru, D., Banda, B., Twyman, N., Ramin, S. M., Raine, S. P., Belfort, M. A., & Aagaard, K. M. (2020). Population-Based Estimation of the Preterm Birth Rate in Lilongwe, Malawi: Making Every Birth Count. American journal of perinatology reports, 10(1), e78-e86.

Athar, F., Ehsan, M., Farooq, M., Lo, K. B., Cheema, H. A., Ahmad, S., Naveed, A., & Umer, M. (2022). Adverse fetal and neonatal outcomes following in-utero exposure to oxcarbazepine: A systematic review and meta-analysis. British Journal of Clinical Pharmacology, 88(8), 3600-3609.

Augusto, O., Stergachis, A., Dellicour, S., Tinto, H., Vala, A., Ruperez, M., Macete, E., Nakanabo-Diallo, S., Kazienga, A., Valea, I., d'Alessandro, U., Ter Kuile, F. O., Calip, G. S., Ouma, P., Desai, M., & Sevene, E. (2020). First trimester use of artemisinin-based combination therapy and the risk of low birth weight and small for gestational age. Malaria Journal, 19(1), 144.

Auriti, C., De Rose, D. U., Santisi, A., Martini, L., Piersigilli, F., Bersani, I., Ronchetti, M. P., & Caforio, L. (2021). Pregnancy and viral infections: Mechanisms of fetal damage, diagnosis and prevention of neonatal adverse outcomes from cytomegalovirus to SARS-CoV-2 and Zika virus. Biochimica et Biophysica Acta - Molecular Basis of Disease, 1867(10), 166198.

Bafa, T. A., & Egata, A. D. (2020). Seroepidemiological patterns and predictors of hepatitis B, C and HIV viruses among pregnant women attending antenatal care clinic of Atat Hospital, Southern Ethiopia. SAGE Open Medicine, 8, 2050312119900870.

Bearden, A., Van Winden, K., Frederick, T., Kono, N., Operskalski, E., Pandian, R., Barton, L., Stek, A., & Kovacs, A. (2020). Low maternal vitamin D is associated with increased risk of congenital and peri/postnatal transmission of Cytomegalovirus in women with HIV. PLoS ONE [Electronic Resource], 15(2), e0228900.

Bhattacharya, D., Guo, R., Tseng, C. H., Emel, L., Sun, R., Chiu, S. H., Stranix-Chibanda, L., Chipato, T., Mohtashemi, N. Z., Kintu, K., Manji, K. P., Moodley, D., Thio, C. L., Maldonado, Y., & Currier, J. S. (2021). Maternal HBV Viremia and Association With Adverse Infant Outcomes in Women Living With HIV and HBV. Pediatric Infectious Disease Journal, 40(2), e56-e61.

Bukkems, V. E., Smolders, E. J., Jourdain, G., Burger, D. M., Colbers, A. P., Cressey, T. R., Network, P., & i, T. A. P. S. G. (2021). Effect of Pregnancy and Concomitant Antiretrovirals on the Pharmacokinetics of Tenofovir in Women With HIV Receiving Tenofovir Disoproxil Fumarate-Based Antiretroviral Therapy Versus Women With HBV Receiving Tenofovir Disoproxil Fumarate Monotherapy. Journal of Clinical Pharmacology, 61(3), 388-393.

Clark, R. (2008). Considerations for the antiretroviral management of women in 2008 [Review]. Women's health, 4(5), 465-477.

Ekali, G. L., Jesson, J., Enok, P. B., & Leroy, V. (2019). Effect of in utero exposure to HIV and antiretroviral drugs on growth in HIV-exposed uninfected children: a systematic review and meta-analysis protocol. BMJ Open, 9(6), e023937.

Erlwanger, A., Rocroi, I., Kirtley, S., & Hemelaar, J. (2024). Perinatal outcomes associated with pre-exposure prophylaxis for HIV prevention during pregnancy: a systematic review and meta-analysis. EClinicalMedicine, 70, 102532.

Ford, N., Calmy, A., & Mofenson, L. (2011). Safety of efavirenz in the first trimester of pregnancy: an updated systematic review and meta-analysis. AIDS, 25(18), 2301-2304.

Ford, N., Mofenson, L., Shubber, Z., Calmy, A., Andrieux-Meyer, I., Vitoria, M., Shaffer, N., & Renaud, F. (2014). Safety of efavirenz in the first trimester of pregnancy: an updated systematic review and meta-analysis. AIDS, 28 Suppl 2, S123-131.

Foster, C., Lyall, H., Olmscheid, B., Pearce, G., Zhang, S., & Gibb, D. M. (2009). Tenofovir disoproxil fumarate in pregnancy and prevention of mother-to-child transmission of HIV-1: is it time to move on from zidovudine? [Review]. HIV Medicine, 10(7), 397-406.

Giraud, A., Stephens, C. M., Boylan, G. B., & Walsh, B. H. (2022). The impact of perinatal inflammation on the electroencephalogram in preterm infants: a systematic review. Pediatric Research, 92(1), 32-39.

Heffron, R., Pintye, J., Matthews, L. T., Weber, S., & Mugo, N. (2016). PrEP as Peri-conception HIV Prevention for Women and Men. Current HIV/AIDS Reports, 13(3), 131-139.

Heidari, S., Mofenson, L., Cotton, M. F., Marlink, R., Cahn, P., & Katabira, E. (2011). Antiretroviral drugs for preventing mother-to-child transmission of HIV: a review of potential effects on HIV-exposed but uninfected children [Review]. Journal of Acquired Immune Deficiency Syndromes: JAIDS, 57(4), 290-296.

Hurt K, Rakovic J, Matecha J, Mojhova M, Zahalka F, Zikan M. HIV infection and adverse perinatal outcomes - a meta-analysis of premature births, low birth weights, and small for gestational age newborns. Ceska Gynekologie 2025; 90(4): 269-77.

Koivu, A. M., Nasanen-Gilmore, P. K., Hunter, P. J., Muthiani, Y., Isojarvi, J., Heimonen, O., Bastola, K., Csonka, L., Ashorn, P., & Ashorn, U. (2023). Antenatal interventions to address harmful behaviors and psychosocial risk factors in the prevention of low birth weight. American Journal of Clinical Nutrition, 117 Suppl 2, S148-S159.

Krist, A. H., & Crawford-Faucher, A. (2002). Management of newborns exposed to maternal HIV infection [Review]. American Family Physician, 65(10), 2049-2056.

Lehman, D. A., & Farquhar, C. (2007). Biological mechanisms of vertical human immunodeficiency virus (HIV-1). Reviews in Medical Virology, 17(6), 381-403.

Meleski, M. E., & Damato, E. G. (2003). HIV exposure: neonatal considerations [Review]. JOGNN - Journal of Obstetric, Gynecologic, & Neonatal Nursing, 32(1), 109-116.

Mendel, B., Kohar, K., Yumnanisha, D. A., Djiu, R. J., Winarta, J., Prakoso, R., & Siagian, S. N. (2024). Impact of fetal pulmonary valvuloplasty in in-utero critical pulmonary stenosis: A systematic review and meta-analysis. International Journal of Cardiology Congenital Heart Disease, 15, 100485.

Moberg, T., Van der Veeken, L., Persad, E., Hansson, S. R., & Bruschettini, M. (2023). Placenta-associated adverse pregnancy outcomes in women experiencing mild or severe hyperemesis gravidarum - a systematic review and meta-analysis. BMC Pregnancy & Childbirth, 23(1), 375.

Mofenson, L. M. (2010). Antiretroviral drugs to prevent breastfeeding HIV transmission. Antiviral Therapy, 15(4), 537-553.

Pennell, P. B. (2003). The importance of monotherapy in pregnancy. Neurology, 60(11 Suppl 4), S31-38.

Poirier, M. C., Gibbons, A. T., Rugeles, M. T., Andre-Schmutz, I., & Blanche, S. (2015). Fetal consequences of maternal antiretroviral nucleoside reverse transcriptase inhibitor use in human and nonhuman primate pregnancy. Current Opinion in Pediatrics, 27(2), 233-239.

Rakhmanina, N. Y., & van den Anker, J. N. (2014). Pharmacologic prevention of perinatal HIV infection. Early Human Development, 90 Suppl 1, S13-15.

Rothman, R. E. (2004). Current Centers for Disease Control and Prevention guidelines for HIV counseling, testing, and referral: critical role of and a call to action for emergency physicians. Annals of Emergency Medicine, 44(1), 31-42.

Sharland, M., Gibb, D. M., & Tudor-Williams, G. (2003). Advances in the prevention and treatment of paediatric HIV infection in the United Kingdom. Sexually Transmitted Infections, 79(1), 53-55.

Waters, L., John, L., & Nelson, M. (2007). Non-nucleoside reverse transcriptase inhibitors: a review [Review]. International Journal of Clinical Practice, 61(1), 105-118.

Zareba, K. M., Lavigne, J. E., & Lipshultz, S. E. (2004). Cardiovascular effects of HAART in infants and children of HIV-infected mothers. Cardiovascular Toxicology, 4(3), 271-279.

**Inappropriate study design: 38**

Agboghoroma CO. Gynaecological and reproductive health issues in HIV-positive women. West African Journal of Medicine. 2010;29(3):135-42.

Ahmad N. Molecular mechanisms of HIV-1 mother-to-child transmission and infection in neonatal target cells. Life Sciences. 2011;88(21-22):980-6.

Aizire J, Fowler MG, Coovadia HM. Operational issues and barriers to implementation of prevention of mother-to-child transmission of HIV (PMTCT) interventions in Sub-Saharan Africa. Current HIV Research. 2013;11(2):144-59.

Ammann AJ. Optimal versus suboptimal treatment for HIV-infected pregnant women and HIV-exposed infants in clinical research studies. Journal of Acquired Immune Deficiency Syndromes: JAIDS. 2009;51(5):509-12.

Andany N, Loutfy MR. HIV protease inhibitors in pregnancy: pharmacology and clinical use. Drugs. 2013;73(3):229-47.

Astawesegn FH, Stulz V, Conroy E, Mannan H. Trends and effects of antiretroviral therapy coverage during pregnancy on mother-to-child transmission of HIV in Sub-Saharan Africa. Evidence from panel data analysis. BMC Infectious Diseases. 2022;22(1):134.

Balkus JE, Neradilek M, Fairlie L, Makanani B, Mgodi N, Mhlanga F, et al. Assessing pregnancy and neonatal outcomes in Malawi, South Africa, Uganda, and Zimbabwe: Results from a systematic chart review. PLoS ONE. 2021;16(3):e0248423.

Bendle M, Bajpai S, Choudhary A, Pazare A. Prevention of perinatal HIV I transmission by protease inhibitor based triple drug antiretroviral therapy versus nevirapine as single dose at the time of delivery. Journal of the Association of Physicians of India. 2012; 60:39-44.

Beranger A, Bekker A, Solans BP, Cotton MF, Mirochnick M, Violari A, et al. Influence of NAT2 genotype and maturation on isoniazid exposure in low-birth-weight and preterm infants with or without HIV exposure. Clinical Infectious Diseases. 2022;04:04.

Brummel SS, Taha TE, Angelidou KN, Saidi F, Atuhaire P, Dula D, et al. Brief Report: Impact of ART on Maternal Health After Cessation of Breastfeeding. Journal of Acquired Immune Deficiency Syndromes: JAIDS. 2021;86(4):450-4.

Buchanan AM, Cunningham CK. Advances and failures in preventing perinatal human immunodeficiency virus infection. Clinical Microbiology Reviews. 2009;22(3):493-507.

Buckoreelall K, Cressey TR, King JR. Pharmacokinetic optimization of antiretroviral therapy in pregnancy. Clinical Pharmacokinetics. 2012;51(10):639-59.

Bukkems V, Necsoi C, Tenorio CH, Garcia C, Rockstroh J, Schwarze-Zander C, et al. Clinically Significant Lower Elvitegravir Exposure During the Third Trimester of Pregnant Patients Living With Human Immunodeficiency Virus: Data From the Pharmacokinetics of ANtiretroviral agents in HIV-infected pregNAnt women (PANNA) Network. Clinical Infectious Diseases. 2020;71(10):e714-e7.

Grant PM, Zolopa AR. Optimal antiretroviral therapy: HIV-1 treatment strategies to avoid and overcome drug resistance. Current Opinion in Investigational Drugs. 2010;11(8):901-10.

Hasnain M. Antenatal HIV screening and treatment in South Africa: social norms and policy options. African Journal of Reproductive Health. 2004;8(2):77-85.

Jamieson DJ, Read JS, Kourtis AP, Durant TM, Lampe MA, Dominguez KL. Cesarean delivery for HIV-infected women: recommendations and controversies. American Journal of Obstetrics & Gynecology. 2007;197(3 Suppl):S96-100.

Kalungwe M, Mbalinda SN, Karonga T, Simwanza NR, Mumba Mtambo CM, Nyashanu M. Exploring barriers to antiretroviral therapy adherence among pregnant women: A scoping literature review. International Journal of Gynaecology & Obstetrics. 2022;159(2):343-50.

Katz A. The evolving art of caring for pregnant women with HIV infection. JOGNN - Journal of Obstetric, Gynecologic, & Neonatal Nursing. 2003;32(1):102-8.

Khoo S, Peytavin G, Burger D, Hill A, Brown K, Moecklinghoff C, et al. Pharmacokinetics and Safety of Darunavir/Ritonavir in HIV-Infected Pregnant Women. AIDS Reviews. 2017;19(1):16-23.

Kidd MR, Anderson J. Antiretroviral medications and HIV. Australian Family Physician. 2002;31(2):135-9.

Kriebs JM. Changing the paradigm: HIV in pregnancy. Journal of Perinatal & Neonatal Nursing. 2006;20(1):71-3.

Martin F, Taylor GP. The safety of highly active antiretroviral therapy for the HIV-positive pregnant mother and her baby: is 'the more the merrier'? Journal of Antimicrobial Chemotherapy. 2009;64(5):895-900.

Miller N. HIV & pregnancy--what more can we do for HIV-positive women? Midwifery Today with International Midwife. 2004(71):24-9.

Minkoff H. Human immunodeficiency virus infection in pregnancy. Obstetrics & Gynecology. 2003;101(4):797-810.

Mirochnick M, Best BM, Clarke DF. Antiretroviral pharmacology: special issues regarding pregnant women and neonates. Clinics in Perinatology. 2010;37(4):907-27, xi.

Newell ML, Bunders MJ. Safety of antiretroviral drugs in pregnancy and breastfeeding for mother and child. Current Opinion in HIV & AIDS. 2013;8(5):504-10.

Newell ML, Thorne C. Antiretroviral therapy and mother-to-child transmission of HIV-1. Expert Review of Antiinfective Therapy. 2004;2(5):717-32.

Newshan G, Hoyt MJ. Use of combination antiretroviral therapy in pregnant women with HIV disease. MCN, American Journal of Maternal Child Nursing. 1998;23(6):307-12; quiz 13.

Niemiec T, Okninska A, El Midaoui A. Management and treatment of HIV infected pregnant women in Poland. Medycyna Wieku Rozwojowego. 2003;7(4 Pt 1):415-23.

Paintsil E, Andiman WA. Care and management of the infant of the HIV-1-infected mother. Seminars in Perinatology. 2007;31(2):112-23.

Rongkavilit C, Asmar BI. Advances in prevention of mother-to-child HIV transmission. Indian Journal of Pediatrics. 2004;71(1):69-79.

Rongkavilit C, Asmar BI. Advances in prevention of mother-to-child HIV transmission: the international perspectives. Indian Journal of Pediatrics. 2011;78(2):192-204.

Talaie H, Nava-Ocampo AA, Koren G. Antiretroviral treatment of maternal HIV infection. Canadian Family Physician. 2004;50:865-8.

Townsend C, Schulte J, Thorne C, Dominguez KI, Tookey PA, Cortina-Borja M, et al. Antiretroviral therapy and preterm delivery-a pooled analysis of data from the United States and Europe. BJOG: An International Journal of Obstetrics & Gynaecology. 2010;117(11):1399-410.

Townsend CL, Cortina-Borja M, Peckham CS, Tookey PA. Response to Kourtis et al. 'Use of antiretroviral therapy in pregnant HIV-infected women and the risk of premature delivery: a meta-analysis'. Aids. 2007;21(13):1831-2.

Vogler MA, Singh H, Wright R. Complex decisions in managing HIV infection during pregnancy. Current HIV/AIDS Reports. 2011;8(2):122-31.

Wunder D, Evison JM. [Antiretroviral therapy and pregnancy]. Therapeutische Umschau. 2005;62(1):37-42.

Zahedi-Spung L, Badell ML. Current Strategies to Prevent Maternal-to-Child Transmission of Human Immunodeficiency Virus. Clinics in Perinatology. 2018;45(2):325-37.

**No quantitative data: 37**

Abarzua F, Nunez F, Hubinont C, Bernard P, Yombi JC, Vandercam B. [Human immunodeficiency virus (HIV) infection in pregnancy: antiretroviral treatment (ART) and mode of delivery]. Revista Chilena de Infectologia. 2005;22(4):327-37.

Abdi F, Alimoradi Z, Alidost F. Pregnancy Outcomes and Effects of Antiretroviral Drugs in HIV-positive Pregnant Women: A Systematic Review. Future Virology. 2019;14(3):197-210.

Abrams EJ. Prevention of mother-to-child transmission of HIV--successes, controversies and critical questions. AIDS Reviews. 2004;6(3):131-43.

Abrams EJ, Myer L, Rosenfield A, El-Sadr WM. Prevention of mother-to-child transmission services as a gateway to family-based human immunodeficiency virus care and treatment in resource-limited settings: rationale and international experiences. American Journal of Obstetrics & Gynecology. 2007;197(3 Suppl):S101-6.

Adam S. HIV and pregnancy: review. Obstetrics and Gynaecology Forum. 2015;25(2):19-22.

Al-Husaini AM. Role of placenta in the vertical transmission of human immunodeficiency virus. Journal of Perinatology. 2009;29(5):331-6.

Alemu FM, Yalew AW, Fantahun M, Ashu EE. Antiretroviral Therapy and Pregnancy Outcomes in Developing Countries: A Systematic Review. International Journal of MCH & AIDS. 2015;3(1):31-43.

Anderson BL, Cu-Uvin S. Pregnancy and optimal care of HIV-infected patients. Clinical Infectious Diseases. 2009;48(4):449-55.

Anderson K, Muloiwa R, Davies MA. Long-term outcomes in perinatally HIV-infected adolescents and young adults on antiretroviral therapy: a review of South African and global literature. African Journal of AIDS Research. 2020;19(1):1-12.

Atowoju I, Dawer P, Asrani M, Panjiyar B. Impact of maternal HIV infection on perinatal outcomes: a systematic review. Special Issue: Sexually transmitted infections during pregnancy. 2024;166(1):35-43.

Azwa I, Khong SY. Human immunodeficiency virus (HIV) in pregnancy: a review of the guidelines for preventing mother-to-child transmission in Malaysia. Annals of the Academy of Medicine, Singapore. 2012;41(12):587-94.

Baggaley R, van Praag E. Antiretroviral interventions to reduce mother-to-child transmission of human immunodeficiency virus: challenges for health systems, communities and society. Bulletin of the World Health Organization. 2000;78(8):1036-44.

Bruun JN. Antiviral agents reduce HIV transmission from mother to child. Current combination therapy results in best suppression of the virus. Lakartidningen. 2003;100(44):3506-8.

Cerveny L, Murthi P, Staud F. HIV in pregnancy: Mother-to-child transmission, pharmacotherapy, and toxicity. Biochimica et Biophysica Acta - Molecular Basis of Disease. 2021;1867(10):166206.

Chougrani I, Luton D, Matheron S, Mandelbrot L, Azria E. Safety of protease inhibitors in HIV-infected pregnant women. HIV/AIDS Research and Palliative Care. 2013;5:253-62.

Cohan D. Perinatal HIV: special considerations. Topics in HIV Medicine. 2003;11(6):200-13.

Donnelly M, Davies JK. Contemporary management of human immunodeficiency virus in pregnancy. Obstetrics & Gynecology Clinics of North America. 2014;41(4):547-71.

Dragovic G, Grbovic L, Jevtovic D, Andjelic S, Vasic B, Lukic R. Safety of antiretrovirals in pregnacy. Vojnosanitetski Pregled. 2011;68(11):967-74.

Dude AM, Jones M, Wilson T. Human Immunodeficiency Virus in Pregnancy. Obstetrics & Gynecology Clinics of North America. 2023;50(2):389-99.

Eke AC, Brooks KM, Gebreyohannes RD, Sheffield JS, Dooley KE, Mirochnick M. Tenofovir alafenamide use in pregnant and lactating women living with HIV. Expert Opinion On Drug Metabolism & Toxicology. 2020;16(4):333-42.

Harris K, Yudin MH. HIV Infection in Pregnant Women: A 2020 Update. Prenatal Diagnosis. 2020;40(13):1715-21.

Kotler DP. Human immunodeficiency virus and pregnancy. Gastroenterology Clinics of North America. 2003;32(1):437-48, ix.

Mofenson LM, Baggaley RC, Mameletzis I. Tenofovir disoproxil fumarate safety for women and their infants during pregnancy and breastfeeding. Aids. 2017;31(2):213-32.

Poliektov NE, Badell ML. Antiretroviral Options and Treatment Decisions During Pregnancy. Paediatric Drugs. 2023;25(3):267-82.

Premkumar A, Dude AM, Haddad LB, Yee LM. Combined antiretroviral therapy for HIV and the risk of hypertensive disorders of pregnancy: A systematic review. Pregnancy Hypertension. 2019;17:178-90.

Reshi P, Lone IM. Human immunodeficiency virus and pregnancy. Archives of Gynecology & Obstetrics. 2010;281(5):781-92.

Rigopoulos D, Gregoriou S, Paparizos V, Katsambas A. AIDS in pregnancy, part II: Treatment in the era of highly active antiretroviral therapy and management of obstetric, anesthetic, and pediatric issues. SKINmed. 2007;6(2):79-84.

Rigopoulos D, Gregoriou S, Paparizos V, Katsambas A. AIDS in pregnancy, part I: epidemiology, testing, effect on disease progression, opportunistic infections, and risk of vertical transmission. SKINmed. 2007;6(1):18-23.

Rubinstein A. Advances in antiretroviral, immune-based, and gene therapy for HIV infection in mothers and infants. Implications for future use in developing countries. Annals of the New York Academy of Sciences. 2000;918:27-35.

Saleska JL, Turner AN, Maierhofer C, Clark J, Kwiek JJ. Use of Antiretroviral Therapy During Pregnancy and Adverse Birth Outcomes Among Women Living With HIV-1 in Low- and Middle-Income Countries: A Systematic Review. Journal of Acquired Immune Deficiency Syndromes: JAIDS. 2018;79(1):1-9.

Sandelowski M, Barroso J. Motherhood in the context of maternal HIV infection. Research in Nursing & Health. 2003;26(6):470-82.

Semprini AE, Fiore S. HIV and pregnancy: is the outlook for mother and baby transformed? Current Opinion in Obstetrics & Gynecology. 2004;16(6):471-5.

Senise J, Bonafe S, Castelo A. The management of HIV-infected pregnant women. Current Opinion in Obstetrics & Gynecology. 2012;24(6):395-401.

Shannon M. Antiretroviral therapy in HIV-infected pregnant women and their infants: current interventions and challenges. Journal of Perinatal & Neonatal Nursing. 2002;16(2):1-25.

Shetty AK, Maldonado Y. Antiretroviral drugs to prevent mother-to-child transmission of HIV during breastfeeding. Current HIV Research. 2013;11(2):102-25.

Taylor GP, Lyall EG, Mercey D, Smith R, Chester T, Newell ML, et al. British HIV Association guidelines for prescribing antiretroviral therapy in pregnancy (1998). Sexually Transmitted Infections. 1999;75(2):90-7.

Van de Perre P. HIV and AIDS in Africa: impact on mother and child health. European Journal of Medical Research. 1999;4(8):341-4.

**Not relevant to the review: 15**

Brocklehurst P, Volmink J. Antiretrovirals for reducing the risk of mother-to-child transmission of HIV infection. Cochrane Database of Systematic Reviews. 2002(1):CD003510.

Eke AC, Ramaiyer M, Eleje GU, Ezebialu IU, Aliyu MH. A systematic review and meta-analysis of maternal weight changes and pregnancy outcomes associated with integrase inhibitors and tenofovir alafenamide in pregnant women with HIV. American journal of obstetrics & gynecology MFM. 2024;6(8):101406.

Huang X, Xu Y, Yang Q, Chen J, Zhang T, Li Z, et al. Efficacy and biological safety of lopinavir/ritonavir based anti-retroviral therapy in HIV-1-infected patients: a meta-analysis of randomized controlled trials. Scientific Reports. 2015;5:8528.

Kongnyuy EJ, Wiysonge CS, Shey MS. A systematic review of randomized controlled trials of prenatal and postnatal vitamin A supplementation of HIV-infected women. International Journal of Gynaecology & Obstetrics. 2009;104(1):5-8.

Kourtis AP, Schmid CH, Jamieson DJ, Lau J. Use of antiretroviral therapy in pregnant HIV-infected women and the risk of premature delivery: a meta-analysis. Aids. 2007;21(5):607-15.

Li H, Qi F, Chen D. Does the risk of neurodevelopment disorders in children differ with different ART treatments? A systematic review and meta-analysis. *Pakistan Journal of Medical Sciences* 2025; **41(8)**: 2415-24.

Mehrabi F, Karamouzian M, Farhoudi B, Moradi Falah Langeroodi S, Mehmandoost S, Abbaszadeh S, et al. Comparison of safety and effectiveness of antiretroviral therapy regimens among pregnant women living with HIV at preconception or during pregnancy: a systematic review and network meta-analysis of randomized trials. BMC Infectious Diseases. 2024;24(1):417.

Mesfin YM, Kibret KT, Taye A. Is protease inhibitors based antiretroviral therapy during pregnancy associated with an increased risk of preterm birth? Systematic review and a meta-analysis. Reproductive Health. 2016;13:30.

Mehrabi F, Karamouzian M, Farhoudi B, Moradi Falah Langeroodi S, Mehmandoost S, Abbaszadeh S, et al. Comparison of safety and effectiveness of antiretroviral therapy regimens among pregnant women living with HIV at preconception or during pregnancy: a systematic review and network meta-analysis of randomized trials. BMC Infect Dis. 2024;24(1):417.

Pasley MV, Martinez M, Hermes A, d'Amico R, Nilius A. Safety and efficacy of lopinavir/ritonavir during pregnancy: a systematic review. AIDS Rev. 2013;15(1):38-48.

Rebnord T, Lie RT, Daltveit AK, Sandoy IF. Maternal antiretroviral treatment for HIV infection and risk of small-for-gestational-age birth: A systematic review and meta-analysis of protease inhibitor-based treatment and timing of treatment. International Journal of Antimicrobial Agents. 2023;62(1):106823.

Saint-Lary L, Benevent J, Damase-Michel C, Vayssiere C, Leroy V, Sommet A. Systematic review and meta-analysis of adverse perinatal outcomes associated with prenatal exposure to protease inhibitor combination in pregnant women with HIV infection. Fundamental and Clinical Pharmacology. 2019;33(Supplement 1):11.

Saint-Lary L, Benevent J, Damase-Michel C, Vayssiere C, Leroy V, Sommet A. Adverse perinatal outcomes associated with prenatal exposure to protease-inhibitor-based versus non-nucleoside reverse transcriptase inhibitor-based antiretroviral combinations in pregnant women with HIV infection: a systematic review and meta-analysis. BMC Pregnancy & Childbirth. 2023;23(1):80.

Suksomboon N, Poolsup N, Ket-Aim S. Systematic review of the efficacy of antiretroviral therapies for reducing the risk of mother-to-child transmission of HIV infection. Journal of Clinical Pharmacy & Therapeutics. 2007;32(3):293-311.

Xiao PL, Zhou YB, Chen Y, Yang MX, Song XX, Shi Y, et al. Association between maternal HIV infection and low birth weight and prematurity: a meta-analysis of cohort studies. BMC Pregnancy & Childbirth. 2015;15:246.

**Outcome of interest not defined correctly: 6**

Adelekan B, Harry-Erin B, Okposo M, Aliyu A, Ndembi N, Dakum P, et al. Final HIV status outcome for HIV-exposed infants at 18 months of age in nine states and the Federal Capital Territory, Nigeria. PLoS ONE [Electronic Resource]. 2022;17(2):e0263921.

Ahmed EH, Shafei MA, Alsubhi LS, Zarban NA, Al-Zahrani BA, Shaikhomer M. The outcome of prevention of mother-to-child transmission of HIV infection at King Abdulaziz University Hospital, Western Region of Saudi Arabia: A Descriptive Study. Saudi Med J. 2021;42(9):1009-16.

Aizire J, Sikorskii A, Ogwang LW, Kawalazira R, Mutebe A, Familiar-Lopez I, et al. Decreased growth among antiretroviral drug and HIV-exposed uninfected versus unexposed children in Malawi and Uganda. Aids. 2020;34(2):215-25.

Baijal N, Seth A, Singh S, Sharma G, Kumar P, Chandra J. HIV-free Survival at the Age of 18 Months in Children Born to Women With HIV Infection: A Retrospective Cohort Study. Indian Pediatr. 2020;57(1):34-8.

Brocklehurst P, French R. The association between maternal HIV infection and perinatal outcome: a systematic review of the literature and meta-analysis. British Journal of Obstetrics & Gynaecology. 1998;105(8):836-48.

Veroniki AA, Antony J, Straus SE, Ashoor HM, Finkelstein Y, Khan PA, et al. Comparative safety and effectiveness of perinatal antiretroviral therapies for HIV-infected women and their children: Systematic review and network meta-analysis including different study designs. PLoS ONE [Electronic Resource]. 2018;13(6):e0198447.

**Outcome of interest not measured: 71**

Aaron KJ, Brill I, Causey-Pruitt Z, Murphy K, Augenbraun M, Kassaye S, et al. Factors associated with syphilis seroprevalence in women with and at-risk for HIV infection in the Women's Interagency HIV Study (1994-2015). Sexually Transmitted Infections. 2022;98(1):4-10.

Abate HK, Mekonnen CK, Ferede YM. Depression Among HIV-Positive Pregnant Women at Northwest Amhara Referral Hospitals During COVID-19 Pandemic. Risk manag. 2021;14:4897-905.

Abdisa S, Tenaw Z. Level of adherence to option B plus PMTCT and associated factors among HIV positive pregnant and lactating women in public health facilities of Hawassa city, Southern Ethiopia. PLoS ONE. 2021;16(8):e0255808.

Abebe W, Gebremariam M, Molla M, Teferra S, Wissow L, Ruff A. Prevalence of depression among HIV-positive pregnant women and its association with adherence to antiretroviral therapy in Addis Ababa, Ethiopia. PLoS ONE. 2022;17(1):e0262638.

Abraham SA, Clow SE. Staying, leaving and returning: Trends of prevention of mother-to-child transmission retention among newly diagnosed HIV-positive pregnant and postpartum women. Int J STD AIDS. 2022;33(1):81-7.

Adams JW, Watts DH, Phelps BR. A systematic review of the effect of HIV infection and antiretroviral therapy on the risk of pre-eclampsia. International Journal of Gynecology & Obstetrics. 2016;133(1):17-21.

Adeniyi OV, Ajayi AI. Level and determinants of postpartum adherence to antiretroviral therapy in the Eastern Cape, South Africa. PLoS ONE. 2020;15(2):e0229592.

Afonina L, Voronin EE. [Use of darunavir in HIV-infected women during pregnancy]. Terapevticheskii Arkhiv. 2013;85(11):109-14.

Agabu A, Baughman AL, Fischer-Walker C, de Klerk M, Mutenda N, Rusberg F, et al. National-level effectiveness of ART to prevent early mother to child transmission of HIV in Namibia. PLoS ONE [Electronic Resource]. 2020;15(11):e0233341.

Ahoua L, Arikawa S, Tiendrebeogo T, Lahuerta M, Aly D, Becquet R, et al. Measuring retention in care for HIV-positive pregnant women in Prevention of Mother-to-Child Transmission of HIV (PMTCT) option B+ programs: the Mozambique experience. BMC Public Health. 2020;20(1):322.

Akoto C, Chan CYS, Ravi K, Zhang W, Vatish M, Norris SA, et al. gammadelta T cell frequencies are altered in HIV positive pregnant South African women and are associated with preterm birth. PLoS ONE. 2020;15(6):e0235162.

Akoto C, Chan CYS, Tshivuila-Matala COO, Ravi K, Zhang W, Vatish M, et al. Innate lymphoid cells are reduced in pregnant HIV positive women and are associated with preterm birth. Scientific Reports. 2020;10(1):13265.

Akoto C, Norris SA, Hemelaar J. Maternal HIV infection is associated with distinct systemic cytokine profiles throughout pregnancy in South African women. Scientific Reports. 2021;11(1):10079.

Alonso S, Vidal M, Ruiz-Olalla G, Gonzalez R, Manaca MN, Jairoce C, et al. Reduced Placental Transfer of Antibodies Against a Wide Range of Microbial and Vaccine Antigens in HIV-Infected Women in Mozambique. Frontiers in Immunology. 2021;12:614246.

Andiman W, Bryson Y, de Martino M, Fowler M, Harris D, Hutto C, et al. The mode of delivery and the risk of vertical transmission of human immunodeficiency virus type 1--a meta-analysis of 15 prospective cohort studies. New England Journal of Medicine. 1999;340(13):977-87.

Anonymous. Nucleoside exposure in the children of HIV-infected women receiving antiretroviral drugs: absence of clear evidence for mitochondrial disease in children who died before 5 years of age in five United States cohorts. Journal of Acquired Immune Deficiency Syndromes: JAIDS. 2000;25(3):261-8.

Arikawa S, Rollins N, Newell ML, Becquet R. Mortality risk and associated factors in HIV-exposed, uninfected children. Tropical Medicine & International Health. 2016;21(6):720-34.

Astawesegn FH, Mannan H, Stulz V, Conroy E. Understanding the uptake and determinants of prevention of mother-to-child transmission of HIV services in East Africa: mixed methods systematic review and meta-analysis. PLoS ONE. 2024;19(4).

Astawesegn FH, Stulz V, Agho KE, Mannan H, Conroy E, Ogbo FA. Prenatal HIV Test Uptake and Its Associated Factors for Prevention of Mother to Child Transmission of HIV in East Africa. Int J Environ Res Public Health. 2021;18(10):16.

Atuhaire P, Matovu F, Nakalega R, Kataike H, Nabwana M, Lukyamuzi Z, et al. Time to first viral load testing among pregnant women living with HIV initiated on option B+ at 5 government clinics in Kampala city, Uganda: Retrospective cohort study. Int J Infect Dis. 2021;104:526-31.

Awadu JE, Sikorskii A, Zalwango S, Coventry A, Giordani B, Ezeamama AE. Developmental Disorder Probability Scores at 6-18 Years Old in Relation to In-Utero/Peripartum Antiretroviral Drug Exposure among Ugandan Children. Int J Environ Res Public Health. 2022;19(6):21.

Bebell LM, Parks K, Le MH, Ngonzi J, Adong J, Boatin AA, et al. Placental Decidual Arteriopathy and Vascular Endothelial Growth Factor A Expression Among Women With or Without Human Immunodeficiency Virus. Journal of Infectious Diseases. 2021;224(12 Suppl 2):S694-S700.

Becquet R, Marston M, Dabis F, Moulton LH, Gray G, Coovadia HM, et al. Children who acquire HIV infection perinatally are at higher risk of early death than those acquiring infection through breastmilk: a meta-analysis. PLoS ONE [Electronic Resource]. 2012;7(2):e28510.

Bera E, Mia R. Safety of nevirapine in HIV-infected pregnant women initiating antiretroviral therapy at higher CD4 counts: a systematic review and meta-analysis. South African Medical Journal Suid-Afrikaanse Tydskrif Vir Geneeskunde. 2012;102(11 Pt 1):855-9.

Browne JL, Schrier VJMM, Grobbee DE, Peters SAE, Klipstein-Grobusch K. HIV, antiretroviral therapy, and hypertensive disorders in pregnancy: a systematic review and meta-analysis. JAIDS, Journal of Acquired Immune Deficiency Syndromes. 2015;70(1):91-8.

Burrage AB, Mushavi A, Shiraishi RW, Barr BT, Shambira G, Nyakura J, et al. Mother-To-Child Transmission of HIV in Adolescents and Young Women: Findings From a National Prospective Cohort Survey, Zimbabwe, 2013-2014. J Adolesc Health. 2020;66(4):455-63.

Calvert C, Ronsmans C. HIV and the risk of direct obstetric complications: a systematic review and meta-analysis. PLoS ONE [Electronic Resource]. 2013;8(10):e74848.

Calvert C, Ronsmans C. The contribution of HIV to pregnancy-related mortality: a systematic review and meta-analysis. Aids. 2013;27(10):1631-9.

Calvert C, Ronsmans C. Pregnancy and HIV disease progression: a systematic review and meta-analysis. Tropical Medicine & International Health. 2015;20(2):122-45.

Chi BH, Stringer JS, Moodley D. Antiretroviral drug regimens to prevent mother-to-child transmission of HIV: a review of scientific, program, and policy advances for sub-Saharan Africa. Current HIV/AIDS Reports. 2013;10(2):124-33.

Danso-Appiah A, Akuffo KO, Owiredu D. Mental health problems in pregnant and postpartum women living with HIV in sub- Saharan Africa: Systematic review and metaanalysis protocol. PLoS ONE. 2024;19(10 October):e0308810.

Dunk CE, Serghides L. Protease inhibitor-based antiretroviral therapy in pregnancy: effects on hormones, placenta, and decidua. The Lancet HIV. 2022;9(2):e120-e9.

Foster EG, Gendelman HE, Bade AN. HIV-1 Integrase Strand Transfer Inhibitors and Neurodevelopment. Pharmaceuticals. 2022;15(12):09.

French R, Brocklehurst P. The effect of pregnancy on survival in women infected with HIV: a systematic review of the literature and meta-analysis. British Journal of Obstetrics & Gynaecology. 1998;105(8):827-35.

Ge K, Liu X, Ruan W, Wu X, Zhang Z. Interventions to prevent vertical transmission of HIV: an umbrella review of systematic reviews and meta-analyses. AIDS Reviews 2025; 27(3): 104-15.

Hill A, Clayden P, Thorne C, Christie R, Zash R. Safety and pharmacokinetics of dolutegravir in HIV-positive pregnant women: a systematic review. Journal of Virus Eradication. 2018;4(2):66-71.

Kanters S, Vitoria M, Zoratti M, Doherty M, Penazzato M, Rangaraj A, et al. Comparative efficacy, tolerability and safety of dolutegravir and efavirenz 400mg among antiretroviral therapies for first-line HIV treatment: A systematic literature review and network meta-analysis. EClinicalMedicine. 2020;28:100573.

Jasper A, Fourie W, Chetty S. The safety of cabotegravir in pregnancy: a systematic review and meta-analysis. BMC Infectious Diseases 2025; 25(1): 1550.

Maingi M, Stark AH, Iron-Segev S. The impact of Option B+ on mother-to-child transmission of HIV in Africa: A systematic review. Tropical Medicine & International Health. 2022;27(6):553-63.

Mataramvura H, Bunders MJ, Duri K. Human immunodeficiency virus and antiretroviral therapy-mediated immune cell metabolic dysregulation in children born to HIV-infected women: potential clinical implications. Frontiers in Immunology. 2023;14:1182217.

McHenry MS, Balogun KA, McDonald BC, Vreeman RC, Whipple EC, Serghides L. In utero exposure to HIV and/or antiretroviral therapy: a systematic review of preclinical and clinical evidence of cognitive outcomes. Journal of the International AIDS Society. 2019;22(4):e25275.

McIntyre J. Use of antiretrovirals during pregnancy and breastfeeding in low-income and middle-income countries. Current Opinion in HIV & AIDS. 2010;5(1):48-53.

Mirochnick M, Capparelli E. Pharmacokinetics of antiretrovirals in pregnant women. Clinical Pharmacokinetics. 2004;43(15):1071-87.

Moodley J, Wennberg JL. HIV in pregnancy. Current Opinion in Obstetrics & Gynecology. 2005;17(2):117-21.

Nachega JB, Uthman OA, Anderson J, Peltzer K, Wampold S, Cotton MF, et al. Adherence to antiretroviral therapy during and after pregnancy in low-income, middle-income, and high-income countries: a systematic review and meta-analysis. Aids. 2012;26(16):2039-52.

Ndlovu KS, Pavan RR, Corry J, et al. The vaginal microbiome of pregnant people living with HIV on antiretroviral therapy in the Democratic Republic of Congo: a pilot study and global meta-analysis. *Msphere* 2026: e0059725.

Neary M, Owen A, Olagunju A. Pharmacokinetics of HIV therapies in pregnant patients: an update. Expert Opinion On Drug Metabolism & Toxicology. 2020;16(6):449-61.

Nguyen B, Foisy MM, Hughes CA. Pharmacokinetics and Safety of the Integrase Inhibitors Elvitegravir and Dolutegravir in Pregnant Women With HIV. Annals of Pharmacotherapy. 2019;53(8):833-44.

Nourollahpour Shiadeh M, Riahi SM, Khani S, Alizadeh S, Hosseinzadeh R, Hasanpour AH, et al. Human Immunodeficiency Virus and risk of pre-eclampsia and eclampsia in pregnant women: A meta-analysis on cohort studies. Pregnancy Hypertension. 2019;17:269-75.

Omonaiye O, Kusljic S, Nicholson P, Manias E. Medication adherence in pregnant women with human immunodeficiency virus receiving antiretroviral therapy in sub-Saharan Africa: a systematic review. BMC Public Health. 2018;18(1):805.

Ortiz-Ibarra FJ. [HIV/AIDS in pregnancy and lactation]. Gaceta Medica de Mexico. 2006;142 Suppl 2:61-8.

Paredes R, Marconi VC, Lockman S, Abrams EJ, Kuhn L. Impact of antiretroviral drugs in pregnant women and their children in Africa: HIV resistance and treatment outcomes. Journal of Infectious Diseases. 2013;207 Suppl 2:S93-100.

Peiperl L. Antiretroviral treatments to reduce mother-to-child transmission of HIV. HIV Clinical Trials. 2001;2(1):46-55.

Petropoulou H, Stratigos AJ, Katsambas AD. Human immunodeficiency virus infection and pregnancy. Clinics in Dermatology. 2006;24(6):536-42.

Pope R, Jr., Kashuba A. Darunavir for use in pregnant women with HIV. Expert Review of Clinical Pharmacology. 2017;10(12):1317-27.

Psaros C, Remmert JE, Bangsberg DR, Safren SA, Smit JA. Adherence to HIV care after pregnancy among women in sub-Saharan Africa: falling off the cliff of the treatment cascade. Current HIV/AIDS Reports. 2015;12(1):1-5.

Rakhmanina NY, van den Anker JN, Soldin SJ. Safety and pharmacokinetics of antiretroviral therapy during pregnancy. Therapeutic Drug Monitoring. 2004;26(2):110-5.

Rosen JE, de Zoysa I, Dehne K, Mangiaterra V, Abdool-Karim Q. Understanding methods for estimating HIV-associated maternal mortality. Journal of Pregnancy. 2012;2012:958262.

Roustit M, Jlaiel M, Leclercq P, Stanke-Labesque F. Pharmacokinetics and therapeutic drug monitoring of antiretrovirals in pregnant women. British Journal of Clinical Pharmacology. 2008;66(2):179-95.

Short CE, Taylor GP. Antiretroviral therapy and preterm birth in HIV-infected women. Expert Review of Antiinfective Therapy. 2014;12(3):293-306.

Siegfried N, van der Merwe L, Brocklehurst P, Sint TT. Antiretrovirals for reducing the risk of mother-to-child transmission of HIV infection. Cochrane Database of Systematic Reviews. 2011(7):CD003510.

Stalter RM, Pintye J, Mugwanya KK. Safety review of tenofovir disoproxil fumarate/emtricitabine pre-exposure prophylaxis for pregnant women at risk of HIV infection. Expert Opinion on Drug Safety. 2021;20(11):1367-73.

Stek AM. Antiretroviral medications during pregnancy for therapy or prophylaxis. Current HIV/AIDS Reports. 2009;6(2):68-76.

Suthar AB, Hoos D, Beqiri A, Lorenz-Dehne K, McClure C, Duncombe C. Integrating antiretroviral therapy into antenatal care and maternal and child health settings: a systematic review and meta-analysis. Bulletin of the World Health Organization. 2013;91(1):46-56.

Taylor GP, Low-Beer N. Antiretroviral therapy in pregnancy: a focus on safety. Drug Safety. 2001;24(9):683-702.

Thorne C, Newell ML. Antenatal and neonatal antiretroviral therapy in HIV-infected women and their infants: a review of safety issues. Medycyna Wieku Rozwojowego. 2003;7(4 Pt 1):425-36.

Thorne C, Newell ML. The safety of antiretroviral drugs in pregnancy. Expert Opinion on Drug Safety. 2005;4(2):323-35.

Volmink J, Siegfried NL, van der Merwe L, Brocklehurst P. Antiretrovirals for reducing the risk of mother-to-child transmission of HIV infection. Cochrane Database of Systematic Reviews. 2007(1):CD003510.

Watts DH. Maternal therapy for HIV in pregnancy. Clinical Obstetrics & Gynecology. 2001;44(2):182-97.

White AB, Mirjahangir JF, Horvath H, Anglemyer A, Read JS. Antiretroviral interventions for preventing breast milk transmission of HIV. Cochrane Database of Systematic Reviews. 2014(10):CD011323.

Zhou H, Liu L, Zhang M, Chen X, Huang Z. Antiretroviral therapy among pregnant and postpartum women in China: A systematic review and meta-analysis. American Journal of Infection Control. 2016;44(3):e25-35.

## Appendix 12.2 Excluded studies: randomised controlled trials

**Inappropriate population: 12**

Aboulker JP, Babiker A, Chaix ML, Compagnucci A, Darbyshire J, Debre M, et al. Highly active antiretroviral therapy started in infants under 3 months of age: 72-week follow-up for CD4 cell count, viral load and drug resistance outcome. AIDS. 2004;18(2):237-45.

Augusto O, Stergachis A, Dellicour S, Tinto H, Vala A, Ruperez M, et al. First trimester use of artemisinin-based combination therapy and the risk of low birth weight and small for gestational age. Malaria Journal. 2020;19(144).

Bekker A, Yang J, Wang J, Cotton MF, Cababasay M, Wiesner L, et al. Safety and Pharmacokinetics of Lopinavir/Ritonavir Oral Solution in Preterm and Term Infants Starting Before 3 Months of Age. Pediatr Infect Dis J. 2024;43(4):355-60.

Bhattacharya D, Guo R, Tseng CH, Emel L, Sun R, Chiu SH, et al. Maternal HBV Viremia and Association With Adverse Infant Outcomes in Women Living With HIV and HBV. The Pediatric infectious disease journal. 2020;11.

Bunge K, Balkus JE, Fairlie L, Mayo AJ, Nakabiito C, Mgodi N, et al. DELIVER: A Safety Study of a Dapivirine Vaginal Ring and Oral PrEP for the Prevention of HIV during Pregnancy. Journal of Acquired Immune Deficiency Syndromes. 2024;95(1):65

EP - 73.

Delany-Moretlwe S, Hanscom B, Guo X, et al. Evaluation of long-acting cabotegravir safety and pharmacokinetics in pregnant women in eastern and southern Africa: a secondary analysis of HPTN 084. Journal of the International AIDS Society 2025; 28(1): e26401.

Denoeud-Ndam L, Zannou DM, Fourcade C, Taron-Brocard C, Porcher R, Atadokpede F, et al. Cotrimoxazole prophylaxis versus mefloquine intermittent preventive treatment to prevent malaria in HIV-infected pregnant women: two randomized controlled trials. Journal of Acquired Immune Deficiency Syndromes: JAIDS. 2014;65(2):198-206.

Fairlie L, Szydlo DW, Mayo A, et al. Safety outcomes among infants whose mothers used the dapivirine vaginal ring or oral PrEP during pregnancy (MTN-042/DELIVER): a randomised phase 3b study. The Lancet HIV 2025; 12(11): e763-e73.

Faye A, Compagnucci A. Highly active antiretroviral therapy started in infants under 3 months of age: 72-Week follow-up for CD4 cell count, viral load and drug resistance outcome. Aids. 2004;18(2):237-45.

Gichangi PB, Ndinya-Achola JO, Ombete J, Nagelkerke NJ, Temmerman M. Antimicrobial prophylaxis in pregnancy: a randomized, placebo-controlled trial with cefetamet-pivoxil in pregnant women with a poor obstetric history. American journal of obstetrics and gynecology. 1997;177(3):680-4.

Girard PM, Antinori A, Arribas JR, Ripamonti D, Bicer C, Netzle-Sveine B, et al. Week 96 efficacy and safety of darunavir/ritonavir monotherapy vs. darunavir/ritonavir with two nucleoside reverse transcriptase inhibitors in the PROTEA trial. HIV Medicine. 2017;18(1):5-12.

Heffron R, Mugo N, Hong T, Celum C, Marzinke MA, Ngure K, et al. Pregnancy outcomes and infant growth among babies with in-utero exposure to tenofovir-based preexposure prophylaxis for HIV prevention. AIDS. 2018;32(12):1707-13.

**Inappropriate study design: 10**

Andany N, Loutfy M. HIV Protease Inhibitors in Pregnancy. Drugs. 2013;73(3):229-47.

Asif S, Baxevanidi E, Hill A, Venter WDF, Fairlie L, Masenya M, et al. The predicted risk of adverse pregnancy outcomes as a result of treatment-associated obesity in a hypothetical population receiving tenofovir alafenamide/emtricitabine/dolutegravir, tenofovir disoproxil fumarate/emtricitabine/dolutegravir or tenofovir disoproxil fumarate/emtricitabine/efavirenz. AIDS. 2021;35(Suppl 2):S117-S25.

Bailey AJ, Newell ML, Peckham CS. Is zidovudine therapy in pregnant HIV-infected women associated with gestational age and birthweight? Aids. 1999;13(1):119-24.

Bekker A, Capparelli EV, Mirochnick M, Clarke DF, Cotton MF, Shapiro R, et al. Lamivudine dosing for preterm infants exposed to HIV: a population pharmacokinetic modelling and simulation study. Journal of Antimicrobial Chemotherapy. 2024;79(10):2570

EP - 4.

Caniglia EC, Zash R, Diseko M, et al. Target trials of preconception switch from nevirapine or efavirenz-based antiretroviral therapy to dolutegravir-based antiretroviral therapy on adverse birth and maternal outcomes. AIDS 2025; 39(14): 2103-13.

Huang X, Xu Y, Yang Q, Chen J, Zhang T, Li Z, et al. Efficacy and biological safety of lopinavir/ritonavir based anti-retroviral therapy in HIV-1-infected patients: a meta-analysis of randomized controlled trials. Scientific Reports. 2015;5:8528.

Taha TE, Justesen A, Paterson K, Mtimavalye LA, Munthali P, Canner JK, et al. An intervention to reduce the risk of mother-to-infant HIV transmission: results of a pilot toxicity study. East Afr Med J. 1994;71(11):712-5.

Traisathit P, Le Coeur S, Mary JY, Kanjanasing A, Lamlertkittikul S, Lallemant M. Gestational age determination and prevention of HIV perinatal transmission. International Journal of Gynaecology & Obstetrics. 2006;92(2):176-80.

White A, Andrews E, Eldridge R, Dickerson M, Tilson H, Elkins M, et al. Birth outcomes following zidovudine therapy in pregnant women. JAMA. 1994;272(1):17.

Yoder DK. Zidovudine for the reduction of perinatal transmission of human immunodeficiency virus. Journal of the Tennessee Medical Association. 1995;88(7):272

EP - 4.

**Outcome not defined appropriately: 4**

Dabis F, Bequet L, Ekouevi DK, Viho I, Rouet F, Horo A, et al. Field efficacy of zidovudine, lamivudine and single-dose nevirapine to prevent peripartum HIV transmission. AIDS. 2005;19(3):309-18.

Masaba R, Borkowf CB, Girde S, Zeh C, Ndivo R, Nyang'au I, et al. Adverse fetal and infant outcomes among HIV-infected women who received either nonnucleoside reverse transcriptase inhibitor-based or protease inhibitor-based antiretroviral therapy for prevention of mother-to-child transmission. AIDS. 2018;32(12):1625-32.

Omer SB. Twelve-month follow-up of Six Week Extended Dose Nevirapine randomized controlled trials: differential impact of extended-dose nevirapine on mother-to-child transmission and infant death by maternal CD4 cell count. AIDS. 2011;25(6):767-76.

Sebikari D, Farhad M, Fenton T, Owor M, Stringer JSA, Qin M, et al. Risk factors for adverse birth outcomes in the PROMISE 1077BF/1077FF trial. JAIDS, Journal of Acquired Immune Deficiency Syndromes. 2019;81(5):521-32.

**Outcome not measured: 25**

Amone A, Gabagaya G, Wavamunno P, Rukundo G, Namale-Matovu J, Malamba SS, et al. Enhanced peer-group strategies to support the prevention of mother-to-child HIV transmission leads to increased retention in care in Uganda: A randomized controlled trial. PLoS One. 2024;19(4):e0297652.

Aziz N, Sokoloff A, Kornak J, Leva NV, Mendiola ML, Levison J, et al. Time to viral load suppression in antiretroviral-naive and -experienced HIV-infected pregnant women on highly active antiretroviral therapy: implications for pregnant women presenting late in gestation. BJOG: An International Journal of Obstetrics & Gynaecology. 2013;120(12):1534-47.

Bae WH, Wester C, Smeaton LM, Shapiro RL, Lockman S, Onyait K, et al. Hematologic and hepatic toxicities associated with antenatal and postnatal exposure to maternal highly active antiretroviral therapy among infants. AIDS. 2008;22(13):1633-40.

Balsley J. Efficacy of zidovudine in preventing HIV transmission from mother to infant. Am J Med. 1997;102(5b):45-6.

Baltrusaitis K, Makanani B, Tierney C, Fowler MG, Moodley D, Theron G, et al. Maternal and infant renal safety following tenofovir disoproxil fumarate exposure during pregnancy in a randomized control trial. BMC Infectious Diseases. 2022;22(634).

Biggar RJ, Miotti PG, Taha TE, Mtimavalye L, Broadhead R, Justesen A, et al. Perinatal intervention trial in Africa: Effect of a birth canal cleansing intervention to prevent HIV transmission. Lancet. 1996;347(9016):1647

EP - 50.

Bishop MD, Korutaro V, Boyce CL, Beck IA, Styrchak SM, Knowles K, et al. Characterizing HIV drug resistance in cases of vertical transmission in the VESTED randomized antiretroviral treatment trial. Journal of acquired immune deficiency syndromes (1999). 2024;96(4):385-92.

Caniglia EC, Zash R, Diseko M, Mayondi G, Mabuta J, Mmalane M, et al. How much could anemia-related interventions reduce the HIV disparity in adverse birth outcomes? Am J Epidemiol. 2024.

Chi BH, Saidi F, Graybill LA, Phanga T, Mollan KR, Amico KR, et al. A Patient-Centered, Combination Intervention to Support Adherence to HIV Pre-exposure Prophylaxis During Pregnancy and Breastfeeding: A Randomized Pilot Study in Malawi. J Acquir Immune Defic Syndr. 2024;95(1):42-51.

Cohan D, Natureeba P, Koss CA, Plenty A, Luwedde F, Mwesigwa J, et al. Efficacy and safety of lopinavir/ritonavir versus efavirenz-based antiretroviral therapy in HIV-infected pregnant Ugandan women. AIDS. 2015;29(2):183-91.

Colvin M, Chopra M, Doherty T, Jackson D, Levin J, Willumsen J, et al. Operational effectiveness of single-dose nevirapine in preventing mother-to-child transmission of HIV. Bulletin of the World Health Organization. 2007;85(6):466-73.

Cressey TR, Jourdain G, Lallemant MJ, Kunkeaw S, Jackson JB, Musoke P, et al. Persistence of nevirapine exposure during the postpartum period after intrapartum single-dose nevirapine in addition to zidovudine prophylaxis for the prevention of mother-to-child transmission of HIV-1. Journal of Acquired Immune Deficiency Syndromes: JAIDS. 2005;38(3):283-8.

Dadabhai S, Chou VB, Pinilla M, Chinula L, Owor M, Violari A, et al. Effects of preterm birth, maternal ART and breastfeeding on 24-month infant HIV-free survival in a randomized trial. AIDS (London, England). 2024;38(9):1304-13.

Dollfus C, Le Chenadec J, Mandelbrot L, Tubiana R, Faye A, Brossard M, et al. Improved Hematologic Outcomes in HIV1-Exposed Infants Receiving Nevirapine Compared With Zidovudine for Postnatal Prophylaxis in a High Resource Setting. Pediatric Infectious Disease Journal. 2022;41(5):420-3.

Eke AC, McCormack SA, Best BM, Stek AM, Wang J, Kreitchmann R, et al. Pharmacokinetics of Increased Nelfinavir Plasma Concentrations in Women During Pregnancy and Postpartum. Journal of Clinical Pharmacology. 2019;59(3):386-93.

Eke AC, Stek AM, Wang J, Kreitchmann R, Shapiro DE, Smith E, et al. Darunavir Pharmacokinetics With an Increased Dose During Pregnancy. Journal of Acquired Immune Deficiency Syndromes: JAIDS. 2020;83(4):373-80.

Eke AC, Brummel SS, Aliyu MH, et al. Lipid and Glucose Profiles in Pregnant Women With HIV on Tenofovir-based Antiretroviral Therapy. Clinical Infectious Diseases 2025; 80(3): 594-601.

Kovacs A, Cowles MK, Britto P, Capparelli E, Fowler MG, Moye J, et al. Pharmacokinetics of didanosine and drug resistance mutations in infants exposed to zidovudine during gestation or postnatally and treated with didanosine or zidovudine in the first three months of life. Pediatric Infectious Disease Journal. 2005;24(6):503-9.

Morris K. Short course of AZT halves HIV-1 perinatal transmission. Lancet (London, England). 1998;351(9103):651.

Musoke P, Guay LA, Bagenda D, Mirochnick M, Nakabiito C, Fleming T, et al. A phase I/II study of the safety and pharmacokinetics of nevirapine in HIV-1-infected pregnant Ugandan women and their neonates (HIVNET 006). AIDS. 1999;13(4):479-86.

Naidoo M, Naidoo KL, Lombard C, Desmond AC, Clark R, Rooney JF, et al. In-utero exposure to tenofovir disoproxil fumarate pre-exposure prophylaxis and growth metrics in HIV unexposed breastfed infants in South Africa: a post hoc analysis of the CAP 016 PrEP in pregnancy RCT. Frontiers in Pediatrics. 2024;12:1447173.

Newell ML, Parazzini F, Mandelbrot L, Peckham C, Semprini A, Bazin B, et al. A randomised trial of mode of delivery in women infected with the human immunodeficiency virus. British Journal of Obstetrics and Gynaecology. 1998;105(3):281EP - 5.

Sakai H, Kawata R, Adhikari R, Thapa YO, Bhandari TR. Effectiveness of art-based health education on anemia and health literacy among pregnant women in Western Nepal: A randomized controlled trial. PLoS ONE. 2024;19(9):e0281789.

Shapiro RL, Kitch D, Ogwu A, Hughes MD, Lockman S, Powis K, et al. HIV transmission and 24-month survival in a randomized trial of HAART to prevent MTCT during pregnancy and breastfeeding in Botswana. AIDS. 2013;27(12):1911-20.

Sperling RS, Shapiro DE, McSherry GD, Britto P, Cunningham BE, Culnane M, et al. Safety of the maternal-infant zidovudine regimen utilized in the Pediatric AIDS Clinical Trial Group 076 study. AIDS. 1998;12(14):1805

EP - 13.

# Appendix 13: Corrected covered area

## Appendix 13.1: Overall corrected covered area

| Primary Publication | Beck 2023 | Boering 2025 | Cowdell 2022 | Cowdell 2025 | Hey 2025 | Portwood 2022 | Sexton 2023 | Uthman 2017 | Wedi 2016 |
| --- | --- | --- | --- | --- | --- | --- | --- | --- | --- |
| Aaron 2012 | X |  | X |  |  |  | X |  |  |
| Adam 2016 |  | X |  |  |  |  |  |  |  |
| Adjorlolo 1991 |  |  |  |  |  |  |  |  | X |
| Ai-Jie 2013 |  |  |  |  |  | X |  |  |  |
| Albert 2020 | X |  | X |  |  | X |  |  |  |
| Alger 1993 |  |  |  |  |  |  |  |  | X |
| Aniji 2013 |  |  |  |  |  |  | X | X |  |
| Ayisi 2003 |  |  |  |  |  |  |  |  | X |
| Azria 2009 |  |  |  |  | X | X |  |  |  |
| Bailey 2013 |  | X |  |  |  | X |  |  |  |
| Bailey 2019 | X |  | X |  |  |  |  |  |  |
| Balogun 2018 |  |  |  |  | X | X |  |  |  |
| Benamor Teixeira 2020 | X |  |  |  |  |  |  |  |  |
| Bengtson 2020 |  | X |  |  | X |  |  |  |  |
| Bergstrom 1995 |  |  |  |  |  | X |  |  |  |
| Boer 2007 |  |  | X |  | X | X |  |  |  |
| Boyajian |  |  |  |  |  | X |  |  |  |
| Braddick 1990 |  |  |  |  |  |  |  |  | X |
| Bucceri 1997 |  |  |  |  |  |  |  |  | X |
| Bulterys 1994 |  |  |  |  |  |  |  |  | X |
| Caniglia 2018 |  |  |  | X |  |  |  |  |  |
| Carceller 2009 |  |  | X |  | X | X |  |  |  |
| Chagomerana 2017 |  |  |  |  |  | X | X |  |  |
| Chauhan 2021 | X |  |  | X |  |  |  |  |  |
| Chen 2012 | X | X | X |  | X | X | X | X |  |
| Chetty 2018 |  |  |  |  |  |  | X |  |  |
| Chibwesha 2016 |  |  |  |  |  | X |  |  |  |
| Coley 2001 |  |  |  |  |  |  |  |  | X |
| Cooper 2002 |  |  |  |  |  | X |  |  |  |
| Cotter 2006 |  |  |  |  |  | X |  |  |  |
| Da Souza 2000 |  |  |  |  |  | X |  |  |  |
| Dadabhai 2019 |  | X |  |  | X | X |  |  |  |
| Dale 2013 |  |  |  |  |  |  |  | X |  |
| Delicio 2018 | X |  | X |  |  |  |  |  |  |
| Djeha 2019 |  | X |  |  |  |  |  |  |  |
| Ejigu 2019 | X |  | X | X |  |  |  |  |  |
| Ekouevi 2008 |  | X |  |  |  |  | X |  |  |
| Ellis 2002 |  |  |  |  |  |  |  |  | X |
| European Pregnancy And Paediatric Hiv Cohort Collaboration Eppicc Study Group 2019 |  |  |  | X |  |  |  |  |  |
| Ezeaka 2009 |  |  |  |  |  |  |  |  | X |
| Ezechi 2012 | X |  | X |  |  |  |  |  |  |
| Favarato 2018 | X |  | X |  |  |  | X |  |  |
| Favarato 2019 | X |  | X | X |  |  |  |  |  |
| Floridia 2014 |  |  | X |  |  |  |  |  |  |
| Floridia 2018 |  |  | X | X |  |  |  |  |  |
| Floridia 2020 | X |  |  |  |  |  |  |  |  |
| Friis 2004 |  |  |  |  |  |  |  |  | X |
| Gagnon 2016 |  |  |  |  | X |  |  |  |  |
| Garcia-Otero 2019 |  |  |  |  |  | X |  |  |  |
| Gibb 2012 |  |  |  | X |  |  |  |  |  |
| Goetghebuer 2019 |  | X |  |  |  | X |  |  |  |
| Habib 2008 |  |  |  |  |  |  |  |  | X |
| Haeri 2009 |  |  |  |  |  | X |  |  |  |
| Halsey 1990 |  |  |  |  |  |  |  |  | X |
| Hernandez 2017 |  |  |  |  |  | X |  |  |  |
| Hu 2019 |  | X |  |  |  | X |  |  |  |
| Jiang 2022 |  |  |  | X |  |  |  |  |  |
| Johnstone 1996 |  |  |  |  |  |  |  |  | X |
| Joseph 2011 |  |  |  |  |  | X |  |  |  |
| Jumare 2019 |  |  |  |  |  | X |  |  |  |
| Kakkar 2015 |  |  | X | X |  | X |  |  |  |
| Kowalska 2003 |  | X | X |  |  | X | X |  |  |
| Ladner 1998 |  |  |  |  |  |  |  |  | X |
| Lallemant 1989 |  |  |  |  |  |  |  |  | X |
| Latham 2022 | X |  |  |  |  |  |  |  |  |
| Lepage 1991 |  |  |  |  |  |  |  |  | X |
| Leroy 1998 |  |  |  |  |  |  |  |  | X |
| Li 2016 |  | X |  |  |  | X | X | X |  |
| Li 2020 |  | X |  |  |  | X |  |  |  |
| Liff 2020 |  |  |  |  |  | X |  |  |  |
| Lopez 2012 |  |  |  |  |  | X |  |  |  |
| Lopez 2015 | X |  | X |  |  |  | X |  |  |
| Machado 2009 | X |  | X |  |  |  | X | X |  |
| Malaba 2017 |  | X |  |  | X | X | X |  |  |
| Malaba 2018 |  |  |  |  | X | X |  |  |  |
| Malaba 2021 |  | X |  |  |  |  |  |  |  |
| Mantelbrot 1998 |  |  |  |  |  | X |  |  |  |
| Mantelbrot 2015 |  |  |  |  |  |  | X | X |  |
| Marazzi 2011 |  | X |  |  |  | X |  |  |  |
| Markson 1996 |  |  |  |  |  |  |  |  | X |
| Martin 2007 |  |  |  |  |  | X |  |  |  |
| Matheson 1995 |  |  |  |  |  | X |  |  |  |
| Maynard 1990 |  |  |  |  |  |  |  |  | X |
| Mehta 2019 |  |  |  |  | X | X |  |  |  |
| Mmiro 1993 |  |  |  |  |  |  |  |  | X |
| Montgomery-Taylor 2015 |  |  | X |  |  | X |  |  |  |
| Moodley 2016 |  | X |  | X | X | X |  |  |  |
| Moseholm 2019 |  |  |  |  |  | X |  |  |  |
| Musana 2009 |  |  |  |  |  |  |  |  | X |
| Mwanyumba 2001 |  |  |  |  |  |  |  |  | X |
| Ndirangu 2012 |  |  |  |  |  |  |  |  | X |
| Noble 2005 |  |  |  |  |  |  |  |  | X |
| Olagbuji 2010 |  | X |  |  | X | X |  |  |  |
| Oomeer 2015 |  |  |  |  |  |  | X |  |  |
| Patel 2005 |  |  |  |  |  |  |  | X |  |
| Patel 2022 | X |  |  |  |  |  |  |  |  |
| Perry 2016 |  |  | X |  |  |  |  |  |  |
| Phiri 2015 |  |  |  |  |  | X |  |  |  |
| Pintye 2017 |  |  | X |  |  |  |  |  |  |
| Piske 2021 | X |  |  |  |  |  |  |  |  |
| Ramokolo 2017 |  | X |  |  | X | X | X |  |  |
| Ransom 2013 |  |  |  | X |  |  |  |  |  |
| Rempis 2017 |  | X |  |  | X | X | X |  |  |
| Rollins 2007 |  |  |  |  |  |  |  |  | X |
| Rough 2018 |  |  |  |  |  |  | X |  |  |
| Rubin 2011 |  | X |  |  | X | X |  |  |  |
| Ryder 1989 |  | X |  |  | X | X |  |  |  |
| Samuel 2014 |  |  |  |  |  |  | X |  |  |
| Santosa 2019 |  | X |  |  | X | X |  |  |  |
| Saums 2019 |  | X |  |  | X | X |  |  |  |
| Schulte 2004 |  |  | X |  |  |  |  |  |  |
| Schulte 2007 |  |  |  |  |  | X |  |  |  |
| Sebitloane 2017 |  | X |  |  | X | X | X |  |  |
| Seidel 2020 |  |  |  | X |  |  |  |  |  |
| Selwyn 1989 |  |  |  |  |  |  |  |  | X |
| Shapiro 2010 | X |  | X |  |  |  |  |  |  |
| Short 2014 | X | X | X |  |  | X | X |  |  |
| Siberry 2012 |  |  |  | X |  |  |  |  |  |
| Sibiude 2012 |  |  | X |  |  |  |  |  |  |
| Sibiude 2018 |  |  | X | X |  |  |  |  |  |
| Sibiude 2021 | X |  |  |  |  |  |  |  |  |
| Silverman 2010 |  | X |  |  |  | X |  |  |  |
| Simonds 1998 |  |  |  |  |  | X |  |  |  |
| Smith 2016 |  |  | X |  |  |  |  |  |  |
| Snijdewind 2018 | X | X |  |  | X | X | X |  |  |
| Sutton 1999 |  |  |  |  |  |  |  |  | X |
| Szyld 2006 | X |  | X |  |  |  |  |  |  |
| Taha 1995 |  |  |  |  |  |  |  |  | X |
| Tan 2023 |  | X |  |  |  |  |  |  |  |
| Tariq 2011 |  |  |  | X |  |  |  |  |  |
| Tennerman 1994 |  |  |  |  |  |  |  |  | X |
| Thimm 2022 |  |  |  | X |  |  |  |  |  |
| Thorne 2000 |  |  |  |  |  |  |  | X |  |
| Tiam 2019 |  | X |  |  | X | X |  |  |  |
| Ticconi 2003 |  |  |  |  |  |  |  |  | X |
| Tookey 2016 |  |  |  | X |  |  |  |  |  |
| Townsend 2007 |  |  | X |  |  |  | X |  |  |
| Townsend Ecs 2010 |  |  |  |  |  | X |  |  |  |
| Townsend Nshpc 2010 |  |  |  |  |  | X |  |  |  |
| Tuomala 2002 |  |  |  |  |  | X |  |  |  |
| Van Den Broek 2014 |  |  |  |  |  |  |  |  | X |
| Van Der Merwe 2011 | X |  | X |  |  | X |  |  |  |
| Vannappagari 2015 |  |  |  | X |  |  |  |  |  |
| Vigano 2011 |  |  |  | X |  |  |  |  |  |
| Von Linstow 2010 |  |  |  |  |  | X |  |  |  |
| Watts 2013 | X |  | X |  |  | X |  |  |  |
| Williams 2013 |  |  | X |  |  |  |  |  |  |
| Yu 2012 | X |  |  |  |  | X |  |  |  |
| Zash 2016 |  |  |  | X |  |  | X | X |  |
| Zash 2017 | X | X | X | X | X |  |  |  |  |
| Zash 2018 | X | X |  |  | X | X | X |  |  |
| Zash 2019 | X |  |  |  |  |  |  |  |  |
| Zash 2021 | X |  |  |  |  |  |  |  |  |
| Ziske 2013 |  |  |  |  |  | X |  |  |  |

## Appendix 13.2 Between-study corrected coverage area

| Study | Beck 2023 | Boering 2025 | Cowdell 2022 | Cowdell 2025 | Hey 2025 | Portwood 2022 | Sexton 2023 | Uthman 2017 | Wedi 2016 |
| --- | --- | --- | --- | --- | --- | --- | --- | --- | --- |
| Beck 2023 |  | 9.1% | 38.9% | 8.7% | 8.2% | 9.5% | 17.8% | 5.6% | 0.0% |
| Boering 2025 |  |  | 3.3% | 4.0% | 44.7% | 34.3% | 25.0% | 5.3% | 0.0% |
| Cowdell  2022 |  |  |  | 12.8% | 7.7% | 11.8% | 16.7% | 5.1% | 0.0% |
| Cowdell 2025 |  |  |  |  | 4.7% | 2.4% | 2.3% | 3.5% | 0.0% |
| Hey  2025 |  |  |  |  |  | 31.8% | 16.3% | 3.1% | 0.0% |
| Portwood  2022 |  |  |  |  |  |  | 14.5% | 2.9% | 0.0% |
| Sexton 2023 |  |  |  |  |  |  |  | 22.2% | 0.0% |
| Uthman 2017 |  |  |  |  |  |  |  |  | 0.0% |
| Wedi 2016 |  |  |  |  |  |  |  |  |  |
